# Identification of Tengfu Jiangya Tablet Target Biomarkers with Quantitative Proteomic Technique

**DOI:** 10.1155/2017/7594805

**Published:** 2017-03-20

**Authors:** Jingwen Xu, Yunlun Li, Shijun Zhang, Haiqiang Jiang, Nan Wang, Haiqing Lin

**Affiliations:** ^1^Shandong University of Traditional Chinese Medicine, 4655 Daxue Road, Changqing District, Jinan, Shandong, China; ^2^Affiliated Hospital of Shandong University of Traditional Chinese Medicine, 16369 Jingshi Road, Lixia District, Jinan, Shandong, China; ^3^Affiliated Hospital of Shandong Academy of Medical Sciences, 38 Shadowless Hill Road, Tianqiao District, Jinan, Shandong, China; ^4^Shandong Tumor Hospital, 440 Jiyan Road, Jinan, Huaiyin District, Shandong, China

## Abstract

Tengfu Jiangya Tablet (TJT) is a well accepted antihypertension drug in China and its major active components were* Uncaria total alkaloids* and* Semen Raphani soluble alkaloid*. To further explore treatment effects mechanism of TJT on essential hypertension, a serum proteomic study was performed. Potential biomarkers were quantified in serum of hypertension individuals before and after taking TJT with isobaric tags for relative and absolute quantitation (iTRAQ) coupled two-dimensional liquid chromatography followed electrospray ionization-tandem mass spectrometry (2D LC-MS/MS) proteomics technique. Among 391 identified proteins with high confidence, 70 proteins were differentially expressed (fold variation criteria, >1.2 or <0.83) between two groups (39 upregulated and 31 downregulated). Combining with Gene Ontology annotation, KEGG pathway analysis, and literature retrieval, 5 proteins were chosen as key target biomarkers during TJT therapeutic process. And the alteration profiles of these 5 proteins were verified by ELISA and Western Blot. Proteins Kininogen 1 and Keratin 1 are members of Kallikrein system, while Myeloperoxidase, Serum Amyloid protein A, and Retinol binding protein 4 had been reported closely related to vascular endothelial injury. Our study discovered 5 target biomarkers of the compound Chinese medicine TJT. Secondly, this research initially revealed the antihypertension therapeutic mechanism of this drug from a brand-new aspect.

## 1. Introduction

Characterized by functional and structural vascular abnormalities, essential hypertension (EH) is a global noncommunicable epidemic disease. EH always results in complications of various organ systems [1, 2]. Currently, the incidence of EH was found increasing year by year [3]. For this reason, development of new antihypertension drugs attracted much attention from global researchers.

Characterized by “multiple-effects, multiple-targets, and multiple-compounds,” Traditional Chinese medicine (TCM) was widely accepted in eastern countries [4]. Tengfu Jiangya Tablet (TJT) is a registered TCM (number Z20110021, Shandong provincial food and Drug Administration). This Chinese patent medicine was developed on the basis of TCM theory and modern preparation technologies. TJT is one of the best selling drugs which was taken daily by many patients for controlling hypertension. TJT has been widely applied in China and well accepted for its excellent bioactivities and efficacy in clinical practices. The main raw materials of TJT were* Uncaria rhynchophylla* and* Semen Raphani*. Previous studies have revealed that main effective components of TJT were* Uncaria total alkaloids* (including* Rhynchophylline* and* Isorhynchophylline*) and* Semen Raphani soluble alkaloid* (mainly sinapine thiocyanate) [5]. Pleiotropic effects of TJT included improved endothelial function, antioxidant and anti-inflammatory [6–8].

TJT was found lacking systemic pharmacomechanism, a problem with the application. Although previous studies revealed that high blood pressure (BP) was improved with TJT, no definite evidence was found with such molecular mechanism as hypertension associated target biomarkers in blood circulation. Proteins are direct executors in vital activities, and the expression level of proteins varies in all stages of life activities. Proteomics could dynamically reveal genetic manipulation, endogenous changes, and exogenous stimuli [9]. Fortunately, based on modern systematic theory, proteomics allows for an integrity identification of physiological variations. The specific target biomarkers in proteomics were associated with pathogenesis process, which was varied in individuals with different disease development stages or after drug intervene [10, 11].

**Figure 1:**
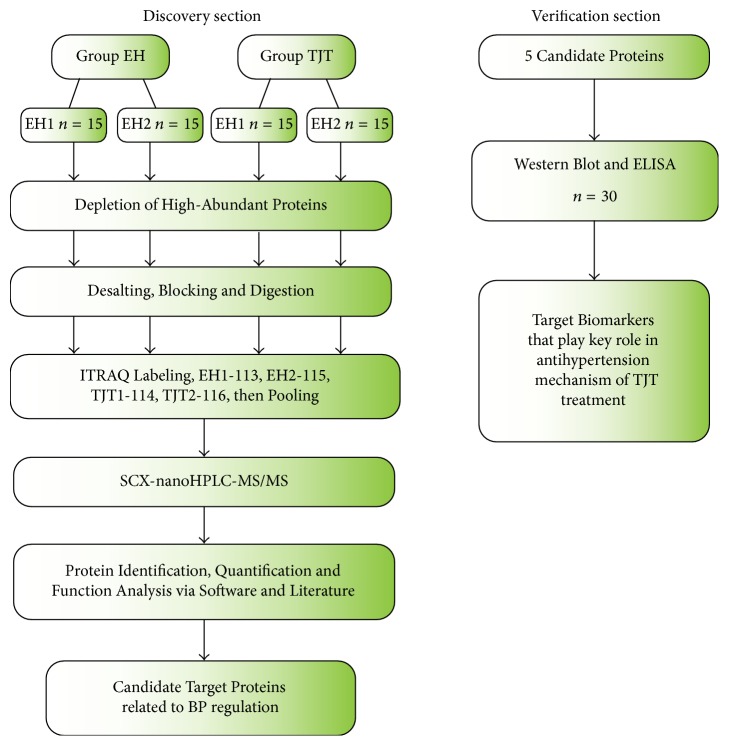
Overview of the workflow for exploring and verifying target biomarkers. In the discovery section, the two groups were randomly divided into two subgroups for biological repetition (EH1, *n* = 15; EH2, *n* = 15; TJT1, *n* = 15; TJT2, *n* = 15). Then, in verification section, the remaining 17 samples and 13 samples involved in the discovery section were applied in verification.

In this study, it was to screen out the effective target proteins, through which TJT made effects on protecting vascular endothelial function and modulating BP. This study consists of two parts: discovery section and verification section, and the workflow is demonstrated in detail in Figure 1. Firstly, in the discovery section, quantitative proteomic technique (iTRAQ coupled 2D-LC-MS/MS) was employed to identify and quantify the expression level of proteins between EH individuals before and after TJT treatment. Gene Ontology (GO) analysis, KEGG, and literature retrieval were employed to select out candidate target proteins closely related to blood pressure regulation. Secondly, in the verification section, candidate target proteins were validated by ELISA and Western Blot.

## 2. Material and Methods

### 2.1. Preparation of Drugs

Tengfu Jiangya Tablet (TJT) was provided by Pharmacy Department of the Affiliated Hospital of Shandong University of Traditional Chinese Medicine (Jinan, Shandong). The main effective components in TJT were quantitatively determined according to the quality standard established in previous studies. The content of* Rhynchophylline* was determined to be higher than 1.182 mg while less than 1.444 mg in each piece; the content of* sinapine thiocyanate* was more than 6.001 mg and less than 7.335 mg [5].

### 2.2. Patient Groups and Drug Treatment

EH patients were recruited from the Outpatient department of Cardiology in the Affiliated Hospital of Shandong University of Traditional Chinese Medicine (Shandong, China). It was reviewed and approved by the Ethics Committee of the Affiliated Hospital of Shandong University of Traditional Chinese Medicine. The protocol was approved by institutional review board and written informed consent was obtained from all patients. All EH patients were defined and screened following the criteria of* Hypertension Prevention and Cure Guideline* (in Chinese, 2010). 47 patients (age range: 43–62, average 55.87 ± 8.41) met the BP criteria (140 mmHg ≤ SBP ≤ 179 mmHg or 90 mmHg ≤ DBP ≤ 109 mmHg) after a washout period of two weeks.

Patients were treated with TJT orally (taking 2 tablets three times per day) for 4 weeks. Such clinical parameters as systolic blood pressure and diastolic blood pressure (Supplementary Figure 1 in Supplementary Material available online at https://doi.org/10.1155/2017/7594805) were determined before and after TJT treatment. The morning fasting venous blood of all included patients was obtained before (EH group) and after TJT treatment for 4 weeks (TJT group).

Patients with secondary hypertension, diabetic mellitus, uncontrolled hypertension, heart failure, mental disorder, renal dysfunction, or liver disease and women with pregnancy and breastfeeding and planning to be pregnant were excluded in this study as well.

### 2.3. Sample Preparation and Distribution

Blood samples were collected and allowed to clot in vacuum tubes without anticoagulant. Samples were centrifuged at 4000 rpm for 10 min under 4°C to separate serum. Serum samples were immediately dispensed into the sterile EP tubes and stored at −80°C. Hemolysis was eliminated in the samples as well [12].

Serum samples of 30 patients were randomly selected out from 47 patients for the discovery section. The remaining 17 patients and 13 patients involved in the discovery section were jointly analyzed in verification section. In verification section, on the one hand, the remaining 17 patients were tested to confirm the generalizability of previous result. On the other hand, we tested the 13 patients again to prove the previous result was not accidental.

Clinical parameters of EH patients between discovery section (*n* = 30) and verification section (*n* = 30) were listed in Supplementary Table 1. To minimize the interferences from complex samples, the highly abundant proteins were depleted with ProteoMinerTM Kits (Bio-Rad Laboratories, Hercules, CA, USA).

### 2.4. Trypsin Digestion, ITRAQ Labeling, and SCX Fractionation

To reduce individual differences, a pooled sample was prepared from the mixture of 15 samples (with the equal amount) in groups EH and TJT, respectively. Thus, there were 4 pooled samples (Groups EH1, EH2, TJT1, and TJT2) which would be analyzed in following experiment. The pooled samples were reduced with DTT (10 mmol/L) and alkylated with IAM (55 mmol/L). Then, samples were precipitated by cold acetone at −20°C overnight. After centrifugation at 30,000*g* under 4°C, the pellet was dissolved in 0.5 M TEAB (Applied Biosystems, Milan, Italy) and sonicated in ice. After centrifuging, total protein concentration of the supernatant was measured with Bradford method (Solarbio, Beijing, China) [13]. Proteins (with the amount of 100 *μ*g) of each sample were digested with trypsin and labeled with iTRAQ reagents (Applied Biosystems, USA). EH1 and EH2 were labeled with iTRAQ 113 and 115 reagent, respectively; TJT1 and TJT2 were each labeled with iTRAQ 114 and 116 reagent.

The labeled samples were resolved into 20 fractions with an Ultremex SCX column containing 5 *μ*m particles (Phenomenex, USA). The eluted fractions were then desalted with a Strata X C18 column (Phenomenex, USA) and dried under vacuum [14].

### 2.5. LC-ESI-MS/MS Analysis Based on Q EXACTIVE

Each 20 SCX separated fraction was resuspended in buffer A (5% ACN, 0.1% FA). After mixing, solution was centrifuged and precipitation was collected. The supernatant was continually separated by LC-20AD nano HPLC (Shimadzu, Kyoto, Japan). Fractions were transferred into C18 column for gradient elution. Samples were first loaded at 8 *μ*L/min for 4 min, followed by a gradient elution program: 0–4 min, 5% B (98% ACN, 0.1% FA); 4–48 min, 2%–35% B; 48–50 min, 35%–80% B; 50–54 min, 80% B; 54–55 min, 80%–5% B; the running speed was 300 nL/min.

The obtained peptides were subjected to nanoelectrospray ionization followed by tandem mass spectrometry (MS/MS) in an Q EXACTIVE (Thermo Fisher Scientific, San Jose, CA) coupled online to the HPLC. Program settings: peptides detect resolution, 70000; collision energy, 27 V; ion fragments detect resolution, 17500; electrospray voltage, 1.6 kV; *m*/*z* scan range for MS, 350 to 2000 Da; *m*/*z* scan range for MS2, 100–1800 Da.

### 2.6. Database Screening

Raw data was acquired from the Orbitrap and converted into MGF files with Proteome Discoverer 1.2 (PD 1.2, Thermo). The data was searched in MGF file. Proteins identification was performed with Mascot search engine (Matrix Science, London, UK; version 2.3.02) against database including 143,397 sequences (Homo sapiens, release-2014_12).

The Mascot search criteria were set with following criteria: precursor mass tolerance: 20 ppm; fragment ions tolerance: 0.05 Da; enzyme: trypsin; fixed modifications: Carbamidomethyl (C), iTRAQ 8plex (N-term), iTRAQ 8plex (K); variable modifications: Gln- >pyro-Glu (N-term Q), Oxidation (M), Deamidated (NQ); maximum missed cleavage sites: 1; instrument type: default. The decoy database search was set to avoid false positives in Mascot searches. Only peptides with Mascot probability analysis greater than “identity” (95% confidence interval) were counted as identified. And at least one unique peptide was involved in each confidently identified protein.

Only protein with fold change meeting the criteria (>1.2 or <0.83) and *p* values < 0.05 was considered as protein with significant expression differences.

### 2.7. Informatics Analysis

After proteins were identified, the functional classifications (molecular function, cellular component, and biological process) were annotated by GO database. The pathway annotation was performed with KEGG database. Protein interaction network mode was created with the String database.

### 2.8. ELISA and Western Blot

To confirm the identification and variation of candidate proteins, ELISA was employed to quantify the concentrations of these candidates. The remaining 17 samples and 13 samples involved in the discovery section were included in this verification section. Human Retinol binding protein 4 (RBP4), Kininogen 1 (KNG1), Keratin 1 (KRT1) ELISA kit (Cusabio Biotech, Wuhan, Hubei, China), Human Serum amyloid protein A (SAA) ELISA kit (Abcam, Cambridge, MA, USA), and Human Myeloperoxidase (MPO) ELISA kit (Uscn Life Science, Wuhan, Hubei, China) were applied to determine the concentration of proteins in each serum sample in validation set (30 samples in EH group and 30 samples in TJT treated group). Measurements of each sample were performed in duplicate according to manufacturer's instructions. The concentration of bradykinin, which was the downstream product of KNG1, was also detected with ELISA kit (Cusabio Biotech, Wuhan, Hubei, China). Different expressed proteins KNG1 and RBP4 were closely related to EH; thus the two proteins were also verified by Western Blot to confirm the results obtained by iTRAQ proteomic analysis.

50 *μ*g serum protein of each group was separated in 15% w/v SDS-PAGE and transferred to PVDF membrane (0.22 *μ*m) with semidry transfer system (BIO-RAD Trans-Blot Turbo, US). After blocking and washing, membranes were incubated with antibody (1 : 1000) (Abcam, Cambridge, UK) overnight at 4°C. Then the membranes were washed and incubated with second antibody (1 : 20000, HRP labeled goat anti-rabbit IgG) (ZSGB-BIO, China) at room temperature for 2 h. After washing, the blots were detected with ECL solution (Millipore, GER). Transferrin was chosen as internal marker (Rabbit anti-Transferrin antibody, bs-2052R, Beijing, China) [15]. Finally, the intensity of protein bands was analyzed by Quantity One software (Bio-Rad, USA). The experiment was performed in triplicate.

### 2.9. Statistical Analysis

All the measurement data were expressed as Mean ± SD. Statistical analysis was performed with SPSS16.0 statistical package. For comparisons between groups, all comparative data was analyzed with independent *T*-test or Chi-square test. A threshold of *p* < 0.05 indicated significant difference.

## 3. Results

### 3.1. Protein Quantification and Candidate Proteins Identification

In the discovery section, serum proteins were analyzed and compared in EH and TJT treatment groups. A total of 2305 peptides were identified with a false discovery rate of 1%, while 391 proteins were identified with 95% confidence interval (Supplementary Table 2). All of these proteins were reliably quantified. Among them, 70 proteins (Supplementary Table 3) were identified to be differentially expressed, with statistical significance (*p* < 0.05). With a fold change threshold of 20% (ratios > 1.20 or <0.83), 31 proteins were upregulated (EH-Vs-TJT) and 39 proteins were downregulated.

**Figure 2:**
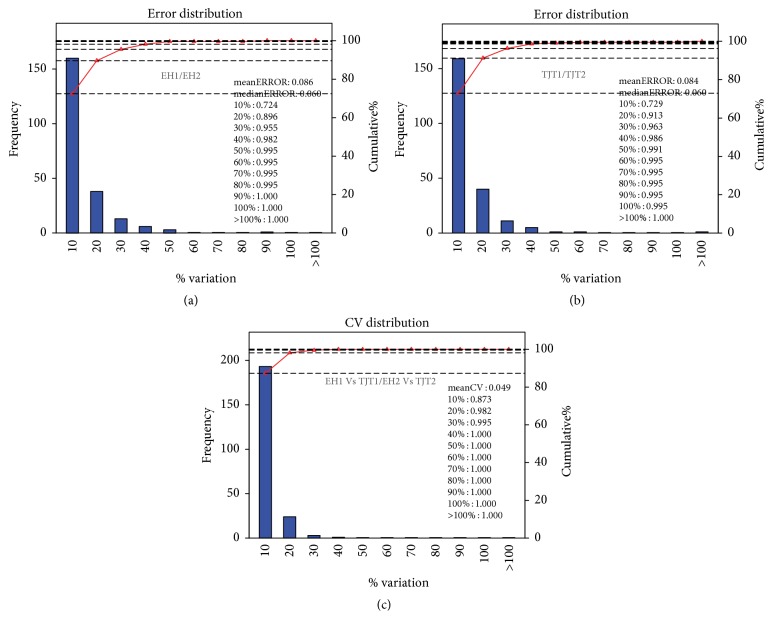
Reproducibility of proteomic analysis. Abscissa represented different variation levels, the left ordinate represented the number of quantitative proteins at different variation level, and the right ordinate represented the accumulation ratio of total quantitative protein at different variation level. (a and b) indicated comparison of intragroup (EH1/EH2, TJT1/TJT2), while (c) indicated the reproducibility between two comparison groups (EH1 versus TJT1/EH2 versus TJT2).

In the discovery section of this study, one biological repetition was designed to reduce individual differences and enhance the confidence of this study. In sample preparation, the serums in each group were randomly divided into two subgroups. Then the samples in each subgroup were pooled and 4 subgroups were obtained: EH1 (iTRAQ labeled 113), EH2 (iTRAQ labeled 115), TJT1 (iTRAQ labeled 114), and TJT2 (iTRAQ labeled 116). The results of both intragroup replicate and repetitive assessment between comparison groups showed a good repeatability (Figure 2). There were only two groups involved in our study, we set up a replication to eliminate random difference and enhance the credibility of this study. Fortunately, the mean error within group is only 8.6% (EH1/EH2) and 8.4% (TJT1/TJT2). Meanwhile in both two comparable groups, the variation range of more than 95% identified proteins was below 30%. And the CV values were below 30% (coverage ratio > 95%). All the replication data proved that the quantification and comparison results were reliable and repeatable in this experiment.

### 3.2. Bioinformatics

**Figure 3:**
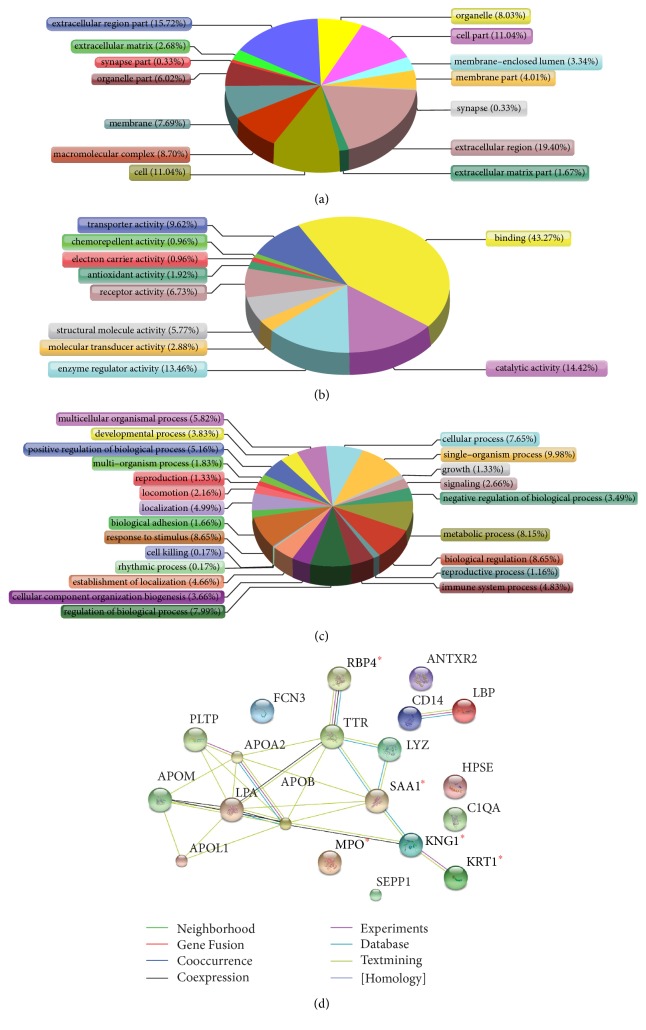
Bioinformatics analysis of differentially expressed proteins. GO analysis results showed cellular component (a), molecular function (b), and biological process (c). STRING analysis indicated visualization of protein-protein interactions for 20 candidate proteins (d). The notes for different color lines were in the bottom of (d). In addition, 5 candidate biomarkers verified by ELISA and Western Blot were marked with *∗*.

The datasets of identified proteins were searched for exploring potential relevant biomarkers. Firstly, the bioinformation function of 70 differentiated proteins was analyzed. GO functional annotation was employed to determine the cellular component, molecular function, and biological process of these proteins (Figure 3). The cellular component annotation showed that most of the differentiated proteins were located in extracellular region (19.40%), extracellular region part (15.72%), cell (11.04%), and cell part (11.04%). Molecular function annotation revealed that the dominant functions of these proteins were binding (43.27%), catalytic activity (14.42%), enzyme regulator activity (13.46%), and transporter activity (9.62%). However, the biological process of these proteins was differed. The major biological processes of these proteins were biological regulation (8.65%), response to stimulus (8.65%), metabolic process (8.15%), and regulation of biological process (7.99%).

The candidate target biomarkers should be associated with the regulation of vascular function. Thus, the candidates must be secretary proteins with regulation functions, acting as binding receptors or catalyst in relevant biological process. In addition, meanwhile, KEGG database and pertinent literature were searched for these differentiated proteins. Finally, 20 candidate proteins (Table 1) were selected out as they were closely associated with vascular endothelial function or reported to make effects in EH pathological mechanism.

In order to explore the interactions of these 20 differentiated proteins during TJT treatment, String database was also searched for the protein-protein interaction network. String map (Figure 3(d)) displayed the genetic constitution, physical, and functional interaction among these proteins, from which 5 proteins (proteins KNG1, KRT1, MPO, RBP4, and SAA) were located in central of the network and they were selected as target candidates in TJT treatment process.

### 3.3. Validation of Candidate Proteins

All of these 5 candidate proteins were verified individually with ELISA. The concentrations of KNG1 in the EH and TJT group were 117.43 ± 33.61 ug/ml and 208.57 ± 41.19 ug/ml, respectively. The concentrations of KRT1 in the EH and TJT group were 0.53 ± 0.13 ng/ml and 1.16 ± 0.33 ng/ml, respectively. The concentrations of MPO in the EH and TJT group were 91.91 ± 27.65 ng/ml and 35.14 ± 9.33 ng/ml, respectively. The concentrations of RBP4 in the EH and TJT group were 18.51 ± 3.60 ug/ml and 12.42 ± 1.78 ug/ml. The concentrations of SAA in the EH and TJT group were 36.38 ± 5.54 ng/ml and 28.20 ± 3.97 ng/ml.

In this study, the levels of serum protein KNG1 and KRT1 were increased after TJT treatment. Meanwhile, both KEGG analysis and literature retrieval revealed that the two proteins were involved in kallikrein-kinin system (KKS). KNG1 is the precursor of bradykinin, while KRT1 is an assistant protein during the production of bradykinin. So it is supposed that the level of bradykinin could be raised by TJT treatment, resulting in lowered blood pressure through KKS pathway. In addition, to further verify our speculation, the levels of bradykinin in two groups were tested with ELISA. The concentrations of bradykinin in EH and TJT individuals were 1526.29 ± 332.35 pg/ml and 4329.18 ± 635.16 pg/ml. After TJT treatment, it was actually increased.

**Figure 4:**
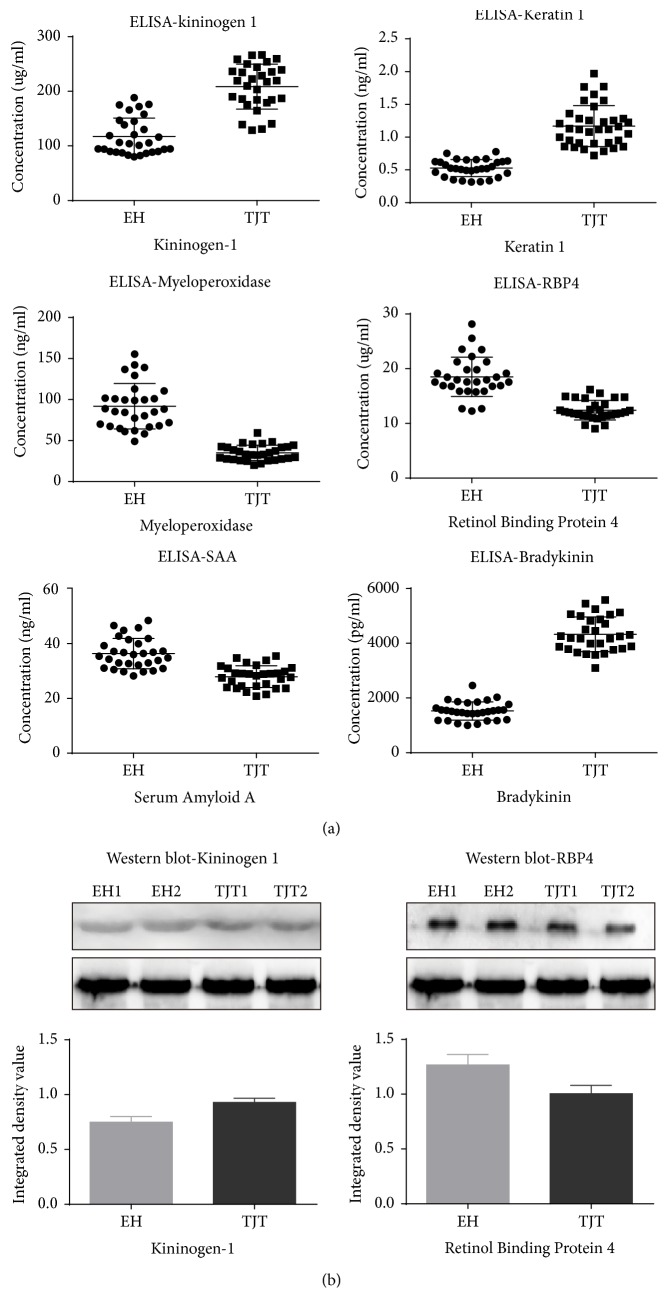
Validation of Kininogen-1, Keratin 1, Myeloperoxidase, Retinol binding protein 4, Serum amyloid A protein, and bradykinin in serum. (a) Levels of these candidate biomarkers and downstream substance were measured by ELISA in serum of EH patients (*n* = 30) and TJT treated patients (*n* = 30). *p* values were calculated with ANOVA test (^*∗*^*p* = 0.000). (b) With Western Blot results, the expression of Kininogen 1 and Retinol binding protein 4 was significantly differentiated between two groups. Transferrin was applied as loading control.

The variations of serum KNG1 (48 KDa) and RBP4 (23 KDa) were verified with Western Blot. It aimed to demonstrate a visible expression profile. The intensity image and grayscale histogram of two proteins in EH and TJT group were provided (Figure 4). The verification results on both ELISA and Western Blot were consistent with iTRAQ proteomics obtained in discovery section.

## 4. Discussion

**Figure 5:**
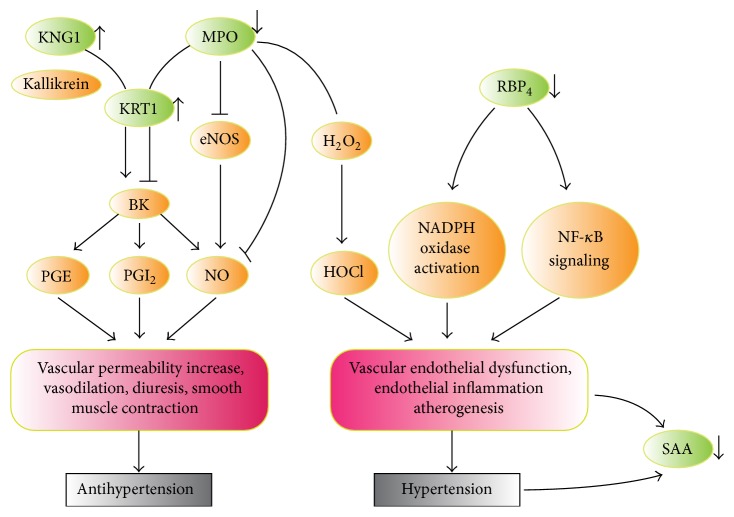
This map directly summarized the pathway of 5 target proteins (green ovals). The arrowheads represented variation trends of target protein after TJT treatment. KNG1, KRT1, and MPO were involved in bradykinin production; meanwhile MPO and RBP4 played vital role in vascular oxidative and inflammatory injury. SAA was an acute-phase marker for vascular injury.

Tengfu Jiangya Tablet (TJT) is a well accepted and widely applied traditional Chinese compound prescription. The present study has demonstrated that it was effective in treating essential hypertension (Supplementary Figure 1). To gain insights into the antihypertension therapeutic mechanism of TJT, it analyzed the proteomic variation of EH patients before and after TJT intervention with iTRAQ quantitative proteomic technique. Then, five target proteins were identified to be closely related to EH pathogenesis, which were also verified with immunology validation. Finally, the correlated function pathways involving those target proteins were recognized as pharmacomechanism pathways (Figure 5) for high blood pressure treatment efficacy of TJT.

In our study, iTRAQ proteomic analysis was employed to explore and quantify dysregulated proteins. It has been a mature and high-throughput proteomic technique comparing to other proteomic methods [16]. The relative quantification of eight groups could be achieved simultaneously; thus the requirement of repeat could also be fulfilled in the analysis of less than four groups. The simultaneous analysis would result in better repeatability. The results of both intragroup replicate and repetitive assessment between comparisons in our results showed a good repeatability.

During these years, basing on the antihypertension effect of TJT, our research team has been dedicated to studying the pharmacological of TJT on molecular biological and metabonomics. We have verified that TJT can decrease serum endothelin (ET-1) and increase the level of NO; to some extent, it also could reduce serum rennin and angiotensin II [7, 8]. The metabonomics displayed TJT could achieve antihypertension function by improving NO production and an extra cardiovascular role by amelioration of inflammatory state and vascular remodeling [5]. In this study, we discovered key five target biomarkers that TJT directly made effect on, which were KNG 1, KRT1, MPO, SAA, and RBP4.

KNG1 was a precursor of vasoactive kinins-high-molecular-weight kininogen (HMWK) and low-molecular-weight Kininogen (LMWK), which could release bradykinin from plasma or kidney glandular tissues [17, 18]. These cytokines were major components of kallikrein-kinin system (KKS), which were recognized as a key part in maintaining the homeostasis of normal blood pressure [19, 20]. In our previous study, KNG1-deficiency was identified as a key factor involved in the development of EH.

As we know, bradykinin would be released from KNG1 after the action of plasma Kallikrein; however, it should be noticed that bradykinin would only be completely released into blood or tissues by KNG 1 after binding to endothelium. Keratin 1 (KRT1), the highest-molecular-weight keratin (67 KDa), was an integral component of the multiprotein KNG receptor in endothelial cells. In blood vessels, bradykinin delivery must be regulated by the binding of KNG1 and endothelium, which largely depended on the binding function of KRT1 [21, 22]. It suggested that the bradykinin delivery would be influenced by level variation of KNG1 and KRT1 to a great extent.

In our study, a significant increase profile was observed in both KNG1 and KRT1 after TJT treatment. Meanwhile, the verification section further confirmed that the concentration of not only the two serum proteins but also bradykinin was raised after oral administration of TJT. Therefore, TJT would make antihypertension function through elevating the serum level of KNG1 and KRT1 or stimulating the activity of these two proteins.

Myeloperoxidase (MPO), a leukocyte-derived enzyme, was considered as an independent risk biomarker for indicating cardiovascular inflammation [23]. According to previous studies, vascular endothelial function could be damaged by MPO from three aspects: Firstly, normal EC function would be directly undermined by neutrophil MPO through oxidative stress (OS) [24]. Secondly, MPO would adhere to EC surface and serve as a major target-antigen for antineutrophil cytoplasmic autoantibodies [25]. But most of all, MPO would consume NO, which was the major dilating molecule produced by endothelium [26]. All the above factors have demonstrated the adverse influence of MPO on endothelial function.

Our research showed that the serum MPO before TJT treatment was significantly higher than that of after treatment. It implied that vascular could be protected for alleviating inflammatory injury and oxidative lesion. Astern et al.'s research indicated that MPO could cooperatively bind with KRT1 thus blocking the plasma Kallikrein cleavage site on KNG1 [27]. And our study shows that TJT could result in increased level of KNG1 and KRT1 and decreased production of MPO. Then the activation of the plasma KKS would be restored.

RBP4 is a 23 KDa protein and it was expressed in skeletal muscles and white adipose tissues [28]. Many studies suggested that RBP4 may be involved in the pathogenesis of early vascular damage during EH occurrence by participating in modulation of the atherosclerotic process [29]. The pathogenesis relationship between RBP4 and cardiovascular diseases was also studied. They found that insulin action would be inhibited by RBP4 in endothelial cells, leading to the damage of NO-dependent vasodilation [30]. Likewise, in our study, it showed that RBP4 level could be significantly decreased in serum after TJT treatment. It indicated potential protection effects on cardiovascular function in EH patients.

SAA belongs to apolipoprotein family, and it is mainly produced and stored in liver as well as adipose tissues. Once there was an infection or inflammation, SAA would be instantly released from hepatocytes to central venules [31]. Recently, many studies reported that inflammation plays critical pathogenetic roles in EH [32]. Besides, significant increased levels of SAA were observed in both SHR and EH patient serums [33]. In our study, serum SAA level in EH patients was significantly reduced after TJT treatment. This result was consistent with previous study that cardiovascular inflammation could be decreased after TJT treatment.

The TJT involved basic pharmacological pathway map in antihypertension process was provided (Figure 5), including classical Kallikrein system, vascular oxidative stress, and inflammation injury pathway. As shown in this map, the level of KNG1 could be increased by TJT, which competitively bound to KRT1 to improve the production of bradykinin. In addition, the level of MPO was decreased by TJT, alleviating the inhibition effects on both bradykinin and NO production. Meanwhile, the declined level of MPO and RBP4 after TJT treatment would make effects on antioxidative stress and anti-inflammation. Further in-depth study will be performed to study the mechanism and pathways through which TJT makes effects on these targets, respectively.

## 5. Conclusion

Based on iTRAQ proteomic analysis and immunological verification methods, five serum proteins were screened out as target biomarkers through which TJT made effects on EH. TJT could effectively improve vascular permeability and vasodilatation, while decreasing vascular inflammation. The potential mechanism may be increased bradykinin production and reduced vascular oxidative lesion. The quantitative proteomics provide an integrity view for disease pathogenesis and drug efficacy study.

## Supplementary Material

The comparison of blood pressure of EH patients (*n*=47) before and after TJT treatment. Both of the SBP and DBP were reduced significantly (*P*<0.01) after TJT treatment.

## Figures and Tables

**Table 1:** The molecular function, biological process, and expression levels of 20 candidate serum proteins quantified by iTRAQ technique.

Protein ID	Protein name	Molecular_function	Biological process	EH/TJT (mean)
sp|P18428	Lipopolysaccharide-binding protein	Receptor binding Lipoteichoic acid binding	Positive regulation of chemokine production Leukocyte chemotaxis involved in inflammatory; acute-phase response	0.789

tr|Q5SRP5	Apolipoprotein M	Lipid transporter activity Antioxidant activity	Response to glucose stimulus	0.6995

tr|F1C4A7	Monocyte differentiation antigen CD14	Lipoteichoic acid binding Lipopolysaccharide binding Opsonin receptor	Positive regulation of cytokine secretion Apoptotic process Inflammatory response	0.5385

tr|D6REX5	Selenoprotein P	Selenium binding	Response to oxidative stress	0.8125

tr|A0A024R1G8	Apolipoprotein L, 1	Lipid binding Protein binding Chloride channel activity	Chloride transport Cytolysis	0.342

tr|H6VRF8	Keratin 1	receptor activity protein binding	response to oxidative stress regulation of angiogenesis epidermis development	0.533

tr|Q1HP67	Lipoprotein, Lp(A)	Serine-type endopeptidase activity Protein domain specific binding	Negative regulation of cell proliferation Proteolysis Negative regulation of cell proliferation	0.4515

tr|C0JYY2	Apolipoprotein B	Receptor binding Enzyme binding		0.396

tr|D3DNU8	Kininogen 1	Receptor binding Zinc ion binding Heparin binding Cysteine-type endopeptidase inhibitor activity	Elevation of cytosolic calcium ion Positive regulation of renal sodium excretion; Smooth muscle contraction; Vasodilation; inflammatory response	0.796

tr|J3KPY9	Anthrax toxin receptor 2	Receptor activity Protein binding	Receptor-mediated endocytosis Artery morphogenesis Low-density lipoprotein particle remodeling	0.4945

tr|V9GYG9	Apolipoprotein A-II	Protein homodimerization activity binding	Response to glucocorticoid stimulus Protein oxidation Acute inflammatory response	0.609

tr|B3KUE5	Phospholipid transfer protein	Lipid binding	Vitamin E biosynthetic process Lipid metabolic process	0.441

sp|P05164	Myeloperoxidase	Peroxidase activity heme binding	Hydrogen peroxide catabolic process Oxidation-reduction process	1.868

tr|Q6UXM4	Ficolin 3	Receptor binding	Signal transduction Complement activation, lectin pathway	1.219

tr|F8VV32	Lysozyme C	Lysozyme activity	Inflammatory	1.5085

tr|Q5VY30	Retinol binding protein 4	Retinol transporter activity protein binding	Positive regulation of insulin secretion Positive regulation of immunoglobulin secretion; negative regulation of endopeptidase activity Peptidyl-glutamic acid carboxylation	1.856

tr|A0A024RDB8	Heparanase	Beta-glucuronidase activity heparanase activity	Vascular wound healing; regulation vascular endothelial growth factor production Glycosaminoglycan catabolic process	1.449

tr|D3DQX7	Serum amyloid A protein	G-protein coupled receptor binding	Acute-phase response; negative regulation of inflammatory response Neutrophil chemotaxis	1.2535

tr|X6RLJ0	Complement C1q subcomponent subunit A	Protein binding	Complement activation, classical pathway Cell-cell signaling	1.216

tr|A0A087WT59	Transthyretin	Protein binding	—	1.1655

## References

[1] Pierdomenico S. D., Di Nicola M., Esposito A. L. (2009). Prognostic value of different indices of blood pressure variability in hypertensive patients. *American Journal of Hypertension*.

[2] Mancia G., Laurent S., Agabiti-Rosei E. (2009). Reappraisal of European guidelines on hypertension management: a European Society of Hypertension Task Force documental. *Blood Pressure*.

[3] Writing Group of 2010 Chinese Guidelines for the Management of Hypertension (2011). 2010 Chinese guidelines for the management of hypertension. *Zhonghua Xin Xue Guan Bing Za Zhi*.

[4] Schmidt B. M., Ribnicky D. M., Lipsky P. E., Raskin I. (2007). Revisiting the ancient concept of botanical therapeutics. *Nature Chemical Biology*.

[5] Jiang H., Shen Z., Chu Y. (2015). Serum metabolomics research of the anti-hypertensive effects of Tengfu Jiangya tablet on spontaneously hypertensive rats. *Journal of Chromatography B: Analytical Technologies in the Biomedical and Life Sciences*.

[6] Shi Z., Lu Z., Zhao Y. (2013). Neuroprotective effects of aqueous extracts of Uncaria tomentosa: insights from 6-OHDA induced cell damage and transgenic Caenorhabditis elegans model. *Neurochemistry International*.

[7] Li Y., Yang W., Zhu Q., Yang J., Wang Z. (2015). Protective effects on vascular endothelial cell in N'-nitro-L-arginine (L-NNA)-induced hypertensive rats from the combination of effective components of Uncaria rhynchophylla and Semen Raphani. *BioScience Trends*.

[8] Li Y. L., Zhu M., Cheng D. B., Jiang H. Q. (2013). Effect of Tengfu Jiangya Capsules on rat with hypertension. *Chinese Traditional Patent Medicine*.

[9] Hood L. E., Omenn G. S., Moritz R. L. (2012). New and improved proteomics technologies for understanding complex biological systems: addressing a grand challenge in the life sciences. *Proteomics*.

[10] Matt P., Fu Z. M., Fu Q., Van Eyk J. E. (2008). Biomarker discovery: proteome fractionation and separation in biological samples. *Physiological Genomics*.

[11] Cao W., Zhou Y., Li Y. (2015). iTRAQ-based proteomic analysis of combination therapy with taurine, epigallocatechin gallate, and genistein on carbon tetrachloride-induced liver fibrosis in rats. *Toxicology Letters*.

[12] Govorukhina N. I., de Vries M., Reijmers T. H., Horvatovich P., van der Zee A. G. J., Bischoff R. (2009). Influence of clotting time on the protein composition of serum samples based on LC-MS data. *Journal of Chromatography B*.

[13] DeSouza L. V., Grigull J., Ghanny S. (2007). Endometrial carcinoma biomarker discovery and verification using differentially tagged clinical samples with multidimensional liquid chromatography and tandem mass spectrometry. *Molecular and Cellular Proteomics*.

[14] Ralhan R., DeSouza L. V., Matta A. (2008). Discovery and verification of head-and-neck cancer biomarkers by differential protein expression analysis using iTRAQ labeling, multidimensional liquid chromatography, and tandem mass spectrometry. *Molecular and Cellular Proteomics*.

[15] Redding K. M., Chen B. L., Singh A. (2010). Transgenic mice expressing an intracellular fluorescent fusion of angiotensin II demonstrate renal thrombotic microangiopathy and elevated blood pressure. *American Journal of Physiology—Heart and Circulatory Physiology*.

[16] Xu D.-D., Deng D.-F., Li X. (2014). Discovery and identification of serum potential biomarkers for pulmonary tuberculosis using iTRAQ-coupled two-dimensional LC-MS/MS. *Proteomics*.

[17] Kato H., Enjyoji K.-I., Miyata T., Hayashi I., Ohishi S., Iwanaga S. (1985). Demonstration of arginyl-bradykinin moiety in rat HMW kininogen: direct evidence for liberation of bradykinin by rat glandular kallikreins. *Biochemical and Biophysical Research Communications*.

[18] Scharfstein J., Schmitz V., Svensjö E., Granato A., Monteiro A. C. (2007). Kininogens coordinate adaptive immunity through the proteolytic release of bradykinin, an endogenous danger signal driving dendritic cell maturation. *Scandinavian Journal of Immunology*.

[19] Schneider E. G., Strandhoy J. W., Willis L. R., Knox F. G. (1973). Relationship between proximal sodium reabsorption and excretion of calcium, magnesium and phosphate. *Kidney International*.

[20] Katori M., Majima M. (2003). The renal kallikrein-kinin system: its role as a safety valve for excess sodium intake, and its attenuation as a possible etiologic factor in salt-sensitive hypertension. *Critical Reviews in Clinical Laboratory Sciences*.

[21] Remotti F., Fetsch J. F., Miettinen M. (2001). Keratin 1 expression in endothelia and mesenchymal tumors: an immunohistochemical analysis of normal and neoplastic tissues. *Human Pathology*.

[22] Hasan A. A. K., Zisman T., Schmaier A. H. (1998). Identification of cytokeratin 1 as a binding protein and presentation receptor for kininogens on endothelial cells. *Proceedings of the National Academy of Sciences of the United States of America*.

[23] Jerke U., Rolle S., Purfürst B., Luft F. C., Nauseef W. M., Kettritz R. (2013). *β*2 integrin-mediated cell-cell contact transfers active myeloperoxidase from neutrophils to endothelial cells. *Journal of Biological Chemistry*.

[24] Yang J. J., Preston G. A., Pendergraft W. F. (2001). Internalization of proteinase 3 is concomitant with endothelial cell apoptosis and internalization of myeloperoxidase with generation of intracellular oxidants. *American Journal of Pathology*.

[25] Xiao H., Heeringa P., Hu P. (2002). Antineutrophil cytoplasmic autoantibodies specific for myeloperoxidase cause glomerulonephritis and vasculitis in mice. *Journal of Clinical Investigation*.

[26] Walker A. E., Seibert S. M., Donato A. J., Pierce G. L., Seals D. R. (2010). Vascular endothelial function is related to white blood cell count and myeloperoxidase among healthy middle-aged and older adults. *Hypertension*.

[27] Astern J. M., Pendergraft W. F., Falk R. J. (2007). Myeloperoxidase interacts with endothelial cell-surface cytokeratin 1 and modulates bradykinin production by the plasma kallikrein-kinin system. *The American Journal of Pathology*.

[28] Soprano D. R., Soprano K. J., Goodman D. S. (1986). Retinol-binding protein messenger RNA levels in the liver and in extrahepatic tissues of the rat. *Journal of Lipid Research*.

[29] Solini A., Santini E., Madec S., Rossi C., Muscelli E. (2009). Retinol-binding protein-4 in women with untreated essential hypertension. *American Journal of Hypertension*.

[30] Farjo K. M., Farjo R. A., Halsey S., Moiseyev G., Ma J.-X. (2012). Retinol-binding protein 4 induces inflammation in human endothelial cells by an NADPH oxidase- and nuclear factor kappa B-dependent and retinol-independent mechanism. *Molecular and Cellular Biology*.

[31] Sjöholm K., Palming J., Olofsson L. E. (2005). A microarray search for genes predominantly expressed in human omental adipocytes: adipose tissue as a major production site of serum amyloid A. *Journal of Clinical Endocrinology and Metabolism*.

[32] Das U. N. (2005). Is angiotensin-II an endogenous pro-inflammatory molecule?. *Medical Science Monitor*.

[33] Qi Y., Rathinasabapathy A., Huo T. (2015). Dysfunctional adipose stem cell is linked to obesity, elevated inflammatory cytokines and resistant hypertension. *Journal of Hypertension*.

