# Supplementary material for: Identification of Tengfu Jiangya Tablet Target Biomarkers with Quantitative Proteomic Technique
Source: Evid Based Complement Alternat Med. 2017 Mar 20;2017:7594805. doi: 10.1155/2017/7594805 (PMC5376940; doi:10.1155/2017/7594805)
Supplement: Supplementary file 1 — The comparison of blood pressure of EH patients (n=47) before and after TJT treatment. Both of the SBP and DBP were reduced significantly (P<0.01) after TJT treatment. [file 7594805.f1.pdf]

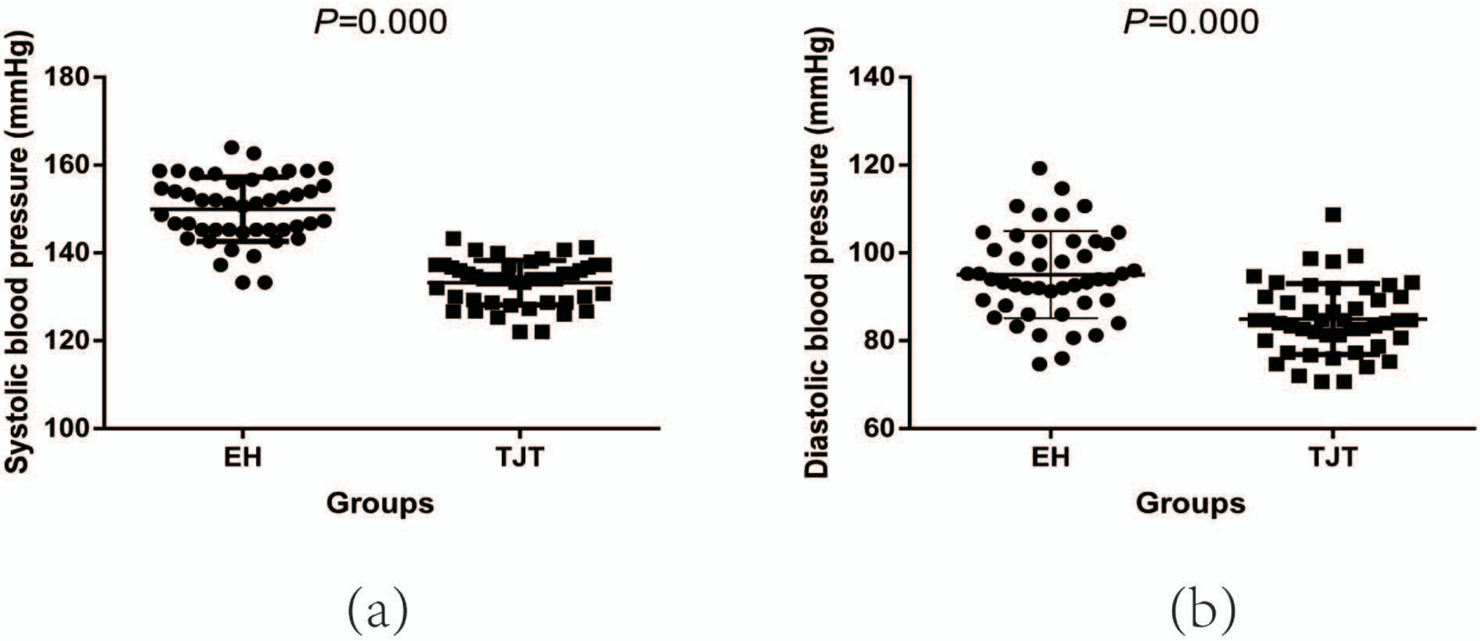

Supplementary Figure 1

Supplementary table 1. The clinical parameters of EH patients between discovery section (n=30) and verification section (n=30).

Clinical parameters	Discovery section (n=30)	Verification section (n=30)	*P*-value
Age(years)	57.1±9.9	59.9±9.2	0.946[a]
Sex(♂/♀)	16/14	14/16	0.606[b]
BMI	25.5±3.0	23.7±2.3	0.126[a]
Smoker	14/30	12/30	0.602
Blood pressure of EH			
Systolic	150.3±8.3	149.3±7.1	0.618[a]
Diastolic	95.0±11.3	95.0±8.3	1.000[a]
Blood pressure of TJT			
Systolic	133.1±5.4	132.2±5.1	0.512[a]
Diastolic	84.6±9.2	84.9±6.5	0.889[a]

Data were expressed as Mean±SD; a) *P*- value between two experiment sections with independent samples T-test; b) Chi-square Test, $P \leqslant 0.05$ was considered to be statistically significant. In additional, the blood pressures between EH and TJT groups were also statistically analyzed by Spss16.0 with Paired-sample T-test, both of the systolic and diastolic blood pressure were significantly different before and after TJT treatment (P=0.000).

*A total of 47 EH individuals were involved in our study. Firstly, 30 patients were selected in the discovery section randomly. Secondly, the remaining 17 patients and 13 patients involved in the first section were jointly analyzed in verification section.

*BMI=Weight (Kg)/Height^2(m^2)

supplementary table2.A list of identified and quantified proteins by iTRAQ proteomics

Hits	Accession	Desription	Mass	Seq	Score	Cov	SameSets	Spectrum	Unique Spe	Peptide	Unique Pep	A-VS-B@R	A-VS-B@R
1	tr|B0YJC6|B0YJC6_HUMAN	Vitamin K-dependent protein Z variant 1 OS=Hom	53450	MAGCVPL	31	2.9	1	1	1	1	1	---	---
2	tr|Q7Z7Q0|Q7Z7Q0_HUMAN	APOB protein OS=Homo sapiens GN=APOB PE=	113702	MDPPRPA	5835	45.5	-	588	1	39	1	---	---
3	sp|P35908|K22E_HUMAN	Keratin, type II cytoskeletal 2 epidermal OS=Hom	76630	MSCQISCK	275	15.5	-	23	13	10	8	0.608	0.607
4	tr|B4DNG0|B4DNG0_HUMAN	Olfactomedin-like protein 3 OS=Homo sapiens GN	44167	MLPLLEV	40	5.8	1	4	4	2	2	---	---
5	sp|P23142|FBLN1_HUMAN	Fibulin-1 OS=Homo sapiens GN=FBLN1 PE=1 S	86744	MERAAPS	851	28.3	-	87	60	17	8	1.115	1.048
6	tr|L0R4T3|L0R4T3_HUMAN	Histone H2B OS=Homo sapiens GN=ABCF2 PE=	8880	MSIMNSF	32	13.4	20	4	4	1	1	---	---
7	tr|C9JF17|C9JF17_HUMAN	Apolipoprotein D (Fragment) OS=Homo sapiens G	28800	MFKQLSC	857	28.8	1	84	84	6	6	0.834	0.781
8	sp|P18428|LBP_HUMAN	Lipopolysaccharide-binding protein OS=Homo sap	60518	MGALARA	2335	20.2	-	145	145	9	9	0.794	0.784
9	tr|B2RMS9|B2RMS9_HUMAN	Inter-alpha (Globulin) inhibitor H4 (Plasma Kallik	116303	MKPPRPV	3903	25.2	1	263	263	21	21	1.114	1.097
10	sp|P03950|ANGI_HUMAN	Angiogenin OS=Homo sapiens GN=ANG PE=1 S	19315	MVMGLG	40	26.5	-	9	9	4	4	0.78	0.697
11	tr|Q0PW96|Q0PW96_HUMAN	Intersectin 1 short form variant 7 OS=Homo sapier	157455	MAQFPTP	25	0.7	10	1	1	1	1	---	---
12	tr|L0R8K6|L0R8K6_HUMAN	Alternative protein TRIM25 OS=Homo sapiens G	9186	MWSLEVI	21	13.6	-	3	3	1	1	---	---
13	tr|B3KRN4|B3KRN4_HUMAN	cDNA FLJ34625 fis, clone KIDNE2015244, highl	57113	MGWAAR	45	5.6	2	3	3	2	2	0.831	0.903
14	sp|P01608|KV116_HUMAN	Ig kappa chain V-I region Roy OS=Homo sapiens	14018	DIQMTQS	137	24.1	-	11	6	2	1	---	---
15	tr|B7Z7R8|B7Z7R8_HUMAN	cDNA FLJ55622, highly similar to Multimerin-1 C	133624	MQHKIVT	301	10.9	2	24	24	10	10	1.033	0.938
16	sp|P00739|HPTR_HUMAN	Haptoglobin-related protein OS=Homo sapiens GN	48340	MSDLGAV	677	36.2	-	72	22	11	4	0.676	0.704
17	tr|B4DEB9|B4DEB9_HUMAN	cDNA FLJ61099, highly similar to ADP-ribosylati	23649	MGNIFAN	29	5.8	7	1	1	1	1	---	---
18	sp|Q13790|APOF_HUMAN	Apolipoprotein F OS=Homo sapiens GN=APOF P	39065	MTGLCGY	45	8.3	-	3	3	2	2	---	---
19	tr|Q6IB71|Q6IB71_HUMAN	Proteasome subunit alpha type OS=Homo sapiens	34405	MSSIGTGY	81	5.5	1	2	2	1	1	1.029	1.096
20	tr|Q6ZNX5|Q6ZNX5_HUMAN	CDNA FLJ26936 fis, clone RCT06808 OS=Homo	18632	MAWALLI	41	4.8	-	2	2	1	1	---	---
21	tr|Q5CZ94|Q5CZ94_HUMAN	Putative uncharacterized protein DKFZp781M038	29340	MAWTPLF	708	30.8	-	75	2	6	1	---	---
22	tr|A0A087WTX5|A0A087WTX	Ig kappa chain C region OS=Homo sapiens GN=IG	30209	MDMRVPA	1394	44.1	-	155	1	7	1	---	---
23	tr|A2MYC8|A2MYC8_HUMA	V5-2 protein (Fragment) OS=Homo sapiens GN=V	12669	QPVLTQP	50	28.8	1	6	6	3	3	---	---
24	tr|G3V2W1|G3V2W1_HUMA	Protein Z-dependent protease inhibitor OS=Homo s	65592	MSRSTQE	346	15.1	1	33	3	8	1	1.054	0.935
25	sp|P19652|A1AG2_HUMAN	Alpha-1-acid glycoprotein 2 OS=Homo sapiens G	28132	MALSWVI	61	9.5	-	7	7	2	2	0.897	0.763
26	tr|D6RF35|D6RF35_HUMAN	Vitamin D-binding protein OS=Homo sapiens GN=	67663	MKRVLVL	55	18.5	1	17	17	9	9	1.692	1.092
27	tr|K7ERI9|K7ERI9_HUMAN	Truncated apolipoprotein C-I (Fragment) OS=Hom	11075	MRLFLSL	409	32.5	1	51	51	3	3	0.739	0.71
28	sp|O76076|WISP2_HUMAN	WNT1-inducible-signaling pathway protein 2 OS=	29677	MRGTPKT	35	4	-	2	2	1	1	---	---
29	tr|Q1L857|Q1L857_HUMAN	Ceruloplasmin (Fragment) OS=Homo sapiens PE=	136274	MKILILGI	862	16.7	3	82	82	13	13	1.059	1.013
30	tr|Q9UL89|Q9UL89_HUMAN	Myosin-reactive immunoglobulin heavy chain varia	14841	VQSGAEV	62	10.3	1	1	1	1	1	---	---
31	tr|Q5SRP5|Q5SRP5_HUMAN	Apolipoprotein M OS=Homo sapiens GN=APOM	17751	MAAGSAP	61	17.8	1	11	11	3	3	0.669	0.73
32	tr|B3KRF9|B3KRF9_HUMAN	cDNA FLJ34156 fis, clone FCBBF3013266, highl	35578	MPWPLLL	124	7.2	1	8	8	2	2	0.946	0.878
33	tr|H0Y7V6|H0Y7V6_HUMAN	Pulmonary surfactant-associated protein B (Fragme	46516	XHLLQWI	24	4.3	5	1	1	1	1	---	---
34	tr|Q7Z351|Q7Z351_HUMAN	Putative uncharacterized protein DKFZp686N022	63846	MEFGLTW	3561	29.9	-	309	2	11	1	---	---
35	sp|Q15113|PCOC1_HUMAN	Procollagen C-endopeptidase enhancer 1 OS=Hom	55490	MLPAATA	347	28.1	-	40	40	11	11	1.08	1.092
36	tr|A0A068LKR7|A0A068LKR7	Ig heavy chain variable region (Fragment) OS=Ho	15875	QVQLVES	102	32.8	1	10	3	3	1	1.055	0.94
37	tr|F8VV32|F8VV32_HUMAN	Lysozyme C OS=Homo sapiens GN=LYZ PE=1 S	13895	MKALIVL	163	23.1	1	12	12	2	2	1.512	1.505
38	sp|Q6UY14|ATL4_HUMAN	ADAMTS-like protein 4 OS=Homo sapiens GN=A	123181	MENWTG	64	3.8	1	3	3	3	3	0.878	0.978
39	tr|Q8TAN2|Q8TAN2_HUMAN	Frizzled homolog 9 (Drosophila) OS=Homo sapier	71986	MAVAPLR	31	1.7	1	9	9	1	1	---	---
40	sp|P34096|RNAS4_HUMAN	Ribonuclease 4 OS=Homo sapiens GN=RNASE4	19111	MALQRTH	49	18.4	-	5	5	2	2	1.505	0.764
41	tr|B2R984|B2R984_HUMAN	cDNA, FLJ94268, highly similar to Homo sapiens	41045	MSETAPA	48	8.2	5	2	2	2	2	---	---
42	sp|Q86UD1|OAF_HUMAN	Out at first protein homolog OS=Homo sapiens GN	35194	MRLPGVP	90	3.3	-	11	11	1	1	1.119	1.026
43	sp|P02741|CRP_HUMAN	C-reactive protein OS=Homo sapiens GN=CRP PE	29757	MEKLLCF	309	23.7	-	59	59	6	6	1.125	1.098
44	sp|P80108|PHLD_HUMAN	Phosphatidylinositol-glycan-specific phospholipase	103248	MSAFRLW	206	11.1	-	18	18	8	8	1.146	1.04
45	tr|A0A075B7C5|A0A075B7C5_	C-C motif chemokine 5 (Fragment) OS=Homo sap	19150	XAALAVII	23	5.3	2	2	2	1	1	---	---
46	tr|F1C4A7|F1C4A7_HUMAN	Monocyte differentiation antigen CD14 OS=Homo	43702	MERASCL	185	19.2	2	18	18	6	6	0.594	0.483
47	tr|S6C4Q9|S6C4Q9_HUMAN	IgG L chain OS=Homo sapiens PE=2 SV=1	26779	MAWALLI	1176	24	-	134	2	5	1	---	---
48	tr|A5PLK9|A5PLK9_HUMAN	Metalloendopeptidase OS=Homo sapiens GN=BM	131159	MPGVARL	78	1.9	2	4	4	2	2	0.963	1.105
49	tr|B3KRV7|B3KRV7_HUMAN	cDNA FLJ34967 fis, clone NTONG2004690, high	63225	MESKALL	89	10.8	4	8	8	5	5	1.056	1.014
50	sp|Q14520|HABP2_HUMAN	Hyaluronan-binding protein 2 OS=Homo sapiens	76908	MFARMSD	160	10.2	-	34	34	6	6	1.19	1.1
51	tr|Q5VY30|Q5VY30_HUMAN	Retinol binding protein 4, plasma, isoform CRA_b	26952	MNYSKIP	63	15.1	1	7	7	3	3	2.028	1.684
52	tr|Q9NSD0|Q9NSD0_HUMAN	Protein S OS=Homo sapiens PE=2 SV=1	89278	MSKQQAS	489	16.3	2	60	60	12	12	1.181	1.13
53	sp|P20160|CAP7_HUMAN	Azurocidin OS=Homo sapiens GN=AZU1 PE=1 S	27933	MTRLTVL	123	8	-	8	8	2	2	1.254	1.791
54	tr|A0N8J8|A0N8J8_HUMAN	V lambda (Fragment) OS=Homo sapiens PE=4 SV	13760	MAWTPLF	57	21.6	2	11	2	3	1	---	---
55	tr|Q6PIK1|Q6PIK1_HUMAN	IGL@ protein OS=Homo sapiens GN=IGL@ PE=	29690	MAWALLI	816	34	-	95	4	7	1	---	---
56	tr|B4E1T8|B4E1T8_HUMAN	cDNA FLJ51452, highly similar to Lysyl oxidase	51776	MALARTS	28	4.6	1	1	1	1	1	---	---
57	sp|P35579|MYH9_HUMAN	Myosin-9 OS=Homo sapiens GN=MYH9 PE=1 S	290921	MAQQAAI	34	1.4	-	4	2	3	2	---	---
58	sp|P02533|K1C14_HUMAN	Keratin, type I cytoskeletal 14 OS=Homo sapiens	59172	MTTCSRQ	50	4.4	1	2	1	2	1	---	---
59	sp|Q15166|PON3_HUMAN	Serum paraoxonase/lactonase 3 OS=Homo sapiens	46199	MGKLVAI	183	14.7	-	39	39	5	5	1.026	0.971
60	tr|B3KML9|B3KML9_HUMAN	cDNA FLJ11352 fis, clone HEMBA1000020, high	49175	MRENVHI	52	10.1	16	4	4	3	3	---	---
61	tr|H0Y512|H0Y512_HUMAN	Adipocyte plasma membrane-associated protein (F	50834	XVVTDDD	169	8.6	1	14	14	3	3	0.922	0.865
62	tr|U3KPS2|U3KPS2_HUMAN	Myeloblastin OS=Homo sapiens GN=PRTN3 PE=	25269	MASLQMF	42	5.6	2	2	2	1	1	---	---
63	tr|B2RBW9|B2RBW9_HUMA	cDNA, FLJ95746, highly similar to Homo sapiens	41072	MTSSLLL	90	5.1	1	13	13	2	2	0.832	0.786
64	tr|A0A068LL62|A0A068LL62_	Ig heavy chain variable region (Fragment) OS=Ho	14698	QVQLQQV	40	12.7	-	6	2	2	1	---	---
65	tr|H0YK48|H0YK48_HUMAN	Tropomyosin alpha-1 chain OS=Homo sapiens GN	36225	MAGSSSL	52	9.7	5	3	1	2	1	---	---
66	tr|B7Z4X6|B7Z4X6_HUMAN	cDNA FLJ51012, highly similar to Plasminogen ac	43747	MLQLTTG	40	14.9	2	12	12	5	5	1.167	1.24
67	sp|P01009|A1AT_HUMAN	Alpha-1-antitrypsin OS=Homo sapiens GN=SERP	57525	MPSSVSW	610	29.2	-	47	47	11	11	0.877	0.901
68	sp|P08294|SODE_HUMAN	Extracellular superoxide dismutase [Cu-Zn] OS=H	28116	MLALLCS	390	25.8	-	19	19	4	4	1.044	1.053
69	tr|Q6U2E7|Q6U2E7_HUMAN	C4B1 (Fragment) OS=Homo sapiens GN=C4B PE	6651	VSAGSPH	291	80.3	-	45	1	3	1	---	---
70	tr|A2RRB2|A2RRB2_HUMAN	ZNF608 protein OS=Homo sapiens GN=ZNF608	57576	MSVNIST	25	2	3	2	2	1	1	---	---
71	sp|P04180|LCAT_HUMAN	Phosphatidylcholine-sterol acyltransferase OS=Ho	53843	MGPPGSP	341	14.8	-	35	35	6	6	0.988	1.027
72	tr|J3KRP0|J3KRP0_HUMAN	Beta-Ala-His dipeptidase OS=Homo sapiens GN=	59295	MDPKLGR	122	15.5	2	12	12	8	8	1.078	1.127
73	tr|J3KQ66|J3KQ66_HUMAN	Reelin OS=Homo sapiens GN=RELN PE=4 SV=1	428795	MERSGW	39	0.7	1	4	4	3	3	---	---
74	tr|Q9HCC1|Q9HCC1_HUMAN	Single chain Fv (Fragment) OS=Homo sapiens PE=	13566	EVQLVES	64	36.6	-	9	2	3	1	---	---
75	tr|B7Z1F8|B7Z1F8_HUMAN	cDNA FLJ53025, highly similar to Complement C	36015	MCPEDSL	8244	56.7	-	678	64	14	2	1.052	1.046
76	sp|P20851|C4BPB_HUMAN	C4b-binding protein beta chain OS=Homo sapiens	35088	MFFWCAC	477	21.8	-	43	43	5	5	0.841	0.783
77	tr|Q9UL70|Q9UL70_HUMAN	Myosin-reactive immunoglobulin light chain variab	13869	DIQMTQS	123	23.1	-	6	1	2	1	---	---
78	sp|Q02985|FHR3_HUMAN	Complement factor H-related protein 3 OS=Homo	44580	MLLLINVI	81	14.5	-	11	10	5	4	1.233	1.352
79	tr|A8K6C1|A8K6C1_HUMAN	cDNA FLJ76868, highly similar to Homo sapiens	63358	MLAATVL	447	10.8	1	41	41	6	6	0.721	0.829
80	tr|B3KS79|B3KS79_HUMAN	cDNA FLJ35730 fis, clone TESTI2003131, highly	59285	MKIHYSR	136	9.8	1	11	11	4	4	1.034	0.889
81	sp|P01137|TGFB1_HUMAN	Transforming growth factor beta-1 OS=Homo sapi	50777	MPPSGLR	52	8.7	-	7	7	4	4	0.822	0.9
82	tr|B5MCZ9|B5MCZ9_HUMAN	Glutaminyl-peptide cyclotransferase (Fragment) O	35244	MAGGRH	31	4.9	1	2	2	1	1	---	---
83	tr|D6REX5|D6REX5_HUMAN	Selenoprotein P (Fragment) OS=Homo sapiens GN	42698	MWRSLGI	165	12.6	1	9	9	4	4	0.731	0.894
84	tr|B2R7Z6|B2R7Z6_HUMAN	cDNA, FLJ93674 OS=Homo sapiens PE=2 SV=1	62079	MAGPWT	99	10.7	1	7	7	5	5	0.703	0.649
85	tr|B2R6W1|B2R6W1_HUMAN	cDNA, FLJ93143, highly similar to Homo sapiens	111585	MKVISLF	635	25.9	-	91	4	19	2	1.218	1.098
86	tr|A8K061|A8K061_HUMAN	cDNA FLJ77880, highly similar to Homo sapiens	65697	MFTIKLLL	39	8.7	1	4	4	4	4	---	---
87	tr|B2RA39|B2RA39_HUMAN	cDNA, FLJ94686, highly similar to Homo sapiens	77962	MLLLFSVI	324	30.4	1	63	56	15	14	1.117	1.128
88	sp|P01707|LV204_HUMAN	Ig lambda chain V-II region TRO OS=Homo sapie	13550	QSALTQP	34	14.4	-	3	2	2	1	---	---
89	tr|Q9UL82|Q9UL82_HUMAN	Myosin-reactive immunoglobulin light chain variab	13377	SYELTQPS	31	16.8	-	7	7	2	2	---	---
90	tr|A8K008|A8K008_HUMAN	cDNA FLJ78387 OS=Homo sapiens PE=1 SV=1	63504	MEFGLSW	3565	31.8	-	310	1	12	1	---	---
91	sp|P48061|SDF1_HUMAN	Stromal cell-derived factor 1 OS=Homo sapiens G	14290	MNAKVV	23	17.2	-	2	2	2	2	---	---
92	sp|P27918|PROP_HUMAN	Properdin OS=Homo sapiens GN=CFP PE=1 SV=	59531	MITEGAQ	207	14.7	-	29	29	6	6	1.065	1.004
93	tr|B4DF70|B4DF70_HUMAN	cDNA FLJ60461, highly similar to Peroxiredoxin-	23555	MASGNAF	47	9.8	1	2	2	2	2	---	---
94	sp|P05160|F13B_HUMAN	Coagulation factor XIII B chain OS=Homo sapiens	91735	MRLKNLT	41	4.7	-	4	4	3	3	---	---
95	tr|A2NH53|A2NH53_HUMAN	Immunogobulin kappa, VJ region (Fragment) OS=	13215	THGEIVM	34	18.8	2	1	1	1	1	---	---
96	tr|A2NKM6|A2NKM6_HUMA	NANUC-1 heavy chain (Fragment) OS=Homo sap	16555	LEQSGGG	99	23.1	-	9	1	3	1	---	---
97	sp|P02776|PLF4_HUMAN	Platelet factor 4 OS=Homo sapiens GN=PF4 PE=1	13861	MSSAAGF	776	35.6	-	107	62	4	2	0.947	1.005
98	tr|B0YIW1|B0YIW1_HUMAN	Apolipoprotein A-V variant 3 OS=Homo sapiens C	45822	MAAVLTV	228	19.8	2	22	22	7	7	1.378	1.211
99	tr|E7EUJ1|E7EUJ1_HUMAN	Hepatic triacylglycerol lipase OS=Homo sapiens G	63567	MDTSPLC	44	3.4	3	1	1	1	1	---	---
100	sp|P06727|APOA4_HUMAN	Apolipoprotein A-IV OS=Homo sapiens GN=APO	54193	MFLKAVV	3978	58.1	-	422	422	24	24	1.157	1.174
101	tr|Q5UGI6|Q5UGI6_HUMAN	Serine/cysteine proteinase inhibitor clade G membe	44679	GLQADPA	122	14.4	2	8	8	4	4	0.963	0.848
102	tr|H0UI83|H0UI83_HUMAN	Proteasome subunit alpha type OS=Homo sapiens	21075	MSYDRAI	36	5.4	2	1	1	1	1	---	---
103	sp|P07237|PDIA1_HUMAN	Protein disulfide-isomerase OS=Homo sapiens GN	72082	MLRRALL	161	18.3	-	18	18	9	9	1.068	1.014
104	tr|H0YJV4|H0YJV4_HUMAN	Solute carrier family 35 member F4 (Fragment) OS	7109	XYLLALK	23	15.2	-	1	1	1	1	---	---
105	tr|H6VRF8|H6VRF8_HUMAN	Keratin 1 OS=Homo sapiens GN=KRT1 PE=3 SV	75006	MSRQFTS	670	24.8	4	46	36	13	11	0.504	0.562

106	sp|P02671|FIBA_HUMAN	Fibrinogen alpha chain OS=Homo sapiens GN=FG	109041	MFSMRIV	373	16.9	-		42	42	13	13	1.008	1.028
107	tr|B7Z565|B7Z565_HUMAN	cDNA FLJ54739, highly similar to Alpha-actinin-	111056	MLLLEVIS	38	2.4		2	4	4	2	2	---	---
108	tr|A0A024RDT5|A0A024RDT5	Periostin, osteoblast specific factor, isoform CRA_	106758	MIPFLPMF	55	2.1		3	1	1	1	1	---	---
109	tr|A0A024R755|A0A024R755_	Calumenin, isoform CRA_a OS=Homo sapiens GN	45743	MDLRQFL	47	5.7		3	3	3	2	2	---	---
110	sp|P10909|CLUS_HUMAN	Clusterin OS=Homo sapiens GN=CLU PE=1 SV=	62157	MMKTLLL	3979	33.9	-		340	340	15	15	0.957	1
111	tr|A2J1N0|A2J1N0_HUMAN	Rheumatoid factor RF-IP14 (Fragment) OS=Homo	11519	QLVESGG	99	30.2	-		15	4	3	1	---	---
112	sp|P10645|CMGA_HUMAN	Chromogranin-A OS=Homo sapiens GN=CHGA P	60563	MRSAAVL	118	6.1	-		7	7	3	3	1.061	1.172
113	tr|Q9NPP6|Q9NPP6_HUMAN	Immunoglobulin heavy chain variant (Fragment) O	50481	PGKGLEW	578	22.1	-		72	2	8	1	---	---
114	sp|P22692|IBP4_HUMAN	Insulin-like growth factor-binding protein 4 OS=H	33068	MLPLCLV.	34	5.4	-		3	3	1	1	1.051	0.927
115	sp|P01619|KV301_HUMAN	Ig kappa chain V-III region B6 OS=Homo sapiens	12959	ZIVLTZSP	179	16.7	-		8	8	3	3	1.039	0.874
116	sp|Q16610|ECM1_HUMAN	Extracellular matrix protein 1 OS=Homo sapiens G	67099	MGTTARA	464	27	-		54	54	13	13	0.93	0.848
117	sp|P06331|HV209_HUMAN	Ig heavy chain V-II region ARH-77 OS=Homo sap	18519	MKHLWFI	58	17.1	-		6	4	2	1	1.099	1.024
118	sp|P14543|NID1_HUMAN	Nidogen-1 OS=Homo sapiens GN=NID1 PE=1 SV	148268	MLASSSRI	80	5.4	-		10	10	6	6	1.16	0.906
119	sp|Q96PD5|PGRP2_HUMAN	N-acetylmuramoyl-L-alanine amidase OS=Homo s	66703	MAQGVLV	186	8.7	-		13	13	4	4	0.813	0.694
120	tr|Q6FGL0|Q6FGL0_HUMAN	Galectin (Fragment) OS=Homo sapiens GN=LGA	28940	MADNFSL	38	4.4		3	1	1	1	1	---	---
121	tr|Q1HP67|Q1HP67_HUMAN	Lipoprotein, Lp(A) OS=Homo sapiens GN=LPA P	240494	MEHKEVV	211	6.1		1	17	17	10	10	0.451	0.452
122	tr|B4DI57|B4DI57_HUMAN	cDNA FLJ54111, highly similar to Serotransferrin	80545	MNQLRGK	134	10.2		4	11	11	6	6	1.114	0.93
123	tr|C9JC84|C9JC84_HUMAN	Fibrinogen gamma chain OS=Homo sapiens GN=F	63883	MSWSLHP	299	25.8		2	32	32	11	11	0.977	1.13
124	sp|P40197|GPV_HUMAN	Platelet glycoprotein V OS=Homo sapiens GN=GP	65693	MLRGTLL	67	13.2	-		10	10	6	6	0.68	0.474
125	tr|B2R773|B2R773_HUMAN	cDNA, FLJ93312, highly similar to Homo sapiens	30174	MLLLGAV	151	16		1	12	12	3	3	1.067	1.1
126	tr|B4DUV1|B4DUV1_HUMAN	Fibulin-1 OS=Homo sapiens PE=2 SV=1	79879	MATHQKI	284	25.9	-		42	15	13	4	---	---
127	tr|E7EV88|E7EV88_HUMAN	A disintegrin and metalloproteinase with thrombos	52259	MASDGAA	75	8.8		1	6	6	4	4	1.187	0.911
128	sp|P08697|A2AP_HUMAN	Alpha-2-antiplasmin OS=Homo sapiens GN=SERI	60957	MALLWGI	421	17.7	-		62	62	8	8	0.94	0.815
129	tr|Q6MZM7|Q6MZM7_HUMA	Putative uncharacterized protein DKFZp686O1216	262413	ARAGCAA	9056	32.7	-		750	1	54	1	---	---
130	tr|K7ER74|K7ER74_HUMAN	Protein APOC4-APOC2 OS=Homo sapiens GN=A	23306	MSLLRNR	455	28.1		1	66	66	4	4	0.977	0.997
131	tr|A2J1N5|A2J1N5_HUMAN	Rheumatoid factor RF-ET6 (Fragment) OS=Homo	12382	VESGGGV	49	23.4	-		6	1	2	1	---	---
132	tr|A0A0A0MT96|A0A0A0MT9	Protein IGKJ3 (Fragment) OS=Homo sapiens GN=	2332	XFTFGPGT	28	69.2	-		1	1	1	1	---	---
133	tr|S6AWD3|S6AWD3_HUMAI	IgG L chain OS=Homo sapiens PE=2 SV=1	22895	MRLPAQL	907	23.5	-		130	1	4	1	---	---
134	sp|P07357|CO8A_HUMAN	Complement component C8 alpha chain OS=Homo	76870	MFAVVFF	244	15.2	-		28	28	8	8	1.216	1.033
135	tr|D3DQX7|D3DQX7_HUMAI	Serum amyloid A protein OS=Homo sapiens GN=S	15436	MKLLTGL	105	20.5	-		10	9	3	2	1.459	1.048
136	tr|B2R6M6|B2R6M6_HUMAN	cDNA, FLJ93024, highly similar to Homo sapiens	60522	MLKALFL	224	9.7		6	22	22	4	4	1.206	1.336
137	tr|Q96K68|Q96K68_HUMAN	cDNA FLJ14473 fis, clone MAMMA1001080, hig	59670	MELGLRW	1125	28.3	-		121	58	11	4	1.124	1.135
138	tr|Q6ZRG8|Q6ZRG8_HUMAN	cDNA FLJ46365 fis, clone TESTI4051054 OS=H	23673	MGPGIVL	30	3.2		1	5	5	1	1	---	---
139	sp|P67936|TPM4_HUMAN	Tropomyosin alpha-4 chain OS=Homo sapiens GN	36528	MAGLNSL	106	21.8	-		11	6	5	3	1.325	1.22
140	tr|A6YID2|A6YID2_HUMAN	Fibronectin splice variant A (Fragment) OS=Homo	74428	SQMQVTD	2728	39.2		3	214	1	16	1	---	---
141	sp|P27169|PON1_HUMAN	Serum paraoxonase/arylesterase 1 OS=Homo sapie	46266	MAKLIAL	613	23.1	-		81	81	8	8	1.039	0.917
142	tr|A0A024RCD0|A0A024RCD	Proprotein convertase subtilisin/kexin type 6, isofo	109973	MDPQVKV	25	2.1		7	2	2	2	2	---	---
143	tr|B4DN21|B4DN21_HUMAN	cDNA FLJ53365, highly similar to Homo sapiens	38489	MIFEEHGI	2830	43.1		1	173	4	11	1	0.911	0.845
144	sp|P02763|A1AG1_HUMAN	Alpha-1-acid glycoprotein 1 OS=Homo sapiens GN	28288	MALSWVI	54	7.5	-		5	5	1	1	0.797	0.895
145	tr|E1U340|E1U340_HUMAN	ZNF511/PRAP1 fusion protein OS=Homo sapiens	41096	MQVADVI	30	5.5		2	4	4	2	2	---	---
146	tr|D6RAR4|D6RAR4_HUMAN	Hepatocyte growth factor activator OS=Homo sapi	78528	MGRWAW	45	1.7		1	4	4	1	1	---	---
147	tr|A0A087WYJ9|A0A087WYJ	Ig mu chain C region OS=Homo sapiens GN=IGH	74671	MSVSFLIF	7728	35.4	-		611	4	21	2	---	---
148	tr|B2RBZ5|B2RBZ5_HUMAN	cDNA, FLJ95778, highly similar to Homo sapiens	60491	MKVVPSL	328	16.4	-		31	1	8	1	---	---
149	tr|D3DQH5|D3DQH5_HUMAI	Coiled-coil domain containing 69, isoform CRA_a	41482	MGCRHSR	29	4.7	-		2	2	2	2	---	---
150	tr|B7Z549|B7Z549_HUMAN	cDNA FLJ56821, highly similar to Inter-alpha-tryp	87971	MDGAMG	1649	28.1		1	119	119	15	15	1.098	1.03
151	tr|F5H0G0|F5H0G0_HUMAN	ATP-binding cassette sub-family B member 9 OS=	33165	MRLWKA	33	2.6		3	5	5	1	1	---	---
152	tr|A2IPI4|A2IPI4_HUMAN	HRV Fab 025-VL (Fragment) OS=Homo sapiens I	14086	MAELQMT	67	8.8	-		2	2	1	1	---	---
153	sp|P00734|THRB_HUMAN	Prothrombin OS=Homo sapiens GN=F2 PE=1 SV=	80601	MAHVRGI	5988	58.5	-		555	555	31	31	1.043	1.089
154	sp|Q92954|PRG4_HUMAN	Proteoglycan 4 OS=Homo sapiens GN=PRG4 PE=	202736	MAWKTLI	94	4.5	-		12	12	7	7	0.695	0.696
155	sp|P01861|IGHG4_HUMAN	Ig gamma-4 chain C region OS=Homo sapiens GN	44036	ASTKGPS	603	25.1	-		38	3	6	2	---	---
156	tr|Q8TCZ8|Q8TCZ8_HUMAN	Apolipoprotein E (Fragment) OS=Homo sapiens G	7933	RLGADME	953	54.4	-		49	1	3	1	---	---
157	sp|P01708|LV205_HUMAN	Ig lambda chain V-II region BUR OS=Homo sapie	13495	QSALTQPI	36	10.1	-		1	1	1	1	---	---
158	sp|P07996|TSP1_HUMAN	Thrombospondin-1 OS=Homo sapiens GN=THBS	150327	MGLAWG	1599	25.5	-		210	4	27	1	1.534	0.833
159	sp|Q4G0X9|CCD40_HUMAN	Coiled-coil domain-containing protein 40 OS=Hon	155929	MAEPGGA	21	0.7	-		4	4	1	1	---	---
160	sp|O00187|MASP2_HUMAN	Mannan-binding lectin serine protease 2 OS=Homo	86928	MRLLTLL	241	12.5	-		30	30	9	9	1.078	1.235
161	tr|B1AH77|B1AH77_HUMAN	Ras-related C3 botulinum toxin substrate 2 OS=Ho	21005	MVDSKPV	35	9.5		3	1	1	1	1	---	---
162	tr|B4E1D8|B4E1D8_HUMAN	cDNA FLJ51597, highly similar to C4b-binding pr	71792	MDITLTET	1578	33.4		2	226	226	16	16	1.059	1.036
163	tr|Q4LE64|Q4LE64_HUMAN	NUMA1 variant protein (Fragment) OS=Homo sap	286341	LSGITKMT	24	0.3		1	1	1	1	1	---	---
164	sp|Q86U17|SPA11_HUMAN	Serpin A11 OS=Homo sapiens GN=SERPINA11 I	53519	MGPAWLV	42	3.3	-		1	1	1	1	---	---
165	tr|B7ZLY3|B7ZLY3_HUMAN	LTBP1 protein OS=Homo sapiens GN=LTBP1 PE	175722	MDTKLM	66	3		3	6	6	3	3	0.923	0.852
166	tr|B4DDF8|B4DDF8_HUMAN	cDNA FLJ51786, highly similar to Retinal dehydro	56272	MDASERG	24	2.1		3	1	1	1	1	---	---
167	tr|G3XAM2|G3XAM2_HUMA	Complement factor I light chain OS=Homo sapiens	80796	MKLLHVF	64	5.7		4	5	5	3	3	1.123	1.084
168	sp|P01024|CO3_HUMAN	Complement C3 OS=Homo sapiens GN=C3 PE=1	223249	MGPTSGP	14077	52.2	-		1426	1426	79	79	0.879	0.874
169	tr|B0YIW2|B0YIW2_HUMAN	Apolipoprotein C-III OS=Homo sapiens GN=APO	14994	MGTWGA	1654	29.1		1	130	130	3	3	0.871	0.87
170	tr|A0A075B6J9|A0A075B6J9_	HCG1782423 (Fragment) OS=Homo sapiens GN=	13724	MAWALLI	43	10.8		1	1	1	1	1	---	---
171	sp|P0DJI9|SAA2_HUMAN	Serum amyloid A-2 protein OS=Homo sapiens GN	15401	MKLLTGL	29	20.5	-		2	1	2	1	---	---
172	sp|P01031|CO5_HUMAN	Complement C5 OS=Homo sapiens GN=C5 PE=1	226401	MGLLGIL	1032	21.8	-		167	167	36	36	1.115	1.104
173	tr|C1K3N3|C1K3N3_HUMAN	Insulin-like growth factor binding protein 1 (Fragm	31823	SEVPVAR	94	11.2		3	9	9	3	3	0.994	0.859
174	tr|A0A068LRW6|A0A068LRW	Ig heavy chain variable region (Fragment) OS=Ho	16657	QVTLRES	25	5.5		2	1	1	1	1	---	---
175	tr|A0A024RD39|A0A024RD39	Phospholipase A2, group VII (Platelet-activating fa	59600	MVPPKLH	38	1.8		2	2	2	1	1	---	---
176	tr|A0A087X1J7|A0A087X1J7_	Glutathione peroxidase OS=Homo sapiens GN=GI	30481	MARLLQA	713	34.7		1	101	101	7	7	1.097	1.038
177	sp|P48740|MASP1_HUMAN	Mannan-binding lectin serine protease 1 OS=Homo	92409	MRWLLLY	566	26	-		59	15	14	6	1.306	1.34
178	sp|P04220|MUCB_HUMAN	Ig mu heavy chain disease protein OS=Homo sapie	49019	DSPLEQSC	2388	33.8	-		286	3	12	1	---	---
179	tr|J3KPY9|J3KPY9_HUMAN	Anthrax toxin receptor 2 OS=Homo sapiens GN=A	54148	MRLSFIVF	65	16.3		1	8	7	6	5	0.535	0.454
180	sp|P02760|AMBP_HUMAN	Protein AMBP OS=Homo sapiens GN=AMBP PE	45666	MRSLGAL	138	16.5	-		11	11	4	4	1.126	1.171
181	sp|P58166|INHBE_HUMAN	Inhibin beta E chain OS=Homo sapiens GN=INHE	41712	MRLPDVQ	64	2.3	-		5	5	1	1	0.859	0.921
182	sp|Q07960|RHG01_HUMAN	Rho GTPase-activating protein 1 OS=Homo sapier	59892	MDPLSEL	31	2.3	-		1	1	1	1	---	---
183	tr|H0YJ31|H0YJ31_HUMAN	Fibulin-5 (Fragment) OS=Homo sapiens GN=FBL	19441	XFETGSCS	44	10.2		4	1	1	1	1	---	---
184	tr|B4E0A4|B4E0A4_HUMAN	cDNA FLJ57023, highly similar to Homo sapiens	149266	MRKHRHI	124	4.3		4	12	12	5	5	0.562	0.549
185	sp|P06312|KV401_HUMAN	Ig kappa chain V-IV region (Fragment) OS=Homo	15007	MVLQTQV	177	17.4		1	19	2	2	1	---	---
186	sp|P01591|IGJ_HUMAN	Immunoglobulin J chain OS=Homo sapiens GN=IG	21585	MKNHLLF	69	35.2	-		23	23	5	5	1.166	1.094
187	tr|A0A087WZW8|A0A087WZV	Protein IGKV3-11 OS=Homo sapiens GN=IGKV3	29520	MEAPAQL	1412	49.8	-		164	2	8	1	---	---
188	tr|A0A0A0MRJ7|A0A0A0MRJ	Coagulation factor V OS=Homo sapiens GN=F5 P	292156	MFPGCPR	1409	16.3		1	166	164	33	32	0.961	0.975
189	tr|B4DPQ0|B4DPQ0_HUMAN	Complement C1r subcomponent OS=Homo sapiens	94632	MSVRFRV	429	26.6		4	48	48	16	16	1.163	1.031
190	sp|P0C0S8|H2A1_HUMAN	Histone H2A type 1 OS=Homo sapiens GN=HIST	18646	MSGRGKC	46	14.6		12	2	2	1	1	1.529	1.57
191	sp|P05164|PERM_HUMAN	Myeloperoxidase OS=Homo sapiens GN=MPO PE	91781	MGVPFFSS	129	8.5	-		15	8	6	4	1.748	1.988
192	tr|L8E853|L8E853_HUMAN	von Willebrand factor OS=Homo sapiens GN=VW	341812	MIPARFAC	627	7.5	-		65	65	19	19	0.813	0.665
193	tr|A0A024R035|A0A024R035_	Complement component 9, isoform CRA_a OS=Ho	76205	MSACWSF	1009	28.3		1	111	111	18	18	1.023	1.05
194	tr|A0A087WTK4|A0A087WTK	Protein IGKV1-16 OS=Homo sapiens GN=IGKV1	12029	DIQMTQSI	143	35.1	-		7	2	2	1	0.939	0.974
195	sp|P08311|CATG_HUMAN	Cathepsin G OS=Homo sapiens GN=CTSG PE=1	30682	MQPLLLLI	27	3.1	-		1	1	1	1	---	---
196	sp|Q9H6X2|ANTR1_HUMAN	Anthrax toxin receptor 1 OS=Homo sapiens GN=A	72255	MATAERR	34	5	-		2	1	2	1	---	---
197	sp|P43652|AFAM_HUMAN	Afamin OS=Homo sapiens GN=AFM PE=1 SV=1	86781	MKLLKLT	210	14.5	-		33	33	9	9	1.168	1.038
198	sp|P04004|VTNC_HUMAN	Vitronectin OS=Homo sapiens GN=VTN PE=1 SV	61458	MAPLRPLI	8430	29.7	-		729	725	13	11	1.096	1.11
199	sp|P00738|HPT_HUMAN	Haptoglobin OS=Homo sapiens GN=HP PE=1 SV	56812	MSALGAV	497	27.6	-		58	8	10	3	1.048	0.926
200	sp|Q9Y490|TLN1_HUMAN	Talin-1 OS=Homo sapiens GN=TLN1 PE=1 SV=3	321351	MVALSLK	66	2.3	-		4	4	4	4	1.259	0.849
201	tr|A2NB43|A2NB43_HUMAN	Cold agglutinin FS-1 H-chain (Fragment) OS=Hon	14984	QVQLQQV	21	7.6	-		1	1	1	1	---	---
202	tr|Q75N88|Q75N88_HUMAN	Fibrillin 1 OS=Homo sapiens GN=FBN1 PE=4 SV	173316	MRRGRLL	30	1.3		1	2	1	2	1	---	---
203	sp|P01833|PIGR_HUMAN	Polymeric immunoglobulin receptor OS=Homo sap	97510	MLLFVLT	37	6.2	-		7	7	5	5	---	---
204	sp|P30043|BLVRB_HUMAN	Flavin reductase (NADPH) OS=Homo sapiens GN	25262	MAVKKIA	33	4.9	-		1	1	1	1	---	---
205	tr|Q9UMV1|Q9UMV1_HUMA	Complement C4B1a (Fragment) OS=Homo sapiens	5960	AAIIESPG	102	23.9		1	14	14	1	1	2.168	1.75
206	tr|A0A075B6K3|A0A075B6K3	Protein IGLV2-11 (Fragment) OS=Homo sapiens	14328	MAWALLI	93	13.4		1	14	13	2	1	---	---
207	tr|A8K2T4|A8K2T4_HUMAN	cDNA FLJ78207, highly similar to Human comple	111446	MKVISLFI	608	25.9		1	88	1	18	1	---	---
208	tr|A0A024R7F1|A0A024R7F1_	Protein kinase C substrate 80K-H, isoform CRA_a	70571	MLLPLLLI	36	1.3		5	3	3	1	1	---	---
209	sp|Q15485|FCN2_HUMAN	Ficolin-2 OS=Homo sapiens GN=FCN2 PE=1 SV	39607	MELDRAV	308	14.7	-		38	38	5	5	2.859	2.981
210	sp|P03951|FA11_HUMAN	Coagulation factor XI OS=Homo sapiens GN=F11	84893	MIFLYQV	40	3.2	-		3	3	2	2	---	---
211	tr|Q6UXM4|Q6UXM4_HUMA	Ficolin (Collagen/fibrinogen domain containing) 3	34547	MDLLWIL	731	26.4		1	80	80	8	8	1.18	1.258
212	tr|B0I1S0|B0I1S0_HUMAN	DYNC2H1 variant protein OS=Homo sapiens PE=	588296	MANGTAI	28	0.2		1	7	7	1	1	---	---
213	tr|D6RD58|D6RD58_HUMAN	Leukocyte cell-derived chemotaxin-2 OS=Homo sa	12319	MIVGQEK	33	11.4		1	3	3	1	1	---	---
214	sp|P02647|APOA1_HUMAN	Apolipoprotein A-I OS=Homo sapiens GN=APOA	37756	MKAAVLT	9312	77.5	-		956	956	26	26	1.023	1.057

215	tr|Q96JD0|Q96JD0_HUMAN	Amyloid lambda 6 light chain variable region SAR	13922	NFMLTQP	149	20.7	-	39	1	2	1	---	---
216	tr|G3XAK1|G3XAK1_HUMAN	Hepatocyte growth factor-like protein alpha chain (	91508	MGLWWV	56	4.1	2	3	3	2	2	---	---
217	tr|E7EUT5|E7EUT5_HUMAN	Glyceraldehyde-3-phosphate dehydrogenase OS=H	34412	MEEMRDI	175	18.5	1	11	11	3	3	1.515	1.089
218	tr|A0A087X079|A0A087X079_	Ig gamma-1 chain C region OS=Homo sapiens GN	62850	MEFGLSW	3604	35.1	-	315	1	13	1	---	---
219	tr|A0A087WW89|A0A087WW	Protein IGHV3-72 OS=Homo sapiens GN=IGHV3	13100	EVQLVES(	387	49.5	-	12	1	4	1	---	---
220	tr|Q6NUL6|Q6NUL6_HUMAN	PITPNA protein (Fragment) OS=Homo sapiens GN	44984	PQRRPAP.	25	5.1	-	1	1	1	1	---	---
221	sp|P01008|ANT3_HUMAN	Antithrombin-III OS=Homo sapiens GN=SERPIN(	64585	MYSNVIG	7673	52.8	-	676	676	25	25	0.925	0.996
222	tr|A0A024R9Q1|A0A024R9Q1	Thrombospondin 1, isoform CRA_a OS=Homo sap	150297	MGLAWG	1658	25.5	-	214	8	27	1	1.307	0.968
223	tr|Q6PIQ7|Q6PIQ7_HUMAN	IGL@ protein OS=Homo sapiens GN=IGL@ PE=	29910	MAWALLI	1404	38.1	-	186	8	8	1	---	---
224	tr|A0A087WU43|A0A087WU4	Cadherin-1 OS=Homo sapiens GN=CDH1 PE=1 S	79244	MGPWSRS	34	2.9	10	2	2	2	2	---	---
225	tr|A0A075B6H6|A0A075B6H6	Ig kappa chain C region (Fragment) OS=Homo sap	14622	XTVAAPS	1005	80.4	-	74	45	6	2	0.791	1.012
226	tr|A0A024R972|A0A024R972_	Laminin, gamma 1 (Formerly LAMB2), isoform C	204490	MDECTDE	53	1	1	1	1	1	1	---	---
227	tr|S6BGE0|S6BGE0_HUMAN	IgG H chain OS=Homo sapiens PE=2 SV=1	37846	MEFGLSW	1429	22.7	-	130	2	5	1	---	---
228	sp|P35527|K1C9_HUMAN	Keratin, type I cytoskeletal 9 OS=Homo sapiens G	70468	MSCRQFS	62	10.4	-	7	7	5	5	0.63	0.618
229	tr|B3KQ18|B3KQ18_HUMAN	cDNA FLJ32632 fis, clone SYNOV1000190, high	56595	MLKKQSA	53	2	4	2	2	1	1	---	---
230	sp|P02747|C1QC_HUMAN	Complement C1q subcomponent subunit C OS=Ho	29940	MDVGPSS	716	21.2	-	50	50	4	4	1.129	0.934
231	sp|P15169|CBPN_HUMAN	Carboxypeptidase N catalytic chain OS=Homo sap	58014	MSDLLSV	37	2	-	4	4	1	1	---	---
232	tr|A0A068LKQ0|A0A068LKQ(	Ig heavy chain variable region (Fragment) OS=Hor	15284	EVQLVQS(	181	10	-	8	8	1	1	0.98	1.073
233	sp|P23560|BDNF_HUMAN	Brain-derived neurotrophic factor OS=Homo sapie	33675	MTILFLTM	48	6.1	-	1	1	1	1	---	---
234	tr|B2R7N9|B2R7N9_HUMAN	cDNA, FLJ93532, highly similar to Homo sapiens	57509	MGFLSPIY	41	2.4	1	1	1	1	1	---	---
235	sp|P13645|K1C10_HUMAN	Keratin, type I cytoskeletal 10 OS=Homo sapiens (	66321	MSVRYSS	504	13.5	-	23	22	7	6	0.468	0.442
236	tr|B2RDL6|B2RDL6_HUMAN	cDNA, FLJ96669, highly similar to Homo sapiens	41811	MRAWIFF	160	7.9	4	15	15	3	3	1.205	1.163
237	sp|P0C0L4|CO4A_HUMAN	Complement C4-A OS=Homo sapiens GN=C4A P	217989	MRLLWGI	18599	42	-	1660	16	65	2	0.79	0.622
238	tr|E7EQB2|E7EQB2_HUMAN	Lactotransferrin (Fragment) OS=Homo sapiens GN	92148	MGLCLAC	602	30.2	2	52	52	18	18	2.479	2.434
239	sp|P07360|CO8G_HUMAN	Complement component C8 gamma chain OS=Hon	24260	MLPPGTA	327	55	-	28	28	9	9	1.086	0.94
240	tr|S6BAR0|S6BAR0_HUMAN	IgG L chain OS=Homo sapiens PE=2 SV=1	27347	MAWTPLF	1409	34.7	-	168	29	7	2	0.988	0.958
241	tr|V9GYE7|V9GYE7_HUMAN	Complement factor H-related protein 2 OS=Homo	34920	MWLLVSV	444	24.8	1	37	7	6	2	1.319	1.074
242	tr|B2R7D2|B2R7D2_HUMAN	cDNA, FLJ93389, highly similar to Homo sapiens	64534	MLRAPGC	36	2.1	2	2	2	1	1	---	---
243	tr|C9J8S2|C9J8S2_HUMAN	Retinoic acid receptor responder protein 2 (Fragme	21794	MRRLLIPL	118	14.5	1	7	7	2	2	1.025	0.994
244	tr|B4E1Z4|B4E1Z4_HUMAN	Uncharacterized protein OS=Homo sapiens PE=2 S	168440	MGPLMVI	349	10.3	1	40	40	13	13	0.965	0.971
245	sp|P0DJI8|SAA1_HUMAN	Serum amyloid A-1 protein OS=Homo sapiens GN	15406	MKLLTGL	125	20.5	-	8	7	2	1	1.061	0.906
246	tr|Q5J875|Q5J875_HUMAN	GUGU gamma form OS=Homo sapiens GN=GUG	41888	MGLFLPL.	47	8.5	3	7	7	3	3	0.264	0.215
247	sp|P0C0L5|CO4B_HUMAN	Complement C4-B OS=Homo sapiens GN=C4B P	217898	MRLLWGI	19149	42	-	1692	38	65	2	0.844	0.653
248	tr|A0A087X232|A0A087X232_	Complement C1s subcomponent OS=Homo sapiens	89564	MYGEILSI	364	16.1	3	53	53	11	11	1.125	1.08
249	sp|P02743|SAMP_HUMAN	Serum amyloid P-component OS=Homo sapiens G	29440	MNKPLLW	926	34.1	-	102	102	9	9	1.086	1.111
250	tr|C8C504|C8C504_HUMAN	Beta-globin OS=Homo sapiens GN=HBB PE=3 S	20056	MVHLTPE	157	35.4	1	14	14	5	5	1.11	0.965
251	sp|O00391|QSOX1_HUMAN	Sulfhydryl oxidase 1 OS=Homo sapiens GN=QSO	93667	MRRCNSG	373	21.3	-	61	61	16	16	0.816	0.801
252	sp|P22792|CPN2_HUMAN	Carboxypeptidase N subunit 2 OS=Homo sapiens (	65328	MLPGAWI	167	15.2	-	26	26	8	8	1.162	1.118
253	tr|H3BN62|H3BN62_HUMAN	Breast cancer anti-estrogen resistance protein 1 (Fr	28074	XPQFQSPI	30	7.1	-	1	1	1	1	---	---
254	tr|Q5NV90|Q5NV90_HUMAN	V2-17 protein (Fragment) OS=Homo sapiens GN=	11730	SYELTQPI	145	41.2	-	16	2	3	1	---	---
255	sp|P02790|HEMO_HUMAN	Hemopexin OS=Homo sapiens GN=HPX PE=1 S\	59077	MARVLGA	33	6.5	-	3	3	3	3	---	---
256	sp|P01768|HV307_HUMAN	Ig heavy chain V-III region CAM OS=Homo sapie	15294	QVELVES(	87	21.3	-	12	12	3	3	1.168	1.32
257	sp|Q5SNT2|TM201_HUMAN	Transmembrane protein 201 OS=Homo sapiens GI	79528	MEGVSAL	29	1.5	-	2	2	1	1	---	---
258	sp|P68366|TBA4A_HUMAN	Tubulin alpha-4A chain OS=Homo sapiens GN=T	56718	MRECISVI	81	9.8	-	5	1	3	1	---	---
259	tr|A2NYU7|A2NYU7_HUMAN	Heavy chain Fab (Fragment) OS=Homo sapiens P	16036	QVKLLES(	26	7.1	-	1	1	1	1	---	---
260	sp|P43251|BTD_HUMAN	Biotinidase OS=Homo sapiens GN=BTD PE=1 S\	68090	MAHAHIQ	145	5.7	-	12	12	3	3	0.999	0.919
261	tr|A0A096LPE2|A0A096LPE2_	Protein SAA2-SAA4 OS=Homo sapiens GN=SAA	26856	MKLLTGL	304	21.2	-	60	59	4	3	0.759	0.724
262	tr|A0A024R498|A0A024R498_	Serpin peptidase inhibitor, clade E (Nexin, plasmin	53326	MNWHLPI	36	9.1	2	3	3	3	3	1.064	1.187
263	tr|B7Z8Q2|B7Z8Q2_HUMAN	cDNA FLJ55606, highly similar to Alpha-2-HS-gl	53042	MFAGCFF	264	13.4	2	24	24	5	5	0.99	1.033
264	tr|B2R815|B2R815_HUMAN	cDNA, FLJ93695, highly similar to Homo sapiens	57184	MHLIDYL	167	17.1	1	25	25	7	7	0.774	0.751
265	sp|P13671|CO6_HUMAN	Complement component C6 OS=Homo sapiens GN	128749	MARRSVL	809	19.5	-	77	77	18	18	1.06	1.011
266	sp|Q4L235|ACSF4_HUMAN	Acyl-CoA synthetase family member 4 OS=Homo	147330	MTLQELV	32	0.7	4	6	6	1	1	---	---
267	tr|B3KPS3|B3KPS3_HUMAN	cDNA FLJ32131 fis, clone PEBLM2000267, high	52809	MPSDKTI(	100	15.4	8	7	3	4	2	1.209	1.279
268	tr|Q4ZFW8|Q4ZFW8_HUMAN	Putative uncharacterized protein IHH OS=Homo sa	34275	MNQWPG	29	4.1	1	1	1	1	1	---	---
269	sp|P00742|FA10_HUMAN	Coagulation factor X OS=Homo sapiens GN=F10	66408	MGRPLHL	37	11.7	-	6	5	6	5	---	---
270	sp|P01023|A2MG_HUMAN	Alpha-2-macroglobulin OS=Homo sapiens GN=A2	191992	MGKNKLI	479	10.3	-	41	31	14	11	1.113	1.116
271	tr|A8K7Q1|A8K7Q1_HUMAN	cDNA FLJ77770, highly similar to Homo sapiens	62976	MPPSGPR(	55	10.2	2	5	5	4	4	1.074	0.921
272	tr|Q53GU8|Q53GU8_HUMAN	Transforming growth factor, beta-induced, 68kDa	85574	MALFVRL	69	9.2	1	6	6	6	6	1.021	0.876
273	sp|Q03591|FHR1_HUMAN	Complement factor H-related protein 1 OS=Homo	43938	MWLLVSV	957	33	-	97	20	10	2	1.343	1.296
274	tr|A2RTY6|A2RTY6_HUMAN	Inter-alpha (Globulin) inhibitor H2 OS=Homo sapi	126903	MKRLTCF	1439	27.7	3	145	145	24	24	1.103	1.127
275	sp|P00451|FA8_HUMAN	Coagulation factor VIII OS=Homo sapiens GN=F8	316692	MQIELST(	129	3.1	-	10	10	7	7	1.019	0.918
276	tr|Q6U2L6|Q6U2L6_HUMAN	C4B (Fragment) OS=Homo sapiens GN=C4B PE=	11768	RSMQGGL	306	15	-	53	52	4	3	0.744	0.707
277	tr|B7Z3Y2|B7Z3Y2_HUMAN	cDNA FLJ51879, highly similar to Prenylcysteine	56786	MVQGQE	40	5.2	5	2	2	2	2	---	---
278	tr|A0A024R1G8|A0A024R1G8	Apolipoprotein L, 1, isoform CRA_b OS=Homo sa	54476	MRFKSHT	561	20	11	63	63	9	9	0.359	0.325
279	tr|A0A024RDB8|A0A024RDB8	Heparanase, isoform CRA_a OS=Homo sapiens G	73287	MLLRSKP.	105	6.6	2	14	14	4	4	1.427	1.471
280	tr|A0A087WSY6|A0A087WSY	Protein IGKV3D-15 (Fragment) OS=Homo sapien	13346	MEAPAQL	49	25.9	-	3	1	2	1	---	---
281	tr|A0A024R3W6|A0A024R3W	Neuropilin 2, isoform CRA_h OS=Homo sapiens (	115991	MDMFPLT	59	1.1	7	2	2	1	1	0.88	0.865
282	sp|Q07954|LRP1_HUMAN	Prolow-density lipoprotein receptor-related protein	576082	MLTPPLLI	243	3.5	-	29	29	14	14	1.193	1.208
283	tr|A0A024R592|A0A024R592_	Glucosidase, alpha neutral AB, isoform CRA_b OS	104019	MTRFRIDI	52	3.1	7	6	6	3	3	---	---
284	tr|A2J1N7|A2J1N7_HUMAN	Rheumatoid factor RF-ET10 (Fragment) OS=Hom	13037	VESGGGL	24	7.3	-	4	4	1	1	---	---
285	tr|A0A024R462|A0A024R462_	Fibronectin 1, isoform CRA_n OS=Homo sapiens (	286673	MLRGPGP	10599	36.3	-	863	66	64	9	1.11	0.968
286	tr|A0A075B6I1|A0A075B6I1_I	Protein IGLV4-60 (Fragment) OS=Homo sapiens (	14671	MAWTPLL	19	7.5	1	1	1	1	1	---	---
287	tr|Q5T9B7|Q5T9B7_HUMAN	Adenylate kinase isoenzyme 1 OS=Homo sapiens (	29708	MGCCSSS	40	5.7	2	1	1	1	1	---	---
288	tr|Q53FV4|Q53FV4_HUMAN	Lumican variant (Fragment) OS=Homo sapiens PE	46640	MSLSAFTI	253	17.5	1	35	35	7	7	1.047	1.043
289	tr|B2R892|B2R892_HUMAN	cDNA, FLJ93793, highly similar to Homo sapiens	54181	MPFGNTH	256	8.1	2	12	12	2	2	0.976	0.94
290	tr|Q6N093|Q6N093_HUMAN	Putative uncharacterized protein DKFZp686I0419(	55234	FFNYAMH	1173	28.1	-	132	1	9	1	---	---
291	tr|Q86TT1|Q86TT1_HUMAN	Full-length cDNA clone CS0DD006YL02 of Neur	47597	MQGTDEI	6862	43.7	-	516	8	15	1	1.026	0.902
292	tr|A8K5T0|A8K5T0_HUMAN	cDNA FLJ75416, highly similar to Homo sapiens (	168175	MRLLAKII	2190	29.7	-	270	5	32	1	1.214	1.176
293	tr|H0Y485|H0Y485_HUMAN	Insulin-like growth factor-binding protein 3 (Fragm	22968	XESPSVSS	48	20.3	14	6	6	3	3	1.494	1.263
294	sp|P05546|HEP2_HUMAN	Heparin cofactor 2 OS=Homo sapiens GN=SERPI	67548	MKHSLNA	699	28.1	-	115	115	16	16	0.473	0.441
295	sp|Q8NBP7|PCSK9_HUMAN	Proprotein convertase subtilisin/kexin type 9 OS=H	80589	MGTVSSR	198	7.1	-	14	14	4	4	1.034	0.838
296	sp|P05154|IPSP_HUMAN	Plasma serine protease inhibitor OS=Homo sapiens	53365	MQLFLLL(	1239	31.3	-	88	88	13	13	0.9	0.856
297	sp|Q6P988|NOTUM_HUMAN	Protein notum homolog OS=Homo sapiens GN=N(	61196	MGRGVR\	60	2.4	2	2	2	1	1	---	---
298	tr|B2R8A1|B2R8A1_HUMAN	cDNA, FLJ93802, highly similar to Homo sapiens	42151	MVSGSSG	91	3	4	17	17	1	1	0.938	0.814
299	tr|A0A024R0A9|A0A024R0A9	Stromal cell derived factor 4, isoform CRA_b OS=	46142	MVWPWV	59	8.6	3	5	5	3	3	0.988	1.012
300	tr|F2RM37|F2RM37_HUMAN	Coagulation factor IX OS=Homo sapiens GN=F9 p	61901	MQRVNM	56	10	1	4	1	4	1	---	---
301	tr|B0AZL7|B0AZL7_HUMAN	cDNA, FLJ79457, highly similar to Insulin-like gr	70047	MALRKG(	48	5.3	2	4	4	3	3	---	---
302	tr|B2R8I2|B2R8I2_HUMAN	cDNA, FLJ93914, highly similar to Homo sapiens	67498	MKALIAA	211	22.1	1	29	29	11	11	1.28	1.223
303	tr|Q5CAQ5|Q5CAQ5_HUMAN	Tumor rejection antigen (Gp96) 1 OS=Homo sapie	116600	MRALWVI	294	17.2	1	33	30	14	13	1.054	0.834
304	tr|A0A024RDF8|A0A024RDF8	Alcohol dehydrogenase 4 (Class II), pi polypeptide	51465	MGTKGK\	41	6.8	2	3	3	3	3	---	---
305	tr|A0A087X0N5|A0A087X0N5	Protein IGKV1-17 OS=Homo sapiens GN=IGKV1	11818	DIQMTQSI	161	35.1	2	9	4	2	1	0.876	1.015
306	tr|Q6N030|Q6N030_HUMAN	Putative uncharacterized protein DKFZp686I1521	68162	MDWTWR	1925	18.1	6	173	49	9	3	1.197	1.145
307	tr|F5GY80|F5GY80_HUMAN	Complement component C8 beta chain OS=Homo	70611	MDTCMTI	214	14	6	41	41	10	10	0.869	0.957
308	tr|A0A0A0MS51|A0A0A0MS5	Gelsolin OS=Homo sapiens GN=GSN PE=4 SV=1	96753	MAEEEAL	655	18.4	6	64	64	12	12	0.678	0.645
309	sp|O00602|FCN1_HUMAN	Ficolin-1 OS=Homo sapiens GN=FCN1 PE=1 SV	42205	MELSGAT	99	8.9	-	9	9	3	3	1.321	1.366
310	tr|C9JPG5|C9JPG5_HUMAN	Semaphorin-3F OS=Homo sapiens GN=SEMA3F	86066	MYVGSKI	40	0.9	2	5	5	1	1	0.627	0.636
311	tr|Q8TBA7|Q8TBA7_HUMAN	HSP90AA1 protein (Fragment) OS=Homo sapiens	97299	GIGMTKA	40	4.5	3	3	2	3	2	---	---
312	tr|B2R6V9|B2R6V9_HUMAN	cDNA, FLJ93141, highly similar to Homo sapiens	95566	MSETSRT.	760	21.7	1	75	75	15	15	0.956	0.95
313	sp|Q9BWP8|COL11_HUMAN	Collectin-11 OS=Homo sapiens GN=COLEC11 PI	35130	MRGNLAI	114	8.9	-	10	10	3	3	1.041	1.074
314	sp|P00747|PLMN_HUMAN	Plasminogen OS=Homo sapiens GN=PLG PE=1 S	108457	MEHKEV\	1079	33.8	-	107	107	26	26	1.187	1.109
315	tr|B3KUE5|B3KUE5_HUMAN	Phospholipid transfer protein, isoform CRA_c OS=	62359	MGLSGSD	390	18.3	2	33	33	9	9	0.438	0.444
316	tr|A0A075B6R9|A0A075B6R9_	Protein IGKV2D-24 (Fragment) OS=Homo sapien	14040	MRLLAQL	160	16.7	-	8	7	2	1	1.346	1.158
317	tr|A0A087WYR4|A0A087WYI	Immunoglobulin lambda-like polypeptide 5 OS=Hc	29663	MAWALLI	865	34	-	101	10	7	1	0.974	1.02
318	tr|B2R950|B2R950_HUMAN	cDNA, FLJ94213, highly similar to Homo sapiens	187153	MRKDRLL	592	16.2	1	70	60	21	18	1.107	1.002
319	tr|A0N5G5|A0N5G5_HUMAN	Rheumatoid factor D5 light chain (Fragment) OS=I	13785	EIVLTQSP.	207	22.9	-	13	5	2	1	0.834	0.934
320	tr|H0Y750|H0Y750_HUMAN	Renin receptor (Fragment) OS=Homo sapiens GN=	31350	XCPAGPFI	47	3.3	5	4	4	1	1	---	---
321	sp|Q15389|ANGP1_HUMAN	Angiopoietin-1 OS=Homo sapiens GN=ANGPT1 I	68085	MTVFLSF.	44	4.8	-	2	2	2	2	---	---
322	tr|A0A024RAB6|A0A024RAB(	Heparan sulfate proteoglycan 2 (Perlecan), isoform	495433	MRWTHSY	44	0.4	1	1	1	1	1	---	---
323	tr|F6KPG5|F6KPG5_HUMAN	Albumin (Fragment) OS=Homo sapiens PE=2 SV=	86736	DAHKSEV	4839	52.8	1	415	415	32	32	1.133	1.122

324	tr|A2N011|A2N011_HUMAN	Vh1-D-J3-region (Fragment) OS=Homo sapiens P	15986	QVHLVQS	125	9.4	-	8	8	1	1	0.981	0.909
325	tr|B4DPQ3|B4DPQ3_HUMAN	cDNA FLJ51034, highly similar to Vitamin K-dep	66152	MAAGRRT	518	19	3	60	23	10	6	1.153	1.161
326	tr|V9GYG9|V9GYG9_HUMAN	Apolipoprotein A-II (Fragment) OS=Homo sapiens	14103	MKLLAAT	186	38.9	2	60	60	3	3	0.603	0.615
327	sp|P11021|GRP78_HUMAN	78 kDa glucose-regulated protein OS=Homo sapien	91263	MKLSLVA	59	5.7	-	4	4	3	3	1.068	0.99
328	tr|B2R5S1|B2R5S1_HUMAN	cDNA, FLJ92595, highly similar to Homo sapiens	60156	MRKRAPQ	350	9.5	6	15	15	4	4	0.857	0.825
329	tr|A0A087WU91|A0A087WU9	Protein IGHV3-49 OS=Homo sapiens GN=IGHV3	14625	AILKGVQ	59	17.5	2	5	5	2	2	---	---
330	tr|H0YAC1|H0YAC1_HUMAN	Plasma kallikrein heavy chain (Fragment) OS=Hor	91778	XSEDCEV	95	8.6	1	9	9	6	6	0.965	0.982
331	sp|Q06033|ITIH3_HUMAN	Inter-alpha-trypsin inhibitor heavy chain H3 OS=H	118324	MAFAWW	320	9.2	-	33	33	7	7	0.962	1.004
332	sp|Q8WWZ8|OIT3_HUMAN	Oncoprotein-induced transcript 3 protein OS=Hom	68290	MPPFLLLT	100	4	-	7	7	2	2	1.26	0.993
333	sp|P15907|SIAT1_HUMAN	Beta-galactoside alpha-2,6-sialyltransferase 1 OS=	58648	MIHTNLKI	46	3.7	-	1	1	1	1	---	---
334	tr|H0YM48|H0YM48_HUMAN	ATP-dependent Clp protease ATP-binding subunit	37716	MPSCGAC	21	2.6	2	1	1	1	1	---	---
335	sp|P05543|THBG_HUMAN	Thyroxine-binding globulin OS=Homo sapiens GN	55459	MSPFLYL	38	4.6	-	3	3	2	2	---	---
336	tr|E7ESK7|E7ESK7_HUMAN	14-3-3 protein zeta/delta (Fragment) OS=Homo sa	20060	MDKNELV	51	14.6	2	3	3	2	2	---	---
337	tr|I3L145|I3L145_HUMAN	Sex hormone-binding globulin OS=Homo sapiens	41647	MTFDLTK	39	12.5	1	9	9	4	4	---	---
338	tr|Q15430|Q15430_HUMAN	Coagulation factor V (Fragment) OS=Homo sapien	29475	PPESTVM	68	8.9	-	3	1	2	1	---	---
339	tr|F5H1C6|F5H1C6_HUMAN	Fermitin family homolog 3 (Fragment) OS=Homo	38768	MAGMKT	46	7.7	1	3	3	2	2	---	---
340	tr|B2R608|B2R608_HUMAN	cDNA, FLJ92718, highly similar to Homo sapiens	64480	MGLQACI	24	3.7	5	3	3	2	2	---	---
341	tr|Q8IZD7|Q8IZD7_HUMAN	Anti-thyroglobulin heavy chain variable region (Fr	15338	QVQLQQS	38	6.9	-	1	1	1	1	---	---
342	sp|P33908|MA1A1_HUMAN	Mannosyl-oligosaccharide 1,2-alpha-mannosidase	86231	MPVGGLL	32	2.5	-	2	2	1	1	---	---
343	tr|G5E9W0|G5E9W0_HUMAN	Phospholipase A1 member A OS=Homo sapiens G	56514	MHKSSSIV	30	3	1	2	2	2	2	---	---
344	tr|C0JYY2|C0JYY2_HUMAN	Apolipoprotein B (Including Ag(X) antigen) OS=H	625263	MDPPRPA	22843	45.3	-	2373	1080	198	94	0.397	0.395
345	tr|E1B4S7|E1B4S7_HUMAN	Apolipoprotein B (Fragment) OS=Homo sapiens G	30390	PTVSSSMI	1108	50	-	65	1	9	1	---	---
346	tr|Q6GMX6|Q6GMX6_HUMA	IGH@ protein OS=Homo sapiens GN=IGH@ PE=	62325	MKHLWFI	3567	35.3	-	314	5	13	2	---	---
347	tr|D3DNU8|D3DNU8_HUMAN	Kininogen 1, isoform CRA_a OS=Homo sapiens G	59249	MKLITILF	208	25.1	3	31	31	10	10	0.835	0.757
348	tr|B4DVE1|B4DVE1_HUMAN	cDNA FLJ53478, highly similar to Galectin-3-bin	70995	MTPPRLF	808	19.4	1	93	93	12	12	1.019	1.105
349	tr|A0A5E4|A0A5E4_HUMAN	Uncharacterized protein OS=Homo sapiens PE=2 S	28363	MAWMMI	1459	35.3	-	173	14	7	1	1.594	1.193
350	sp|Q13103|SPP24_HUMAN	Secreted phosphoprotein 24 OS=Homo sapiens GN	27041	MISRMEK	138	15.6	-	14	14	3	3	1.208	1.231
351	tr|B4E335|B4E335_HUMAN	cDNA FLJ52842, highly similar to Actin, cytoplas	45627	MDDDIAA	366	33.6	7	42	42	9	9	1.33	1.27
352	tr|X6RLJ0|X6RLJ0_HUMAN	Complement C1q subcomponent subunit A (Fragm	27065	MEGPRGV	58	11.8	1	8	8	3	3	1.178	1.254
353	tr|Q5T4F6|Q5T4F6_HUMAN	Cartilage acidic protein 1 (Fragment) OS=Homo sa	60780	YALRDRQ	74	4.2	1	1	1	1	1	---	---
354	tr|B3KWZ6|B3KWZ6_HUMAN	cDNA FLJ44343 fis, clone TRACH3005479, high	150152	MAPEDKD	32	0.6	1	6	6	2	2	---	---
355	tr|B4DNS6|B4DNS6_HUMAN	cDNA FLJ54278, highly similar to SPARC-like pr	71190	MKTGLFF	116	9.6	4	13	13	4	4	1.336	1.124
356	sp|P13667|PDIA4_HUMAN	Protein disulfide-isomerase A4 OS=Homo sapiens	93307	MRPRKAF	23	1.7	-	1	1	1	1	---	---
357	tr|Q6N095|Q6N095_HUMAN	Putative uncharacterized protein DKFZp686K031	63050	MDWTWR	3542	25.1	-	308	2	10	1	---	---
358	tr|B3KUI5|B3KUI5_HUMAN	Hyaluronidase OS=Homo sapiens PE=2 SV=1	51312	MAAHLLP	196	11.5	2	14	14	4	4	1.042	0.988
359	tr|A0A087WV47|A0A087WV4	Ig gamma-1 chain C region OS=Homo sapiens GN	62206	MELGLSW	4000	36.5	1	342	1	14	1	---	---
360	sp|P10720|PF4V_HUMAN	Platelet factor 4 variant OS=Homo sapiens GN=PI	14207	MSSAARS	548	49	-	53	8	4	2	1.076	1.139
361	tr|A0A087WT59|A0A087WT5	Transthyretin OS=Homo sapiens GN=TTR PE=3 S	23099	MASHRLL	813	21.1	4	56	56	5	5	1.202	1.129
362	sp|P55056|APOC4_HUMAN	Apolipoprotein C-IV OS=Homo sapiens GN=APO	17623	MSLLRNR	288	20.5	-	35	35	3	3	0.708	0.668
363	sp|P11678|PERE_HUMAN	Eosinophil peroxidase OS=Homo sapiens GN=EP	88347	MHLLPAL	43	3.9	-	8	1	3	1	---	---
364	sp|P06888|LV109_HUMAN	Ig lambda chain V-I region EPS OS=Homo sapiens	13042	QSVLTQP	39	14.7	-	2	1	2	1	---	---
365	tr|Q8IZZ5|Q8IZZ5_HUMAN	Coagulation factor XII-Mie OS=Homo sapiens PE	76113	MRALLLL	71	3.3	1	3	3	1	1	1.214	0.934
366	tr|Q0VAS5|Q0VAS5_HUMAN	Histone H4 OS=Homo sapiens GN=HIST1H4H P	15015	MSGRGK	41	23.3	1	2	2	2	2	---	---
367	tr|D3DP13|D3DP13_HUMAN	Fibrinogen beta chain, isoform CRA_e OS=Homo	48076	MYLLKDL	199	24.4	3	19	19	8	8	1.334	0.857
368	sp|P02649|APOE_HUMAN	Apolipoprotein E OS=Homo sapiens GN=APOE P	40505	MKVLWA	3398	64	-	373	325	23	21	1.17	1.149
369	tr|H0YJW9|H0YJW9_HUMAN	Uncharacterized protein (Fragment) OS=Homo sap	18921	PGQVDAA	41	31.8	-	5	1	3	1	---	---
370	tr|A0A024R962|A0A024R962_	HCG40889, isoform CRA_b OS=Homo sapiens G	168295	MRLLAKII	2249	30.5	1	277	12	33	2	1.072	1.227
371	sp|Q4KWH8|PLCH1_HUMAN	1-phosphatidylinositol 4,5-bisphosphate phosphodi	231978	MADLEVY	30	0.5	-	1	1	1	1	---	---
372	tr|A0A024R5Z9|A0A024R5Z9_	Pyruvate kinase OS=Homo sapiens GN=PKM2 PE	70098	MSKPHSE	56	4.9	6	3	3	3	3	---	---
373	tr|H0YDT2|H0YDT2_HUMAN	Cathepsin W (Fragment) OS=Homo sapiens GN=	27350	ALTAHPS	29	6.2	2	1	1	1	1	---	---
374	tr|Q9UL88|Q9UL88_HUMAN	Myosin-reactive immunoglobulin heavy chain varia	17289	EVQLVES	71	19.8	-	6	4	2	1	1.031	0.992
375	sp|O43866|CD5L_HUMAN	CD5 antigen-like OS=Homo sapiens GN=CD5L P	44774	MALLFSLI	484	43.8	-	37	37	12	12	1.081	1.02
376	tr|Q9UL84|Q9UL84_HUMAN	Myosin-reactive immunoglobulin heavy chain varia	15510	EVQLVES	60	14.8	-	3	1	2	1	---	---
377	tr|G3XAP6|G3XAP6_HUMAN	Cartilage oligomeric matrix protein OS=Homo sap	88600	MLRELQE	51	1.4	8	5	5	1	1	---	---
378	tr|A0A0A0MSV6|A0A0A0MS	Complement C1q subcomponent subunit B (Fragm	29198	MKIPWGS	760	23.1	2	63	63	6	6	0.976	0.873
379	sp|P06753|TPM3_HUMAN	Tropomyosin alpha-3 chain OS=Homo sapiens GN	44851	MMEAIKK	40	8.4	-	3	1	2	1	---	---
380	tr|Q5IWS5|Q5IWS5_HUMAN	Intelectin 1 OS=Homo sapiens GN=ITLN1 PE=2 S	39480	MNQLSFL	96	6.4	1	12	12	2	2	1.203	1.042
381	tr|Q59HB3|Q59HB3_HUMAN	Apolipoprotein B variant (Fragment) OS=Homo sa	222441	TIEGPLTSI	7264	45	-	713	7	67	1	0.468	0.416
382	tr|A0A024QZL1|A0A024QZL1	Proteoglycan 1, secretory granule, isoform CRA_a	19609	MMQKLLI	60	8.2	1	3	3	1	1	---	---
383	tr|A0A087WSY5|A0A087WSY	Carboxypeptidase B2 OS=Homo sapiens GN=CPE	51257	MKLCSLA	157	18.7	1	22	22	7	7	1.064	0.768
384	tr|E1B2D1|E1B2D1_HUMAN	Hemoglobin alpha-1 globin chain variant (Fragmer	13799	MVLSPAD	63	22	7	4	4	2	2	---	---
385	tr|A0A075B6K6|A0A075B6K6	Protein IGLV4-3 OS=Homo sapiens GN=IGLV4-	15013	MAWVSFY	22	13.9	1	4	4	2	2	---	---
386	tr|Q6MZL2|Q6MZL2_HUMAN	Putative uncharacterized protein DKFZp686M056	38922	NPGIYTCS	103	12.8	-	10	9	4	3	1.362	1.527
387	tr|Q6P528|Q6P528_HUMAN	ASPN protein OS=Homo sapiens GN=ASPN PE=	54606	MKEYVLL	26	2.9	1	1	1	1	1	---	---
388	tr|D6RHH7|D6RHH7_HUMAN	Corticotropin-releasing factor-binding protein OS=	30563	MSPNFKL	48	8	1	4	4	2	2	1.539	1.112
389	tr|M0R009|M0R009_HUMAN	Alpha-1B-glycoprotein (Fragment) OS=Homo sapi	37179	LTCQAHL	103	14.7	1	11	11	5	5	0.994	0.965
390	tr|A0A0A0MRQ5|A0A0A0MR	Peroxiredoxin-1 OS=Homo sapiens GN=PRDX1 I	14073	MSSGNAK	29	8.2	5	1	1	1	1	---	---
391	sp|P02775|CXCL7_HUMAN	Platelet basic protein OS=Homo sapiens GN=PPB	18429	MSLRLDT	164	37.5	-	11	11	4	4	1.149	1.167

A-VS-B@C	A-VS-B@C	A-VS-B@S	A-VS-B@S	KEGG(ko_	GO Biologi	GO Molecu	GO Cellular_Component
-	-	-	-	K01344|1|9	GO:000759	GO:000425	GO:0005788//endoplasmic reticulum lumen;GO:0005796//Golgi lumen;GO:0005576//extracellular region
-	-	-	-	K14462|1|0	GO:001088	GO:005075	GO:0030669//clathrin-coated endocytic vesicle membrane;GO:0034360//chylomicron remnant;GO:0005788//endoplasmic reticulum lumen
4	4	*	*	K07605|1|0	GO:003298	GO:000520	GO:0005794//Golgi apparatus;GO:0045095//keratin filament
-	-	-	-	K04593|1|1	GO:000727	-	GO:0005576//extracellular region
33	33	*	-	K08023|1|8	GO:001095	GO:001650	GO:0005615//extracellular space;GO:0005604//basement membrane
-	-	-	-	K11252|1|1	GO:000633	GO:000367	GO:0000786//nucleosome;GO:0005634//nucleus
55	55	-	-	K03098|1|6	GO:004866	GO:003609	GO:0005783//endoplasmic reticulum;GO:0005615//extracellular space;GO:0022626//cytosolic ribosome;GO:0048471//perinuclear region
106	106	*	-	K05399|1|0	GO:003272	GO:000510	GO:0005615//extracellular space
174	174	-	-	K05316|1|8	GO:001095	GO:000486	GO:0005737//cytoplasm;GO:0005886//plasma membrane;GO:0005576//extracellular region
2	2	-	-	K01172|1|1	GO:004866	GO:000550	GO:0030426//growth cone;GO:0005615//extracellular space;GO:0005605//basal lamina;GO:0032311//angiogenin-PRI complex;GO:0005
-	-	-	-	K12472|1|4	GO:004801	GO:000508	GO:0005829//cytosol;GO:0030054//cell junction;GO:0043005//neuron projection;GO:0045202//synapse;GO:0005905//coated pit;GO:003
-	-	-	-	-	-	-	
2	2	-	-	K08784|1|0	GO:003051	GO:000425	GO:0005615//extracellular space;GO:0031012//extracellular matrix;GO:0005829//cytosol
1	1	-	-	K06554|1|5	-	-	
15	14	-	*	K14616|1|7	GO:000257	-	GO:0031093//platelet alpha granule lumen;GO:0005576//extracellular region
18	18	*	*	K14477|1|0	GO:000695	GO:003049	GO:0031838//haptoglobin-hemoglobin complex;GO:0034366//spherical high-density lipoprotein particle
-	-	-	-	K07937|1|9	GO:001603	GO:000505	GO:0000139//Golgi membrane;GO:0005829//cytosol;GO:0048471//perinuclear region of cytoplasm;GO:0005886//plasma membrane;GO:
1	1	-	-	-	GO:000686	GO:000531	GO:0034362//low-density lipoprotein particle;GO:0034364//high-density lipoprotein particle
2	2	-	-	K02727|1|3	GO:000020	GO:000551	GO:0005654//nucleoplasm;GO:0005829//cytosol;GO:0019773//proteasome core complex, alpha-subunit complex
1	1	-	-	K06554|1|2	-	-	
-	-	-	-	K06554|1|2	-	-	
1	1	-	-	K06554|1|4	GO:004508	GO:000382	GO:0005886//plasma membrane;GO:0005576//extracellular region
1	1	-	-	K06553|1|5	-	-	
3	3	-	-	K03984|1|5	GO:001095	GO:000486	GO:0005576//extracellular region
3	3	-	*	-	GO:000268	GO:000551	GO:0005615//extracellular space
2	2	-	-	K12258|1|0	GO:005072	GO:000510	GO:0005829//cytosol;GO:0005615//extracellular space;GO:0030424//axon;GO:0043202//lysosomal lumen;GO:0048471//perinuclear regi
14	14	*	-	K04524|1|9	GO:001087	GO:003121	GO:0042627//chylomicron;GO:0034361//very-low-density lipoprotein particle;GO:0034364//high-density lipoprotein particle;GO:000578
1	1	-	-	K06827|1|5	GO:000155	GO:000552	GO:0005615//extracellular space;GO:0005886//plasma membrane
50	50	-	-	K13624|1|0	GO:000682	GO:005108	GO:0005615//extracellular space
1	1	-	-	K06856|1|9	-	-	
2	2	-	*	-	GO:003334	GO:000554	GO:0034362//low-density lipoprotein particle;GO:0034365//discoidal high-density lipoprotein particle;GO:0034361//very-low-density lipo
5	4	-	*	K08130|1|3	-	-	GO:0005576//extracellular region
-	-	-	-	K12382|1|3	GO:000166	-	GO:0042599//lamellar body;GO:0005764//lysosome;GO:0005771//multivesicular body;GO:0005615//extracellular space
1	1	-	-	K06856|1|3	-	-	
21	21	-	-	K05502|1|7	GO:001095	GO:000820	GO:0005615//extracellular space;GO:0031012//extracellular matrix
3	3	*	-	K06856|1|2	-	-	
7	7	*	*	K13915|1|5	GO:004274	GO:000379	GO:0005615//extracellular space
2	2	*	-	K08632|1|1	GO:003019	GO:000202	GO:0005614//interstitial matrix
-	-	-	-	K02842|1|0	GO:000635	GO:000493	GO:0031527//filopodium membrane;GO:0016021//integral to membrane;GO:0009986//cell surface;GO:0048471//perinuclear region of cy
2	2	-	-	K01172|1|1	GO:000637	GO:000452	GO:0005576//extracellular region
1	1	-	-	K11275|1|1	GO:001658	GO:000367	GO:0005694//chromosome;GO:0005634//nucleus
6	6	-	-	-	-	-	
23	23	*	*	K01672|1|7	GO:000166	GO:004687	GO:0005615//extracellular space
10	10	-	-	K01127|1|0	GO:004306	GO:000462	GO:0005615//extracellular space;GO:0043231//intracellular membrane-bounded organelle;GO:0005578//proteinaceous extracellular matr
-	-	-	-	K12499|1|1	GO:007144	GO:004362	GO:0005737//cytoplasm;GO:0005615//extracellular space
11	11	*	*	K04391|1|0	GO:000690	GO:007089	GO:0045121//membrane raft;GO:0005615//extracellular space;GO:0031225//anchored to membrane;GO:0009986//cell surface;GO:0005
-	-	-	-	K06554|1|6	-	-	
2	2	-	-	K05502|1|0	GO:000662	GO:000827	GO:0031988//membrane-bounded vesicle;GO:0005581//collagen;GO:0005615//extracellular space
5	5	-	-	K01059|1|0	GO:003437	GO:000446	GO:0042627//chylomicron;GO:0009986//cell surface;GO:0031012//extracellular matrix;GO:0034361//very-low-density lipoprotein partic
8	8	*	-	K08648|1|0	GO:000715	GO:000553	GO:0005615//extracellular space
4	4	*	*	-	GO:000609	GO:003463	GO:0005615//extracellular space
19	20	*	-	K03908|1|0	GO:003016	GO:000550	GO:0000139//Golgi membrane;GO:0031093//platelet alpha granule lumen;GO:0005789//endoplasmic reticulum membrane;GO:0005576//
4	4	-	-	K01327|1|2	GO:004211	GO:000425	GO:0042582//azurophil granule;GO:0005576//extracellular region
-	-	-	-	K06554|1|6	-	-	
1	1	-	-	K06554|1|6	GO:004508	GO:000382	GO:0005886//plasma membrane;GO:0005576//extracellular region
-	-	-	-	K14678|1|0	GO:001827	-	GO:0005576//extracellular region;GO:0031012//extracellular matrix
-	-	-	-	K10352|1|0	GO:003022	GO:004280	GO:0005826//actomyosin contractile ring;GO:0001772//immunological synapse;GO:0031594//neuromuscular junction;GO:0005829//cyto
1	1	-	-	K07604|1|0	GO:001021	GO:000551	GO:0005829//cytosol;GO:0005739//mitochondrion;GO:0005730//nucleolus;GO:0071944//cell periphery;GO:0045095//keratin filament
17	17	-	-	K01045|1|0	GO:004639	GO:004687	GO:0005615//extracellular space
1	1	-	-	K07375|1|0	GO:004226	GO:000552	GO:0045298//tubulin complex;GO:0005829//cytosol;GO:0005874//microtubule;GO:0005634//nucleus
9	9	-	*	K01774|1|1	GO:000905	GO:000406	GO:0016021//integral to membrane;GO:0009986//cell surface
1	1	-	-	K01350|1|8	GO:009702	GO:001989	GO:0005829//cytosol;GO:0031012//extracellular matrix;GO:0005886//plasma membrane
4	4	-	-	K04667|1|0	GO:004000	GO:000808	GO:0005576//extracellular region
-	-	-	-	K06856|1|1	-	-	
-	-	-	-	K10373|1|1	GO:003461	GO:004280	GO:0032059//bleb;GO:0005862//muscle thin filament tropomyosin;GO:0005829//cytosol;GO:0031941//filamentous actin;GO:0001725//s
3	3	-	-	K03982|1|0	GO:000717	GO:000486	GO:0005615//extracellular space;GO:0005886//plasma membrane;GO:0031093//platelet alpha granule lumen;GO:0031012//extracellular
26	26	*	-	K03984|1|0	GO:003398	GO:000486	GO:0005615//extracellular space;GO:0031093//platelet alpha granule lumen;GO:0005578//proteinaceous extracellular matrix
15	15	-	-	K00518|1|6	GO:004668	GO:000820	GO:0005615//extracellular space;GO:0031012//extracellular matrix;GO:0005634//nucleus;GO:0005829//cytosol;GO:0005802//trans-Gol
-	-	-	-	K03989|1|1	GO:000695	GO:000486	GO:0005615//extracellular space;GO:0005886//plasma membrane
-	-	-	-	K10847|1|4	-	-	
21	22	-	-	K00650|1|0	GO:004215	GO:000460	GO:0034364//high-density lipoprotein particle
2	2	-	-	K05604|1|0	GO:000650	GO:004687	GO:0005576//extracellular region
-	-	-	-	K06249|1|0	GO:002176	GO:007032	GO:0005615//extracellular space;GO:0005737//cytoplasm;GO:0005578//proteinaceous extracellular matrix;GO:0030425//dendrite
-	-	-	-	K06856|1|7	-	-	
59	59	-	-	K03989|1|2	GO:000695	GO:000486	GO:0005615//extracellular space;GO:0005886//plasma membrane
25	25	*	-	K04003|1|2	GO:000695	-	GO:0005576//extracellular region;GO:0005886//plasma membrane
-	-	-	-	K06554|1|7	-	-	
4	4	-	*	K04004|1|8	GO:000686	GO:000531	GO:0005615//extracellular space
17	17	*	*	K05399|1|1	GO:004369	GO:001712	GO:0031982//vesicle;GO:0034364//high-density lipoprotein particle
3	3	-	-	K04525|1|0	GO:001095	GO:000486	GO:0005576//extracellular region;GO:0005634//nucleus
2	2	-	-	K13375|1|0	GO:000717	GO:000808	GO:0030424//axon;GO:0043025//neuronal cell body;GO:0005902//microvillus;GO:0005796//Golgi lumen;GO:0009986//cell surface;GO
1	1	-	-	K00683|1|7	GO:000650	GO:001660	GO:0005576//extracellular region
5	4	*	*	-	GO:001026	GO:000843	GO:0005615//extracellular space
2	2	-	-	-	-	GO:000828	GO:0005615//extracellular space
2	2	*	-	K03996|1|0	GO:000695	-	GO:0005579//membrane attack complex;GO:0005576//extracellular region
1	1	-	-	K08767|1|4	GO:000663	GO:000517	GO:0005615//extracellular space
21	21	*	*	K04004|1|1	GO:000695	-	GO:0005576//extracellular region
1	1	-	-	K06554|1|2	-	-	
-	-	-	-	K06554|1|2	-	-	
-	-	-	-	K06856|1|6	-	-	
-	-	-	-	K10031|1|1	GO:007009	GO:004523	GO:0005615//extracellular space;GO:0009897//external side of plasma membrane
12	12	-	-	K15412|1|0	GO:004274	-	GO:0005615//extracellular space;GO:0031012//extracellular matrix
1	1	-	-	K03386|1|1	GO:004274	GO:000837	GO:0005829//cytosol;GO:0005739//mitochondrion
1	1	-	-	K03906|1|0	GO:000759	-	GO:0005576//extracellular region
1	1	-	-	K06554|1|1	-	-	
-	-	-	-	K06856|1|2	-	-	
50	51	*	-	K05407|1|2	GO:001652	GO:000820	GO:0031093//platelet alpha granule lumen;GO:0005615//extracellular space
9	9	*	*	K09025|1|0	GO:004572	GO:005075	GO:0042627//chylomicron;GO:0034361//very-low-density lipoprotein particle;GO:0034364//high-density lipoprotein particle;GO:003436
1	1	-	-	K01046|1|0	GO:000663	GO:000462	GO:0034364//high-density lipoprotein particle
197	197	-	-	K08760|1|0	GO:004274	GO:004280	GO:0009986//cell surface;GO:0034364//high-density lipoprotein particle;GO:0042627//chylomicron;GO:0005788//endoplasmic reticulum
5	3	-	*	K04001|1|2	GO:003016	GO:000551	GO:0031093//platelet alpha granule lumen;GO:0005615//extracellular space
1	1	-	-	K02731|1|8	GO:000020	GO:000429	GO:0005654//nucleoplasm;GO:0005829//cytosol;GO:0019773//proteasome core complex, alpha-subunit complex
9	9	-	-	K09580|1|0	GO:000666	GO:000375	GO:0005793//endoplasmic reticulum-Golgi intermediate compartment;GO:0009986//cell surface;GO:0042470//melanosome;GO:0005788
-	-	-	-	K15288|1|1	-	-	
21	22	*	-	K07605|1|0	GO:000697	GO:000487	GO:0045095//keratin filament;GO:0005886//plasma membrane

<table>
<tr><td>19</td><td>19</td><td>-</td><td>-</td><td>K03903|1|0</td><td>GO:003016</td><td>GO:003067</td><td>GO:0009897//external side of plasma membrane;GO:0031093//platelet alpha granule lumen;GO:0005938//cell cortex;GO:0005577//fibrin</td></tr>
<tr><td>1</td><td>1</td><td>-</td><td>-</td><td>K05699|1|0</td><td>GO:003016</td><td>GO:000517</td><td>GO:0005925//focal adhesion;GO:0031143//pseudopodium;GO:0031093//platelet alpha granule lumen;GO:0005576//extracellular region;G</td></tr>
<tr><td>1</td><td>1</td><td>-</td><td>-</td><td>-</td><td>GO:000859</td><td>GO:000820</td><td>GO:0005578//proteinaceous extracellular matrix</td></tr>
<tr><td>1</td><td>1</td><td>-</td><td>-</td><td>-</td><td>GO:003016</td><td>GO:000550</td><td>GO:0033018//sarcoplasmic reticulum lumen;GO:0042470//melanosome;GO:0005576//extracellular region;GO:0005794//Golgi apparatus</td></tr>
<tr><td>191</td><td>191</td><td>-</td><td>-</td><td>-</td><td>GO:006107</td><td>GO:003162</td><td>GO:0034366//spherical high-density lipoprotein particle;GO:0031093//platelet alpha granule lumen;GO:0031966//mitochondrial membran</td></tr>
<tr><td>-</td><td>-</td><td>-</td><td>-</td><td>K06856|1|2</td><td>-</td><td>-</td><td></td></tr>
<tr><td>5</td><td>5</td><td>-</td><td>-</td><td>-</td><td>GO:005088</td><td>GO:000550</td><td>GO:0030658//transport vesicle membrane;GO:0030141//secretory granule;GO:0005576//extracellular region</td></tr>
<tr><td>-</td><td>-</td><td>-</td><td>-</td><td>K06856|1|1</td><td>-</td><td>-</td><td></td></tr>
<tr><td>3</td><td>3</td><td>-</td><td>-</td><td>K10138|1|2</td><td>GO:004434</td><td>GO:000552</td><td>GO:0005615//extracellular space</td></tr>
<tr><td>5</td><td>5</td><td>-</td><td>*</td><td>K06554|1|2</td><td>-</td><td>-</td><td></td></tr>
<tr><td>26</td><td>26</td><td>-</td><td>*</td><td>-</td><td>GO:000196</td><td>GO:000487</td><td>GO:0005615//extracellular space;GO:0005578//proteinaceous extracellular matrix</td></tr>
<tr><td>2</td><td>2</td><td>-</td><td>-</td><td>K06856|1|8</td><td>-</td><td>-</td><td></td></tr>
<tr><td>3</td><td>3</td><td>*</td><td>-</td><td>K06826|1|0</td><td>GO:000716</td><td>GO:004323</td><td>GO:0005605//basal lamina;GO:0071944//cell periphery</td></tr>
<tr><td>7</td><td>7</td><td>*</td><td>-</td><td>K01446|1|0</td><td>GO:003282</td><td>GO:000827</td><td>GO:0005622//intracellular;GO:0005576//extracellular region;GO:0016020//membrane</td></tr>
<tr><td>1</td><td>1</td><td>-</td><td>-</td><td>K06831|1|7</td><td>GO:000838</td><td>GO:001986</td><td>GO:0005681//spliceosomal complex;GO:0005743//mitochondrial inner membrane;GO:0005578//proteinaceous extracellular matrix;GO:0</td></tr>
<tr><td>9</td><td>9</td><td>-</td><td>*</td><td>K09644|1|0</td><td>GO:003016</td><td>GO:000425</td><td>GO:0031093//platelet alpha granule lumen;GO:0034358//plasma lipoprotein particle;GO:0031232//extrinsic to external side of plasma me</td></tr>
<tr><td>8</td><td>8</td><td>*</td><td>*</td><td>K14736|1|0</td><td>GO:005508</td><td>GO:000819</td><td>GO:0030141//secretory granule;GO:0009925//basal plasma membrane;GO:0005739//mitochondrion;GO:0030139//endocytic vesicle;GO:</td></tr>
<tr><td>15</td><td>14</td><td>-</td><td>*</td><td>K03905|1|0</td><td>GO:003016</td><td>GO:003067</td><td>GO:0009897//external side of plasma membrane;GO:0031093//platelet alpha granule lumen;GO:0005938//cell cortex;GO:0005577//fibrin</td></tr>
<tr><td>4</td><td>4</td><td>*</td><td>*</td><td>K06260|1|0</td><td>GO:000716</td><td>GO:000551</td><td>GO:0005887//integral to plasma membrane;GO:0009986//cell surface</td></tr>
<tr><td>9</td><td>8</td><td>-</td><td>*</td><td>K07296|1|4</td><td>GO:003303</td><td>GO:004349</td><td>GO:0005783//endoplasmic reticulum;GO:0005615//extracellular space;GO:0071944//cell periphery;GO:0048471//perinuclear region of cy</td></tr>
<tr><td>1</td><td>1</td><td>-</td><td>-</td><td>K08023|1|4</td><td>GO:001904</td><td>GO:000520</td><td>GO:0005615//extracellular space;GO:0005578//proteinaceous extracellular matrix</td></tr>
<tr><td>3</td><td>3</td><td>-</td><td>-</td><td>K08627|1|0</td><td>GO:003016</td><td>GO:000827</td><td>GO:0005615//extracellular space;GO:0009986//cell surface;GO:0005578//proteinaceous extracellular matrix</td></tr>
<tr><td>18</td><td>18</td><td>-</td><td>*</td><td>K03983|1|0</td><td>GO:000203</td><td>GO:004280</td><td>GO:0005577//fibrinogen complex;GO:0031093//platelet alpha granule lumen</td></tr>
<tr><td>1</td><td>1</td><td>-</td><td>-</td><td>K05717|1|0</td><td>GO:001095</td><td>GO:001650</td><td>GO:0016324//apical plasma membrane;GO:0005604//basement membrane</td></tr>
<tr><td>29</td><td>29</td><td>-</td><td>-</td><td>-</td><td>GO:004295</td><td>GO:006023</td><td>GO:0042627//chylomicron;GO:0034366//spherical high-density lipoprotein particle;GO:0034361//very-low-density lipoprotein particle;G</td></tr>
<tr><td>1</td><td>1</td><td>-</td><td>-</td><td>K06856|1|4</td><td>-</td><td>-</td><td></td></tr>
<tr><td>-</td><td>-</td><td>-</td><td>-</td><td>-</td><td>-</td><td>-</td><td></td></tr>
<tr><td>-</td><td>-</td><td>-</td><td>-</td><td>K06554|1|5</td><td>GO:004508</td><td>GO:000382</td><td>GO:0005886//plasma membrane;GO:0005576//extracellular region</td></tr>
<tr><td>10</td><td>10</td><td>*</td><td>-</td><td>K03997|1|0</td><td>GO:000695</td><td>-</td><td>GO:0005615//extracellular space;GO:0005579//membrane attack complex</td></tr>
<tr><td>7</td><td>7</td><td>*</td><td>-</td><td>-</td><td>GO:003016</td><td>GO:000166</td><td>GO:0034364//high-density lipoprotein particle</td></tr>
<tr><td>7</td><td>6</td><td>*</td><td>*</td><td>K08023|1|1</td><td>GO:001810</td><td>GO:000550</td><td>GO:0005615//extracellular space;GO:0005578//proteinaceous extracellular matrix</td></tr>
<tr><td>39</td><td>39</td><td>*</td><td>-</td><td>K06856|1|1</td><td>-</td><td>-</td><td></td></tr>
<tr><td>1</td><td>1</td><td>-</td><td>-</td><td>-</td><td>-</td><td>-</td><td></td></tr>
<tr><td>2</td><td>2</td><td>-</td><td>-</td><td>K10375|1|2</td><td>GO:000697</td><td>GO:000830</td><td>GO:0005829//cytosol;GO:0030863//cortical cytoskeleton;GO:0002102//podosome;GO:0001725//stress fiber;GO:0005862//muscle thin fil</td></tr>
<tr><td>1</td><td>1</td><td>-</td><td>-</td><td>K05717|1|0</td><td>GO:000152</td><td>GO:001650</td><td>GO:0005604//basement membrane;GO:0005793//endoplasmic reticulum-Golgi intermediate compartment;GO:0031093//platelet alpha gra</td></tr>
<tr><td>35</td><td>35</td><td>-</td><td>*</td><td>K01045|1|0</td><td>GO:001087</td><td>GO:000406</td><td>GO:0034366//spherical high-density lipoprotein particle</td></tr>
<tr><td>-</td><td>-</td><td>-</td><td>-</td><td>K08672|1|0</td><td>GO:000735</td><td>GO:004349</td><td>GO:0005796//Golgi lumen;GO:0005615//extracellular space;GO:0009986//cell surface;GO:0012505//endomembrane system;GO:003101</td></tr>
<tr><td>2</td><td>2</td><td>-</td><td>-</td><td>K05717|1|8</td><td>GO:000152</td><td>GO:001650</td><td>GO:0005604//basement membrane;GO:0005793//endoplasmic reticulum-Golgi intermediate compartment;GO:0031093//platelet alpha gra</td></tr>
<tr><td>5</td><td>5</td><td>*</td><td>-</td><td>-</td><td>GO:000268</td><td>GO:000551</td><td>GO:0005615//extracellular space</td></tr>
<tr><td>1</td><td>1</td><td>-</td><td>-</td><td>-</td><td>GO:000635</td><td>GO:000367</td><td>GO:0005576//extracellular region;GO:0005634//nucleus</td></tr>
<tr><td>-</td><td>-</td><td>-</td><td>-</td><td>K09631|1|0</td><td>GO:000650</td><td>GO:000425</td><td>GO:0005615//extracellular space;GO:0005791//rough endoplasmic reticulum</td></tr>
<tr><td>-</td><td>-</td><td>-</td><td>-</td><td>K06856|1|1</td><td>GO:000695</td><td>GO:000382</td><td>GO:0005576//extracellular region;GO:0016021//integral to membrane;GO:0005886//plasma membrane</td></tr>
<tr><td>1</td><td>1</td><td>-</td><td>-</td><td>K03984|1|4</td><td>GO:001095</td><td>GO:000486</td><td>GO:0005576//extracellular region</td></tr>
<tr><td>-</td><td>-</td><td>-</td><td>-</td><td>-</td><td>-</td><td>-</td><td></td></tr>
<tr><td>85</td><td>85</td><td>-</td><td>-</td><td>K04861|1|2</td><td>GO:003021</td><td>GO:000486</td><td>GO:0005576//extracellular region</td></tr>
<tr><td>1</td><td>1</td><td>-</td><td>-</td><td>K05656|1|1</td><td>GO:000191</td><td>GO:004697</td><td>GO:0042825//TAP complex;GO:0005743//mitochondrial inner membrane;GO:0005765//lysosomal membrane;GO:0005769//early endoso</td></tr>
<tr><td>1</td><td>1</td><td>-</td><td>-</td><td>K06554|1|7</td><td>-</td><td>-</td><td></td></tr>
<tr><td>307</td><td>307</td><td>-</td><td>-</td><td>K01313|1|0</td><td>GO:000759</td><td>GO:000808</td><td>GO:0005788//endoplasmic reticulum lumen;GO:0005615//extracellular space;GO:0005886//plasma membrane;GO:0005796//Golgi lumen</td></tr>
<tr><td>3</td><td>4</td><td>*</td><td>-</td><td>K06251|1|3</td><td>GO:000695</td><td>GO:003024</td><td>GO:0005576//extracellular region;GO:0016021//integral to membrane</td></tr>
<tr><td>1</td><td>1</td><td>-</td><td>-</td><td>K06856|1|3</td><td>-</td><td>-</td><td></td></tr>
<tr><td>-</td><td>-</td><td>-</td><td>-</td><td>K04524|1|3</td><td>GO:000664</td><td>GO:005075</td><td>GO:0031232//extrinsic to external side of plasma membrane;GO:0034364//high-density lipoprotein particle;GO:0042627//chylomicron;G</td></tr>
<tr><td>1</td><td>1</td><td>-</td><td>-</td><td>K06554|1|4</td><td>-</td><td>-</td><td></td></tr>
<tr><td>2</td><td>2</td><td>-</td><td>-</td><td>K04659|1|0</td><td>GO:001652</td><td>GO:007005</td><td>GO:0005577//fibrinogen complex;GO:0009897//external side of plasma membrane;GO:0031093//platelet alpha granule lumen;GO:00310</td></tr>
<tr><td>-</td><td>-</td><td>-</td><td>-</td><td>-</td><td>GO:006028</td><td>-</td><td>GO:0005737//cytoplasm;GO:0005929//cilium</td></tr>
<tr><td>20</td><td>20</td><td>*</td><td>-</td><td>K03993|1|0</td><td>GO:000695</td><td>GO:000425</td><td>GO:0005576//extracellular region</td></tr>
<tr><td>1</td><td>1</td><td>-</td><td>-</td><td>K07860|1|4</td><td>GO:000741</td><td>GO:000552</td><td>GO:0005829//cytosol;GO:0005635//nuclear envelope;GO:0005886//plasma membrane</td></tr>
<tr><td>112</td><td>112</td><td>*</td><td>-</td><td>K04002|1|0</td><td>GO:004508</td><td>GO:000551</td><td>GO:0005737//cytoplasm;GO:0005886//plasma membrane;GO:0005576//extracellular region</td></tr>
<tr><td>-</td><td>-</td><td>-</td><td>-</td><td>-</td><td>GO:006048</td><td>GO:000551</td><td>GO:0005829//cytosol;GO:0005730//nucleolus;GO:0000922//spindle pole;GO:0005876//spindle microtubule;GO:0005694//chromosome;G</td></tr>
<tr><td>1</td><td>1</td><td>-</td><td>-</td><td>K04525|1|2</td><td>GO:001095</td><td>GO:000486</td><td>GO:0005737//cytoplasm;GO:0005576//extracellular region;GO:0016020//membrane</td></tr>
<tr><td>2</td><td>2</td><td>-</td><td>-</td><td>K08023|1|0</td><td>GO:003558</td><td>GO:000502</td><td>GO:0005578//proteinaceous extracellular matrix</td></tr>
<tr><td>-</td><td>-</td><td>-</td><td>-</td><td>K07249|1|0</td><td>GO:003232</td><td>GO:000549</td><td>GO:0005829//cytosol</td></tr>
<tr><td>2</td><td>2</td><td>-</td><td>-</td><td>K01333|1|0</td><td>GO:000695</td><td>GO:000504</td><td>GO:0005615//extracellular space;GO:0016020//membrane</td></tr>
<tr><td>676</td><td>676</td><td>-</td><td>-</td><td>K03990|1|0</td><td>GO:000250</td><td>GO:003171</td><td>GO:0005615//extracellular space;GO:0005886//plasma membrane</td></tr>
<tr><td>87</td><td>87</td><td>-</td><td>-</td><td>K08759|1|6</td><td>GO:000718</td><td>GO:001548</td><td>GO:0042627//chylomicron;GO:0034366//spherical high-density lipoprotein particle;GO:0034361//very-low-density lipoprotein particle;G</td></tr>
<tr><td>1</td><td>1</td><td>-</td><td>-</td><td>K06554|1|6</td><td>-</td><td>-</td><td></td></tr>
<tr><td>-</td><td>-</td><td>-</td><td>-</td><td>-</td><td>GO:003016</td><td>GO:000166</td><td>GO:0034364//high-density lipoprotein particle</td></tr>
<tr><td>55</td><td>56</td><td>*</td><td>*</td><td>K03994|1|0</td><td>GO:003360</td><td>GO:003171</td><td>GO:0005579//membrane attack complex;GO:0005615//extracellular space</td></tr>
<tr><td>3</td><td>3</td><td>-</td><td>-</td><td>K10138|1|7</td><td>GO:000698</td><td>GO:000552</td><td>GO:0005615//extracellular space</td></tr>
<tr><td>-</td><td>-</td><td>-</td><td>-</td><td>K06856|1|4</td><td>-</td><td>-</td><td></td></tr>
<tr><td>1</td><td>1</td><td>-</td><td>-</td><td>K01062|1|0</td><td>GO:003444</td><td>GO:000384</td><td>GO:0034362//low-density lipoprotein particle</td></tr>
<tr><td>43</td><td>43</td><td>*</td><td>-</td><td>K00432|1|1</td><td>GO:004274</td><td>GO:000460</td><td>GO:0005615//extracellular space</td></tr>
<tr><td>9</td><td>9</td><td>-</td><td>*</td><td>K03992|1|0</td><td>GO:000650</td><td>GO:000550</td><td>GO:0005615//extracellular space</td></tr>
<tr><td>-</td><td>-</td><td>-</td><td>-</td><td>K06856|1|2</td><td>GO:000695</td><td>GO:000382</td><td>GO:0005576//extracellular region;GO:0016021//integral to membrane;GO:0005886//plasma membrane</td></tr>
<tr><td>3</td><td>3</td><td>*</td><td>*</td><td>-</td><td>GO:002241</td><td>GO:000487</td><td>GO:0005789//endoplasmic reticulum membrane;GO:0005886//plasma membrane;GO:0016021//integral to membrane;GO:0005576//extrac</td></tr>
<tr><td>7</td><td>7</td><td>-</td><td>-</td><td>K03909|1|2</td><td>GO:004216</td><td>GO:000486</td><td>GO:0005576//extracellular region;GO:0005886//plasma membrane</td></tr>
<tr><td>2</td><td>2</td><td>-</td><td>-</td><td>K04667|1|0</td><td>GO:004000</td><td>GO:000808</td><td>GO:0005576//extracellular region</td></tr>
<tr><td>-</td><td>-</td><td>-</td><td>-</td><td>K10360|1|6</td><td>GO:003285</td><td>GO:000507</td><td>GO:0005829//cytosol;GO:0043231//intracellular membrane-bounded organelle;GO:0001726//ruffle;GO:0005886//plasma membrane</td></tr>
<tr><td>1</td><td>1</td><td>-</td><td>-</td><td>-</td><td>GO:003439</td><td>GO:000550</td><td>GO:0005615//extracellular space;GO:0005578//proteinaceous extracellular matrix;GO:0005737//cytoplasm;GO:0016020//membrane;GO:</td></tr>
<tr><td>8</td><td>8</td><td>-</td><td>*</td><td>K06238|1|0</td><td>GO:000741</td><td>GO:000486</td><td>GO:0030133//transport vesicle;GO:0005615//extracellular space;GO:0005788//endoplasmic reticulum lumen;GO:0042383//sarcolemma;G</td></tr>
<tr><td>-</td><td>-</td><td>-</td><td>-</td><td>K06554|1|5</td><td>-</td><td>-</td><td></td></tr>
<tr><td>5</td><td>4</td><td>-</td><td>*</td><td>-</td><td>GO:000695</td><td>GO:000382</td><td>GO:0005576//extracellular region</td></tr>
<tr><td>1</td><td>1</td><td>-</td><td>-</td><td>K06554|1|6</td><td>-</td><td>-</td><td></td></tr>
<tr><td>103</td><td>103</td><td>-</td><td>-</td><td>K03902|1|0</td><td>GO:003016</td><td>GO:000550</td><td>GO:0005615//extracellular space;GO:0031093//platelet alpha granule lumen;GO:0005886//plasma membrane</td></tr>
<tr><td>27</td><td>27</td><td>*</td><td>-</td><td>K01330|1|0</td><td>GO:004508</td><td>GO:000425</td><td>GO:0005576//extracellular region</td></tr>
<tr><td>2</td><td>2</td><td>-</td><td>-</td><td>K11251|1|9</td><td>GO:000633</td><td>GO:000367</td><td>GO:0000786//nucleosome;GO:0005634//nucleus</td></tr>
<tr><td>6</td><td>6</td><td>*</td><td>*</td><td>K10789|1|0</td><td>GO:000695</td><td>GO:004687</td><td>GO:0005764//lysosome;GO:0030141//secretory granule;GO:0005615//extracellular space;GO:0005634//nucleus</td></tr>
<tr><td>31</td><td>31</td><td>*</td><td>-</td><td>K03900|1|0</td><td>GO:003016</td><td>GO:004748</td><td>GO:0009897//external side of plasma membrane;GO:0033093//Weibel-Palade body;GO:0005578//proteinaceous extracellular matrix;GO:</td></tr>
<tr><td>70</td><td>70</td><td>-</td><td>-</td><td>K04000|1|0</td><td>GO:001983</td><td>-</td><td>GO:0005579//membrane attack complex;GO:0005576//extracellular region</td></tr>
<tr><td>2</td><td>2</td><td>*</td><td>-</td><td>K06554|1|5</td><td>-</td><td>-</td><td></td></tr>
<tr><td>-</td><td>-</td><td>-</td><td>-</td><td>K01319|1|1</td><td>GO:007094</td><td>GO:000425</td><td>GO:0030141//secretory granule;GO:0005615//extracellular space;GO:0009986//cell surface;GO:0031012//extracellular matrix;GO:0005</td></tr>
<tr><td>1</td><td>1</td><td>-</td><td>-</td><td>K06238|1|2</td><td>GO:003444</td><td>GO:004687</td><td>GO:0031258//lamellipodium membrane;GO:0016021//integral to membrane;GO:0031527//filopodium membrane</td></tr>
<tr><td>19</td><td>18</td><td>*</td><td>-</td><td>K01883|1|5</td><td>GO:005118</td><td>GO:000843</td><td>GO:0005615//extracellular space</td></tr>
<tr><td>448</td><td>448</td><td>-</td><td>-</td><td>K06251|1|0</td><td>GO:003209</td><td>GO:000517</td><td>GO:0005615//extracellular space;GO:0071062//alphav-beta3 integrin-vitronectin complex;GO:0031012//extracellular matrix</td></tr>
<tr><td>7</td><td>7</td><td>-</td><td>-</td><td>K14477|1|6</td><td>GO:000695</td><td>GO:003049</td><td>GO:0005615//extracellular space;GO:0031838//haptoglobin-hemoglobin complex</td></tr>
<tr><td>2</td><td>2</td><td>-</td><td>-</td><td>K06271|1|0</td><td>GO:000741</td><td>GO:003027</td><td>GO:0005925//focal adhesion;GO:0005911//cell-cell junction;GO:0032587//ruffle membrane;GO:0043231//intracellular membrane-bounde</td></tr>
<tr><td>-</td><td>-</td><td>-</td><td>-</td><td>K06856|1|5</td><td>-</td><td>-</td><td></td></tr>
<tr><td>-</td><td>-</td><td>-</td><td>-</td><td>K06825|1|0</td><td>GO:003558</td><td>GO:000550</td><td>GO:0005604//basement membrane;GO:0005615//extracellular space;GO:0001527//microfibril</td></tr>
<tr><td>-</td><td>-</td><td>-</td><td>-</td><td>K13073|1|0</td><td>-</td><td>-</td><td>GO:0005576//extracellular region;GO:0005887//integral to plasma membrane;GO:0055038//recycling endosome membrane;GO:0030133/</td></tr>
<tr><td>-</td><td>-</td><td>-</td><td>-</td><td>K05901|1|8</td><td>GO:004428</td><td>GO:004260</td><td>GO:0005829//cytosol</td></tr>
<tr><td>7</td><td>7</td><td>*</td><td>*</td><td>K03989|1|3</td><td>GO:000695</td><td>GO:000486</td><td>GO:0005615//extracellular space;GO:0005886//plasma membrane</td></tr>
<tr><td>1</td><td>1</td><td>-</td><td>-</td><td>K06554|1|1</td><td>-</td><td>-</td><td></td></tr>
<tr><td>-</td><td>-</td><td>-</td><td>-</td><td>K03996|1|0</td><td>GO:000695</td><td>-</td><td>GO:0005579//membrane attack complex;GO:0005576//extracellular region</td></tr>
<tr><td>-</td><td>-</td><td>-</td><td>-</td><td>K08288|1|0</td><td>GO:000724</td><td>GO:000508</td><td>GO:0005788//endoplasmic reticulum lumen</td></tr>
<tr><td>14</td><td>14</td><td>*</td><td>*</td><td>K10104|1|7</td><td>GO:000822</td><td>GO:000382</td><td>GO:0005615//extracellular space;GO:0005581//collagen</td></tr>
<tr><td>-</td><td>-</td><td>-</td><td>-</td><td>K01323|1|0</td><td>GO:000759</td><td>GO:000551</td><td>GO:0005615//extracellular space;GO:0005886//plasma membrane</td></tr>
<tr><td>48</td><td>48</td><td>*</td><td>*</td><td>K10104|1|2</td><td>GO:000716</td><td>GO:003024</td><td>GO:0005615//extracellular space;GO:0005581//collagen</td></tr>
<tr><td>-</td><td>-</td><td>-</td><td>-</td><td>K10414|1|0</td><td>GO:003090</td><td>GO:000377</td><td>GO:0035085//cilium axoneme;GO:0005829//cytosol;GO:0030286//dynein complex;GO:0005874//microtubule;GO:0031512//motile prima</td></tr>
<tr><td>1</td><td>1</td><td>-</td><td>-</td><td>-</td><td>GO:000693</td><td>-</td><td>GO:0005737//cytoplasm;GO:0005615//extracellular space</td></tr>
<tr><td>603</td><td>603</td><td>-</td><td>-</td><td>K08757|1|1</td><td>GO:007050</td><td>GO:000554</td><td>GO:0030139//endocytic vesicle;GO:0005788//endoplasmic reticulum lumen;GO:0005886//plasma membrane;GO:0030141//secretory gran</td></tr>
</table>

1	1	-	-	K06553|1|9	-	-	
1	1	-	-	K05460|1|1	GO:000156	GO:000425	GO:0005576//extracellular region;GO:0005829//cytosol;GO:0005634//nucleus
8	8	*	-	K00134|1|3	GO:005140	GO:005128	GO:0005829//cytosol;GO:0015630//microtubule cytoskeleton;GO:0048471//perinuclear region of cytoplasm;GO:0005811//lipid particle;G
1	1	-	-	K06856|1|2	-	-	
-	-	-	-	K06856|1|3	-	-	
-	-	-	-	K01106|1|3	GO:000741	GO:000852	GO:0016021//integral to membrane;GO:0005737//cytoplasm
347	347	-	-	K03911|1|0	GO:005511	GO:000486	GO:0005615//extracellular space;GO:0005971//ribonucleoside-diphosphate reductase complex;GO:0005886//plasma membrane
6	6	*	-	K04659|1|0	GO:001652	GO:007005	GO:0005577//fibrinogen complex;GO:0009897//external side of plasma membrane;GO:0031093//platelet alpha granule lumen;GO:00310
-	-	-	-	K06554|1|2	-	-	
1	1	-	-	K05689|1|0	GO:007097	GO:003050	GO:0009986//cell surface;GO:0016235//aggresome;GO:0043296//apical junction complex;GO:0005925//focal adhesion;GO:0005802//tra
20	20	-	-	K06554|1|2	GO:004508	GO:000382	GO:0005886//plasma membrane;GO:0005576//extracellular region
1	1	-	-	K05635|1|0	GO:001647	GO:004320	GO:0043260//laminin-11 complex;GO:0005606//laminin-1 complex;GO:0005615//extracellular space;GO:0043259//laminin-10 complex
-	-	-	-	K06856|1|1	-	-	
3	3	*	-	K07604|1|1	GO:000728	GO:000520	GO:0045095//keratin filament;GO:0048471//perinuclear region of cytoplasm
1	1	-	-	K00726|1|0	GO:001827	GO:004687	GO:0016021//integral to membrane;GO:0000139//Golgi membrane
25	25	*	-	K03988|1|9	GO:000695	-	GO:0005581//collagen
-	-	-	-	K01292|1|0	GO:000662	GO:000418	GO:0005615//extracellular space
7	7	-	*	K06856|1|8	-	-	
1	1	-	-	K04355|1|6	GO:001404	GO:000808	GO:0016023//cytoplasmic membrane-bounded vesicle;GO:0005576//extracellular region
1	1	-	-	K08124|1|0	GO:004234	-	GO:0043202//lysosomal lumen;GO:0005796//Golgi lumen;GO:0005578//proteinaceous extracellular matrix
14	14	*	*	K07604|1|0	GO:007127	GO:003028	GO:0005737//cytoplasm;GO:0045095//keratin filament
9	9	-	*	K08136|1|3	GO:007136	GO:000550	GO:0005604//basement membrane;GO:0005615//extracellular space;GO:0031093//platelet alpha granule lumen;GO:0005634//nucleus
5	5	*	*	K03989|1|0	GO:000695	GO:000486	GO:0005615//extracellular space;GO:0005886//plasma membrane
26	26	*	*	K14736|1|0	GO:004274	GO:000819	GO:0030141//secretory granule;GO:0097013//phagocytic vesicle lumen;GO:0005576//extracellular region
13	13	-	-	K03999|1|2	GO:000695	GO:001984	GO:0005615//extracellular space;GO:0005579//membrane attack complex
8	8	-	-	K06554|1|5	-	-	
2	2	-	-	K04004|1|3	-	-	GO:0005576//extracellular region
1	1	-	-	K03103|1|0	GO:003028	GO:000399	GO:0005788//endoplasmic reticulum lumen
5	4	-	-	K10044|1|1	GO:005087	GO:000510	GO:0031012//extracellular matrix;GO:0005576//extracellular region
21	21	-	-	K01335|1|0	GO:000695	GO:000425	GO:0005886//plasma membrane;GO:0005576//extracellular region
7	7	-	-	-	GO:003016	GO:000166	GO:0034364//high-density lipoprotein particle
3	3	*	*	K03898|1|8	-	-	GO:0005576//extracellular region
29	29	-	*	K03989|1|0	GO:000695	GO:000486	GO:0005615//extracellular space;GO:0005886//plasma membrane
26	26	*	*	K01331|1|0	GO:004508	GO:000425	GO:0005576//extracellular region
56	56	-	-	K01672|1|2	GO:000645	GO:004687	GO:0005615//extracellular space;GO:0043234//protein complex;GO:0031012//extracellular matrix
5	5	-	-	K13823|1|3	GO:004274	GO:000460	GO:0031838//haptoglobin-hemoglobin complex;GO:0005833//hemoglobin complex
19	18	*	*	K10758|1|0	GO:004545	GO:000905	GO:0005615//extracellular space;GO:0030173//integral to Golgi membrane
9	9	*	*	K13023|1|0	GO:005082	GO:003023	GO:0005576//extracellular region
-	-	-	-	K05726|1|1	GO:004801	GO:000487	GO:0005925//focal adhesion;GO:0030027//lamellipodium;GO:0005829//cytosol;GO:0001726//ruffle
-	-	-	-	K06554|1|6	-	-	
1	1	-	-	K01396|1|3	GO:000687	GO:000551	GO:0005615//extracellular space
2	2	-	-	K06856|1|5	-	-	
-	-	-	-	-	-	-	GO:0016020//membrane
1	1	-	-	K07374|1|0	GO:000022	GO:000551	GO:0005881//cytoplasmic microtubule;GO:0005576//extracellular region;GO:0005829//cytosol
-	-	-	-	K06856|1|1	-	-	
11	11	-	-	K01435|1|0	GO:000741	GO:000407	GO:0005730//nucleolus;GO:0005615//extracellular space;GO:0045177//apical part of cell;GO:0043204//perikaryon
19	19	*	-	-	GO:000695	-	GO:0034364//high-density lipoprotein particle
2	2	-	-	K03982|1|3	GO:004587	GO:000820	GO:0031232//extrinsic to external side of plasma membrane;GO:0031594//neuromuscular junction;GO:0005829//cytosol;GO:0005615//ex
14	13	-	-	-	GO:000150	GO:003029	GO:0005615//extracellular space;GO:0043234//protein complex;GO:0031012//extracellular matrix
11	11	*	*	K04525|1|1	GO:001095	GO:000486	GO:0005615//extracellular space
39	39	-	-	K03995|1|0	GO:001983	GO:000551	GO:0005615//extracellular space;GO:0005579//membrane attack complex
-	-	-	-	K00142|1|0	GO:000663	GO:000552	-
3	3	-	*	K07374|1|0	GO:005108	GO:001990	GO:0030496//midbody;GO:0005881//cytoplasmic microtubule
-	-	-	-	K11989|1|2	GO:000205	GO:000511	GO:0005615//extracellular space;GO:0005886//plasma membrane;GO:0031012//extracellular matrix
-	-	-	-	K01314|1|0	GO:000759	GO:000550	GO:0005815//microtubule organizing center;GO:0005796//Golgi lumen;GO:0031233//intrinsic to external side of plasma membrane;GO:0
20	21	*	*	K03910|1|0	GO:003016	GO:001996	GO:0031093//platelet alpha granule lumen;GO:0005615//extracellular space;GO:0005829//cytosol
2	2	-	-	K12385|1|9	-	GO:000367	GO:0015630//microtubule cytoskeleton;GO:0016020//membrane;GO:0005615//extracellular space;GO:0005793//endoplasmic reticulum-G
3	3	-	-	-	GO:000152	GO:005084	GO:0005615//extracellular space;GO:0005886//plasma membrane;GO:0005578//proteinaceous extracellular matrix
10	10	*	-	K04004|1|6	GO:004508	-	GO:0005615//extracellular space
55	55	*	*	K04861|1|3	GO:003021	GO:000486	GO:0005576//extracellular region
5	5	-	*	K03899|1|0	GO:003016	GO:000550	GO:0005615//extracellular space;GO:0031093//platelet alpha granule lumen;GO:0005886//plasma membrane
10	10	*	*	K03989|1|9	GO:004508	-	GO:0005576//extracellular region;GO:0005886//plasma membrane
1	1	-	-	K05906|1|0	GO:003032	GO:000855	GO:0005764//lysosome;GO:0034361//very-low-density lipoprotein particle;GO:0005886//plasma membrane
32	32	*	*	K14480|1|0	GO:001983	GO:000828	GO:0031224//intrinsic to membrane;GO:0034364//high-density lipoprotein particle;GO:0034361//very-low-density lipoprotein particle
4	4	*	*	K07964|1|0	GO:003369	GO:000456	GO:0045121//membrane raft;GO:0005765//lysosomal membrane;GO:0005576//extracellular region;GO:0043202//lysosomal lumen;GO:0
-	-	-	-	K06554|1|2	-	-	
2	2	-	-	K06819|1|0	GO:000741	GO:001983	GO:0016021//integral to membrane;GO:0005576//extracellular region;GO:0030424//axon;GO:0002116//semaphorin receptor complex;G
16	16	-	*	K04550|1|0	GO:004295	GO:000504	GO:0005905//coated pit;GO:0030425//dendrite;GO:0043025//neuronal cell body;GO:0005768//endosome;GO:0048471//perinuclear regio
1	1	-	-	K05546|1|0	GO:001827	GO:003391	GO:0042470//melanosome;GO:0005788//endoplasmic reticulum lumen;GO:0017177//glucosidase II complex;GO:0005794//Golgi apparat
-	-	-	-	K06856|1|1	-	-	
30	30	-	-	K05717|1|0	GO:000152	GO:001650	GO:0005604//basement membrane;GO:0005793//endoplasmic reticulum-Golgi intermediate compartment;GO:0031093//platelet alpha gra
-	-	-	-	K06553|1|9	-	-	
1	1	-	-	K00939|1|2	GO:000705	GO:000401	GO:0005737//cytoplasm;GO:0005886//plasma membrane
16	16	-	-	K08122|1|0	GO:007084	GO:000520	GO:0005615//extracellular space;GO:0005796//Golgi lumen;GO:0043202//lysosomal lumen;GO:0005583//fibrillar collagen
12	12	-	-	K00933|1|0	GO:000660	GO:000411	GO:0005829//cytosol
1	1	-	-	K06856|1|8	-	-	
7	7	-	-	K06856|1|8	GO:000695	GO:000382	GO:0005576//extracellular region;GO:0016021//integral to membrane;GO:0005886//plasma membrane
2	2	-	-	K04004|1|0	GO:000695	-	GO:0005615//extracellular space
2	2	-	-	K10138|1|2	GO:004356	GO:003199	GO:0042567//insulin-like growth factor ternary complex;GO:0042568//insulin-like growth factor binary complex;GO:0005634//nucleus
33	33	*	-	K03912|1|0	GO:000693	GO:000486	GO:0005576//extracellular region
6	6	-	*	K13050|1|0	GO:004308	GO:003419	GO:0005615//extracellular space;GO:0005764//lysosome;GO:0005794//Golgi apparatus;GO:0005770//late endosome;GO:0005769//early
56	56	-	-	K03913|1|0	GO:001095	GO:000197	GO:0097183//protein C inhibitor-coagulation factor XI complex;GO:0036030//protein C inhibitor-plasma kallikrein complex;GO:003602
1	1	-	-	-	-	GO:001678	GO:0005576//extracellular region
2	2	-	-	K01899|1|4	GO:000610	GO:000387	GO:0045244//succinate-CoA ligase complex (GDP-forming);GO:0005743//mitochondrial inner membrane;GO:0005886//plasma membra
2	2	-	-	-	GO:004547	GO:000550	GO:0005796//Golgi lumen;GO:0032059//bleb;GO:0005770//late endosome;GO:0005886//plasma membrane
-	-	-	-	K01321|1|0	GO:000759	GO:000550	GO:0005796//Golgi lumen;GO:0005788//endoplasmic reticulum lumen;GO:0005576//extracellular region;GO:0005886//plasma membran
1	1	-	-	K13023|1|3	GO:000715	GO:000802	GO:0005730//nucleolus;GO:0042567//insulin-like growth factor ternary complex
18	18	-	*	-	GO:004306	GO:000486	GO:0005886//plasma membrane;GO:0031093//platelet alpha granule lumen;GO:0005576//extracellular region
10	10	-	*	K09487|1|0	GO:003043	GO:004679	GO:0005788//endoplasmic reticulum lumen;GO:0042470//melanosome;GO:0005789//endoplasmic reticulum membrane;GO:0005829//cyt
1	1	-	-	K13980|1|0	GO:004257	GO:005128	GO:0005829//cytosol;GO:0015630//microtubule cytoskeleton
4	4	-	-	K06554|1|9	-	-	
47	47	-	-	K06856|1|4	-	-	
9	10	-	-	K03998|1|0	GO:000695	-	GO:0005615//extracellular space;GO:0005579//membrane attack complex
34	34	*	*	K05768|1|0	GO:005159	GO:000550	GO:0030027//lamellipodium;GO:0015629//actin cytoskeleton;GO:0005615//extracellular space;GO:0005829//cytosol;GO:0001726//ruffl
4	5	*	*	K10104|1|0	GO:000822	GO:000382	GO:0005615//extracellular space;GO:0005581//collagen
5	5	*	*	K06840|1|0	GO:004884	GO:000487	GO:0005615//extracellular space;GO:0016020//membrane
1	1	-	-	K04079|1|0	GO:000741	GO:003091	GO:0042470//melanosome;GO:0005829//cytosol;GO:0005886//plasma membrane
42	42	-	-	K03917|1|0	GO:001814	GO:004687	GO:0031093//platelet alpha granule lumen;GO:0005576//extracellular region
6	5	-	*	K10066|1|2	GO:000727	GO:000553	GO:0005581//collagen
54	55	*	-	K01315|1|0	GO:003016	GO:003418	GO:0031232//extrinsic to external side of plasma membrane;GO:0005615//extracellular space;GO:0031093//platelet alpha granule lumen
21	21	*	*	K08761|1|0	GO:001018	GO:000828	GO:0005576//extracellular region
6	6	*	*	K06554|1|4	-	-	
4	4	-	-	K06554|1|3	GO:004508	GO:000382	GO:0005886//plasma membrane;GO:0005576//extracellular region
27	28	*	-	K03910|1|0	GO:003016	GO:001996	GO:0031093//platelet alpha granule lumen;GO:0005615//extracellular space;GO:0005829//cytosol
5	5	-	-	K06554|1|4	-	-	
-	-	-	-	-	GO:003291	GO:000487	GO:0009897//external side of plasma membrane;GO:0016021//integral to membrane
1	1	-	-	K05465|1|0	GO:003313	GO:003097	GO:0005615//extracellular space;GO:0045121//membrane raft;GO:0005886//plasma membrane;GO:0005902//microvillus
1	1	-	-	K06255|1|0	GO:000602	GO:000802	GO:0005796//Golgi lumen;GO:0005604//basement membrane;GO:0043202//lysosomal lumen;GO:0005615//extracellular space;GO:0005
239	239	-	-	K01883|1|7	GO:004668	GO:005108	GO:0005604//basement membrane;GO:0005814//centriole;GO:0031093//platelet alpha granule lumen;GO:0043296//apical junction comp

8	8	-	-	K06856|1|1	-	-	
10	10	*	*	K01344|1|0	GO:001718	GO:000550	GO:0005796//Golgi lumen;GO:0005615//extracellular space;GO:0005788//endoplasmic reticulum lumen;GO:0005886//plasma membrane
10	10	*	*	K08758|1|2	GO:006069	GO:004280	GO:0042627//chylomicron;GO:0005788//endoplasmic reticulum lumen;GO:0034361//very-low-density lipoprotein particle;GO:0034366//
3	3	-	-	K09490|1|0	GO:003051	GO:005108	GO:0009986//cell surface;GO:0034663//endoplasmic reticulum chaperone complex;GO:0030496//midbody;GO:0005788//endoplasmic ret
11	11	-	*	K09821|1|0	GO:004816	GO:000808	GO:0005615//extracellular space;GO:0044444//cytoplasmic part
1	1	-	-	K06856|1|1	-	-	
4	4	-	-	K01324|1|0	GO:000759	GO:001670	GO:0005615//extracellular space;GO:0005886//plasma membrane
21	21	-	-	K05316|1|4	GO:003021	GO:000486	GO:0005576//extracellular region
4	4	*	-	K13912|1|2	-	GO:000550	GO:0005635//nuclear envelope
1	1	-	-	K00778|1|0	GO:001827	GO:000383	GO:0032580//Golgi cisterna membrane;GO:0030173//integral to Golgi membrane;GO:0005576//extracellular region
-	-	-	-	K03544|1|5	GO:000645	GO:004687	GO:0005730//nucleolus;GO:0005743//mitochondrial inner membrane;GO:0042645//mitochondrial nucleoid;GO:0009841//mitochondrial e
1	1	-	-	K04525|1|2	GO:003469	GO:000486	GO:0005615//extracellular space
1	1	-	-	K06630|1|6	GO:003016	GO:001630	GO:0005654//nucleoplasm;GO:0042470//melanosome;GO:0005829//cytosol;GO:0031252//cell leading edge;GO:0043234//protein comple
1	1	-	-	K05464|1|5	GO:000991	GO:000549	GO:0005576//extracellular region
-	-	-	-	K03902|1|3	GO:003016	GO:000550	GO:0005615//extracellular space;GO:0031093//platelet alpha granule lumen;GO:0005886//plasma membrane
1	1	-	-	-	GO:003362	GO:000517	GO:0002102//podosome;GO:0030054//cell junction;GO:0042995//cell projection
-	-	-	-	K01279|1|0	GO:004236	GO:000843	GO:0005829//cytosol;GO:0005654//nucleoplasm;GO:0000783//nuclear telomere cap complex;GO:0043202//lysosomal lumen;GO:00057З
1	1	-	-	K06856|1|3	-	-	
-	-	-	-	K01230|1|0	GO:001827	GO:000457	GO:0000139//Golgi membrane;GO:0005793//endoplasmic reticulum-Golgi intermediate compartment;GO:0016021//integral to membrane
-	-	-	-	K13618|1|0	GO:000665	GO:000897	GO:0005576//extracellular region
519	519	*	-	K14462|1|0	GO:001088	GO:005075	GO:0030669//clathrin-coated endocytic vesicle membrane;GO:0034360//chylomicron remnant;GO:0005788//endoplasmic reticulum lumer
-	-	-	-	K14462|1|4	GO:001088	GO:005075	GO:0030669//clathrin-coated endocytic vesicle membrane;GO:0034360//chylomicron remnant;GO:0005788//endoplasmic reticulum lumer
1	1	-	-	K06856|1|3	-	-	
12	12	-	*	K03898|1|0	GO:003016	GO:000510	GO:0031093//platelet alpha granule lumen;GO:0005615//extracellular space;GO:0005886//plasma membrane
35	35	-	*	K13912|1|2	GO:000696	GO:000504	GO:0016020//membrane;GO:0005615//extracellular space;GO:0005578//proteinaceous extracellular matrix
3	3	*	-	K06554|1|1	-	-	
5	5	*	*	-	GO:000646	GO:000486	GO:0043234//protein complex;GO:0005576//extracellular region
22	22	*	*	K05692|1|0	GO:000741	GO:001990	GO:0030863//cortical cytoskeleton;GO:0031941//filamentous actin;GO:0005829//cytosol;GO:0030016//myofibril;GO:0030424//axon;GC
2	2	-	*	K03986|1|3	GO:004508	GO:000551	GO:0005602//complement component C1 complex;GO:0005581//collagen
1	1	-	-	-	-	GO:000550	GO:0005578//proteinaceous extracellular matrix
1	1	-	-	-	-	-	GO:0005730//nucleolus;GO:0005929//cilium;GO:0005874//microtubule
8	8	*	-	K08136|1|2	GO:000716	GO:000550	GO:0005615//extracellular space;GO:0005578//proteinaceous extracellular matrix
-	-	-	-	K09582|1|0	GO:004545	GO:000905	GO:0005788//endoplasmic reticulum lumen;GO:0042470//melanosome
-	-	-	-	K06856|1|3	-	-	
10	10	-	-	K01197|1|0	GO:000961	GO:000441	GO:0005615//extracellular space;GO:0031410//cytoplasmic vesicle;GO:0043202//lysosomal lumen;GO:0036117//hyaluranon cable
-	-	-	-	K06856|1|3	-	-	
7	7	-	*	K05407|1|6	GO:001652	GO:000820	GO:0031093//platelet alpha granule lumen;GO:0005615//extracellular space
45	45	*	-	K07127|1|1	-	GO:000551	GO:0005576//extracellular region
15	14	*	*	-	GO:000662	GO:000531	GO:0034364//high-density lipoprotein particle;GO:0034361//very-low-density lipoprotein particle
-	-	-	-	K10788|1|0	GO:004274	GO:004687	-
-	-	-	-	K06554|1|2	-	-	
3	3	-	-	K01328|1|0	GO:000759	GO:005178	GO:0005615//extracellular space;GO:0005886//plasma membrane
1	1	-	-	K11254|1|2	GO:000633	GO:000367	GO:0000786//nucleosome;GO:0005634//nucleus
12	11	*	*	K03904|1|0	GO:003016	GO:003067	GO:0009897//external side of plasma membrane;GO:0031093//platelet alpha granule lumen;GO:0005938//cell cortex;GO:0005577//fibrin
138	138	-	-	K04524|1|5	GO:000664	GO:005075	GO:0031232//extrinsic to external side of plasma membrane;GO:0034364//high-density lipoprotein particle;GO:0042627//chylomicron;GC
-	-	-	-	K06251|1|6	GO:003209	GO:000517	GO:0005615//extracellular space;GO:0071062//alphav-beta3 integrin-vitronectin complex;GO:0031012//extracellular matrix
8	8	-	*	K04004|1|0	GO:000695	-	GO:0005615//extracellular space
-	-	-	-	K05857|1|4	GO:001604	GO:000487	GO:0031965//nuclear membrane;GO:0005829//cytosol
1	1	-	-	K00873|1|0	GO:001631	GO:000474	GO:0005886//plasma membrane;GO:0005634//nucleus;GO:0005829//cytosol;GO:0005739//mitochondrion
-	-	-	-	K08569|1|2	GO:000695	GO:000823	-
3	3	-	-	K06856|1|2	-	-	
24	24	-	-	K13912|1|4	GO:000696	GO:000504	GO:0016020//membrane;GO:0005615//extracellular space
-	-	-	-	K06856|1|3	-	-	
1	1	-	-	K04659|1|0	GO:006017	GO:000820	GO:0005615//extracellular space;GO:0005578//proteinaceous extracellular matrix
43	43	-	*	K03987|1|8	GO:000695	GO:000551	GO:0005576//extracellular region
-	-	-	-	K09290|1|1	GO:003004	-	GO:0005829//cytosol;GO:0030863//cortical cytoskeleton;GO:0002102//podosome;GO:0001725//stress fiber;GO:0005862//muscle thin fil
4	4	-	-	-	GO:004632	GO:000510	GO:0031526//brush border membrane;GO:0045121//membrane raft;GO:0005576//extracellular region;GO:0031225//anchored to membra
7	7	*	*	K14462|1|0	GO:001088	GO:005075	GO:0030669//clathrin-coated endocytic vesicle membrane;GO:0034360//chylomicron remnant;GO:0005788//endoplasmic reticulum lumer
1	1	-	-	K06849|1|1	GO:003338	GO:000551	GO:0000139//Golgi membrane;GO:0031093//platelet alpha granule lumen;GO:0005615//extracellular space;GO:0042588//zymogen granu
4	5	-	-	K01300|1|0	GO:004249	GO:000418	GO:0005615//extracellular space
1	1	-	-	K13822|1|2	GO:004274	GO:000460	GO:0022627//cytosolic small ribosomal subunit;GO:0031838//haptoglobin-hemoglobin complex;GO:0005833//hemoglobin complex
-	-	-	-	K06553|1|2	-	-	
4	4	*	*	K03992|1|0	GO:000650	GO:000550	GO:0005615//extracellular space
-	-	-	-	K08120|1|0	GO:007017	GO:001528	GO:0046930//pore complex;GO:0005578//proteinaceous extracellular matrix
3	3	*	-	-	GO:007127	GO:000551	GO:0031045//dense core granule;GO:0043196//varicosity;GO:0043679//axon terminus;GO:0005615//extracellular space;GO:0005794//G
5	5	-	-	K06512|1|7	-	-	GO:0005576//extracellular region
-	-	-	-	K13279|1|8	GO:000150	GO:000837	GO:0042470//melanosome;GO:0005634//nucleus;GO:0005739//mitochondrion
9	9	-	*	K10029|1|2	GO:003016	GO:000535	GO:0005615//extracellular space;GO:0031093//platelet alpha granule lumen

supplementary table3. A list of dysregulated proteins between group A(EH) and group B(TJT)

Hits	Accession	Desription	Mass	Seq	Score	Cov	SameSets	Spectrum	Unique Spe	Peptide	Unique Pep
1	sp|P35908|K22E_HUMAN	Keratin, type II cytoskeletal 2 epidermal OS=Homo sapiens GN=KRT2	76630	MSCQISCI	275	15.5	-	23	13	10	8
2	tr|B4E0A4|B4E0A4_HUMAN	cDNA FLJ57023, highly similar to Homo sapiens collagen, type VI, al	149266	MRKHRHI	124	4.3	4	12	12	5	5
3	sp|P18428|LBP_HUMAN	Lipopolysaccharide-binding protein OS=Homo sapiens GN=LBP PE=	60518	MGALARA	2335	20.2	-	145	145	9	9
4	tr|L8E853|L8E853_HUMAN	von Willebrand factor OS=Homo sapiens GN=VWF PE=4 SV=1	341812	MIPARFAC	627	7.5	-	65	65	19	19
5	sp|P00739|HPTR_HUMAN	Haptoglobin-related protein OS=Homo sapiens GN=HPR PE=1 SV=2	48340	MSDLGAV	677	36.2	-	72	22	11	4
6	sp|P19652|A1AG2_HUMAN	Alpha-1-acid glycoprotein 2 OS=Homo sapiens GN=ORM2 PE=1 SV	28132	MALSWVI	61	9.5	-	7	7	2	2
7	tr|K7ERI9|K7ERI9_HUMAN	Truncated apolipoprotein C-I (Fragment) OS=Homo sapiens GN=APC	11075	MRLFLSLI	409	32.5	1	51	51	3	3
8	tr|Q5SRP5|Q5SRP5_HUMAN	Apolipoprotein M OS=Homo sapiens GN=APOM PE=1 SV=1	17751	MAAGSAF	61	17.8	1	11	11	3	3
9	sp|P35527|K1C9_HUMAN	Keratin, type I cytoskeletal 9 OS=Homo sapiens GN=KRT9 PE=1 SV=	70468	MSCRQFS	62	10.4	-	7	7	5	5
10	tr|F1C4A7|F1C4A7_HUMAN	Monocyte differentiation antigen CD14 OS=Homo sapiens GN=CD14	43702	MERASCL	185	19.2	2	18	18	6	6
11	sp|P13645|K1C10_HUMAN	Keratin, type I cytoskeletal 10 OS=Homo sapiens GN=KRT10 PE=1 S	66321	MSVRYSS	504	13.5	-	23	22	7	6
12	sp|P0C0L4|CO4A_HUMAN	Complement C4-A OS=Homo sapiens GN=C4A PE=1 SV=2	217989	MRLLWGI	18599	42	-	1660	16	65	2
13	tr|Q5J875|Q5J875_HUMAN	GUGU gamma form OS=Homo sapiens GN=GUGU PE=2 SV=1	41888	MGLFLPL	47	8.5	3	7	7	3	3
14	sp|P0C0L5|CO4B_HUMAN	Complement C4-B OS=Homo sapiens GN=C4B PE=1 SV=2	217898	MRLLWGI	19149	42	-	1692	38	65	2
15	sp|O00391|QSOX1_HUMAN	Sulfhydryl oxidase 1 OS=Homo sapiens GN=QSOX1 PE=1 SV=3	93667	MRRCNSG	373	21.3	-	61	61	16	16
16	tr|A8K6C1|A8K6C1_HUMAN	cDNA FLJ76868, highly similar to Homo sapiens cholesteryl ester tran	63358	MLAATVL	447	10.8	1	41	41	6	6
17	tr|D6REX5|D6REX5_HUMAN	Selenoprotein P (Fragment) OS=Homo sapiens GN=SEPP1 PE=4 SV=	42698	MWRSLGI	165	12.6	1	9	9	4	4
18	tr|A0A096LPE2|A0A096LPE2_	Protein SAA2-SAA4 OS=Homo sapiens GN=SAA2-SAA4 PE=4 SV=	26856	MKLLTGL	304	21.2	-	60	59	4	3
19	tr|B2R815|B2R815_HUMAN	cDNA, FLJ93695, highly similar to Homo sapiens serpin peptidase inh	57184	MHLIDYLI	167	17.1	1	25	25	7	7
20	tr|Q6U2L6|Q6U2L6_HUMAN	C4B (Fragment) OS=Homo sapiens GN=C4B PE=4 SV=1	11768	RSMQGGI	306	15	-	53	52	4	3
21	tr|A0A024R1G8|A0A024R1G8	Apolipoprotein L, 1, isoform CRA_b OS=Homo sapiens GN=APOL1	54476	MRFKSHT	561	20	11	63	63	9	9
22	tr|H6VRF8|H6VRF8_HUMAN	Keratin 1 OS=Homo sapiens GN=KRT1 PE=3 SV=1	75006	MSRQFTSI	670	24.8	4	46	36	13	11
23	sp|P05546|HEP2_HUMAN	Heparin cofactor 2 OS=Homo sapiens GN=SERPIND1 PE=1 SV=3	67548	MKHSLNA	699	28.1	-	115	115	16	16
24	tr|A0A0A0MS51|A0A0A0MS5	Gelsolin OS=Homo sapiens GN=GSN PE=4 SV=1	96753	MAEEEAL	655	18.4	6	64	64	12	12
25	tr|C9JPG5|C9JPG5_HUMAN	Semaphorin-3F OS=Homo sapiens GN=SEMA3F PE=4 SV=1	86066	MYVGSKI	40	0.9	2	5	5	1	1
26	sp|Q96PD5|PGRP2_HUMAN	N-acetylmuramoyl-L-alanine amidase OS=Homo sapiens GN=PGLYR	66703	MAQGVLV	186	8.7	-	13	13	4	4
27	tr|Q1HP67|Q1HP67_HUMAN	Lipoprotein, Lp(A) OS=Homo sapiens GN=LPA PE=1 SV=1	240494	MEHKEVV	211	6.1	1	17	17	10	10
28	tr|B3KUE5|B3KUE5_HUMAN	Phospholipid transfer protein, isoform CRA_c OS=Homo sapiens GN=	62359	MGLSGSD	390	18.3	2	33	33	9	9
29	sp|P40197|GPV_HUMAN	Platelet glycoprotein V OS=Homo sapiens GN=GP5 PE=1 SV=1	65693	MLRGTLL	67	13.2	-	10	10	6	6
30	sp|P08697|A2AP_HUMAN	Alpha-2-antiplasmin OS=Homo sapiens GN=SERPINF2 PE=1 SV=3	60957	MALLWGI	421	17.7	-	62	62	8	8
31	tr|V9GYG9|V9GYG9_HUMAN	Apolipoprotein A-II (Fragment) OS=Homo sapiens GN=APOA2 PE=	14103	MKLLAAT	186	38.9	2	60	60	3	3
32	tr|B2R5S1|B2R5S1_HUMAN	cDNA, FLJ92595, highly similar to Homo sapiens angiotensinogen (se	60156	MRKRAPC	350	9.5	6	15	15	4	4
33	sp|P02763|A1AG1_HUMAN	Alpha-1-acid glycoprotein 1 OS=Homo sapiens GN=ORM1 PE=1 SV	28288	MALSWVI	54	7.5	-	5	5	1	1
34	tr|C0JYY2|C0JYY2_HUMAN	Apolipoprotein B (Including Ag(X) antigen) OS=Homo sapiens GN=A	625263	MDPPRPA	22843	45.3	-	2373	1080	198	94
35	tr|D3DNU8|D3DNU8_HUMAN	Kininogen 1, isoform CRA_a OS=Homo sapiens GN=KNG1 PE=4 SV	59249	MKLITILFI	208	25.1	3	31	31	10	10
36	sp|Q92954|PRG4_HUMAN	Proteoglycan 4 OS=Homo sapiens GN=PRG4 PE=1 SV=2 >tr|A0A02	202736	MAWKTLI	94	4.5	-	12	12	7	7
37	sp|P55056|APOC4_HUMAN	Apolipoprotein C-IV OS=Homo sapiens GN=APOC4 PE=1 SV=1	17623	MSLLRNR	288	20.5	-	35	35	3	3
38	tr|Q59HB3|Q59HB3_HUMAN	Apolipoprotein B variant (Fragment) OS=Homo sapiens PE=2 SV=1	222441	TIEGPLTS	7264	45	-	713	7	67	1
39	tr|J3KPY9|J3KPY9_HUMAN	Anthrax toxin receptor 2 OS=Homo sapiens GN=ANTXR2 PE=1 SV=	54148	MRLSFIVF	65	16.3	1	8	7	6	5
1	sp|P05164|PERM_HUMAN	Myeloperoxidase OS=Homo sapiens GN=MPO PE=1 SV=1	91781	MGVPFFS	129	8.5	-	15	8	6	4
2	tr|Q9UMV1|Q9UMV1_HUMA	Complement C4B1a (Fragment) OS=Homo sapiens GN=C4B PE=4 S	5960	AAIIESPG	102	23.9	1	14	14	1	1
3	sp|Q15485|FCN2_HUMAN	Ficolin-2 OS=Homo sapiens GN=FCN2 PE=1 SV=2	39607	MELDRAV	308	14.7	-	38	38	5	5
4	tr|Q6UXM4|Q6UXM4_HUMA	Ficolin (Collagen/fibrinogen domain containing) 3 (Hakata antigen), is	34547	MDLLWIL	731	26.4	1	80	80	8	8
5	tr|E7EUT5|E7EUT5_HUMAN	Glyceraldehyde-3-phosphate dehydrogenase OS=Homo sapiens GN=G	34412	MEEMRDI	175	18.5	1	11	11	3	3
6	tr|F8VV32|F8VV32_HUMAN	Lysozyme C OS=Homo sapiens GN=LYZ PE=1 SV=1	13895	MKALIVL	163	23.1	1	12	12	2	2
7	tr|Q5VY30|Q5VY30_HUMAN	Retinol binding protein 4, plasma, isoform CRA_b OS=Homo sapiens	26952	MNYSKIP	63	15.1	1	7	7	3	3
8	tr|E7EQB2|E7EQB2_HUMAN	Lactotransferrin (Fragment) OS=Homo sapiens GN=LTF PE=1 SV=1	92148	MGLCLAC	602	30.2	2	52	52	18	18
9	sp|Q02985|FHR3_HUMAN	Complement factor H-related protein 3 OS=Homo sapiens GN=CFHR	44580	MLLLINVI	81	14.5	-	11	10	5	4
10	tr|B3KPS3|B3KPS3_HUMAN	cDNA FLJ32131 fis, clone PEBLM2000267, highly similar to Tubulin	52809	MPSDKTIC	100	15.4	8	7	3	4	2
11	tr|B2R6W1|B2R6W1_HUMAN	cDNA, FLJ93143, highly similar to Homo sapiens complement compo	111585	MKVISLFI	635	25.9	-	91	4	19	2
12	sp|Q03591|FHR1_HUMAN	Complement factor H-related protein 1 OS=Homo sapiens GN=CFHR	43938	MWLLVSV	957	33	-	97	20	10	2
13	tr|B0YIW1|B0YIW1_HUMAN	Apolipoprotein A-V variant 3 OS=Homo sapiens GN=APOA5 PE=2 S	45822	MAAVLTV	228	19.8	2	22	22	7	7
14	tr|A0A024RDB8|A0A024RDB8	Heparanase, isoform CRA_a OS=Homo sapiens GN=HPSE PE=4 SV=	73287	MLLRSKP	105	6.6	2	14	14	4	4
15	sp|Q07954|LRP1_HUMAN	Prolow-density lipoprotein receptor-related protein 1 OS=Homo sapien	576082	MLTPPLLI	243	3.5	-	29	29	14	14
16	tr|B2R8I2|B2R8I2_HUMAN	cDNA, FLJ93914, highly similar to Homo sapiens histidine-rich glyco	67498	MKALIAA	211	22.1	1	29	29	11	11
17	sp|O00602|FCN1_HUMAN	Ficolin-1 OS=Homo sapiens GN=FCN1 PE=1 SV=2	42205	MELSGAT	99	8.9	-	9	9	3	3
18	tr|A0A075B6R9|A0A075B6R9_	Protein IGKV2D-24 (Fragment) OS=Homo sapiens GN=IGKV2D-24	14040	MRLLAQL	160	16.7	-	8	7	2	1
19	tr|D3DQX7|D3DQX7_HUMAN	Serum amyloid A protein OS=Homo sapiens GN=SAA1 PE=3 SV=1	15436	MKLLTGL	105	20.5	-	10	9	3	2
20	sp|P07357|CO8A_HUMAN	Complement component C8 alpha chain OS=Homo sapiens GN=C8A	76870	MFAVVFF	244	15.2	-	28	28	8	8
21	tr|B2R6M6|B2R6M6_HUMAN	cDNA, FLJ93024, highly similar to Homo sapiens EGF-containing fib	60522	MLKALFL	224	9.7	6	22	22	4	4
22	tr|A0A5E4|A0A5E4_HUMAN	Uncharacterized protein OS=Homo sapiens PE=2 SV=1	28363	MAWMMI	1459	35.3	-	173	14	7	1
23	sp|Q13103|SPP24_HUMAN	Secreted phosphoprotein 24 OS=Homo sapiens GN=SPP2 PE=1 SV=1	27041	MISRMEK	138	15.6	-	14	14	3	3
24	tr|B4E335|B4E335_HUMAN	cDNA FLJ52842, highly similar to Actin, cytoplasmic 1 OS=Homo sa	45627	MDDDIAA	366	33.6	7	42	42	9	9
25	tr|X6RLJ0|X6RLJ0_HUMAN	Complement C1q subcomponent subunit A (Fragment) OS=Homo sap	27065	MEGPRGV	58	11.8	1	8	8	3	3
26	tr|B4DNS6|B4DNS6_HUMAN	cDNA FLJ54278, highly similar to SPARC-like protein 1 OS=Homo s	71190	MKTGLFF	116	9.6	4	13	13	4	4
27	tr|A0A087WT59|A0A087WT5	Transthyretin OS=Homo sapiens GN=TTR PE=3 SV=1	23099	MASHRLL	813	21.1	4	56	56	5	5
28	tr|A0A024R962|A0A024R962_	HCG40889, isoform CRA_b OS=Homo sapiens GN=hCG_40889 PE=	168295	MRLLAKII	2249	30.5	1	277	12	33	2
29	tr|Q6MZL2|Q6MZL2_HUMAN	Putative uncharacterized protein DKFZp686M0562 (Fragment) OS=H	38922	NPGIYTCS	103	12.8	-	10	9	4	3
30	tr|D6RHH7|D6RHH7_HUMAN	Corticotropin-releasing factor-binding protein OS=Homo sapiens GN=	30563	MSPNFKL	48	8	1	4	4	2	2
31	sp|P48740|MASP1_HUMAN	Mannan-binding lectin serine protease 1 OS=Homo sapiens GN=MAS	92409	MRWLLLY	566	26	-	59	15	14	6

A-VS-B@F	A-VS-B@F	A-VS-B@C	A-VS-B@C	A-VS-B@S	A-VS-B@S	A-VS-B@M	A-VS-B@S	KEGG(ko_i	GO Biologi	GO Molecu	GO Cellular_Component
0.608	0.607	4	4	*	*	0.6075	0.000707	K07605|1|0	GO:003298	GO:000520	GO:0005794//Golgi apparatus;GO:0045095//keratin filament
0.562	0.549	8	8	-	*	0.5555	0.009192	K06238|1|0	GO:000741	GO:000486	GO:0030133//transport vesicle;GO:0005615//extracellular space;GO:0005788//endopl
0.794	0.784	106	106	*	-	0.789	0.007071	K05399|1|0	GO:003272	GO:000510	GO:0005615//extracellular space
0.813	0.665	31	31	*	-	0.739	0.104652	K03900|1|0	GO:003016	GO:004748	GO:0009897//external side of plasma membrane;GO:0033093//Weibel-Palade body;G
0.676	0.704	18	18	*	*	0.69	0.019799	K14477|1|0	GO:000695	GO:003049	GO:0031838//haptoglobin-hemoglobin complex;GO:0034366//spherical high-density li
0.897	0.763	3	3	-	*	0.83	0.094752	-	GO:000268	GO:000551	GO:0005615//extracellular space
0.739	0.71	14	14	*	-	0.7245	0.020506	K04524|1|9	GO:001087	GO:003121	GO:0042627//chylomicron;GO:0034361//very-low-density lipoprotein particle;GO:003
0.669	0.73	2	2	-	*	0.6995	0.043134	-	GO:003334	GO:000554	GO:0034362//low-density lipoprotein particle;GO:0034365//discoidal high-density lipo
0.63	0.618	3	3	*	-	0.624	0.008485	K07604|1|1	GO:000728	GO:000520	GO:0045095//keratin filament;GO:0048471//perinuclear region of cytoplasm
0.594	0.483	11	11	*	*	0.5385	0.078489	K04391|1|0	GO:000690	GO:007089	GO:0045121//membrane raft;GO:0005615//extracellular space;GO:0031225//anchored
0.468	0.442	14	14	*	*	0.455	0.018385	K07604|1|0	GO:007127	GO:003028	GO:0005737//cytoplasm;GO:0045095//keratin filament
0.79	0.622	5	5	*	*	0.706	0.118794	K03989|1|0	GO:000695	GO:000486	GO:0005615//extracellular space;GO:0005886//plasma membrane
0.264	0.215	3	3	*	*	0.2395	0.034648	K03898|1|8	-	-	GO:0005576//extracellular region
0.844	0.653	29	29	-	*	0.7485	0.135057	K03989|1|0	GO:000695	GO:000486	GO:0005615//extracellular space;GO:0005886//plasma membrane
0.816	0.801	19	18	*	*	0.8085	0.010607	K10758|1|0	GO:004545	GO:000905	GO:0005615//extracellular space;GO:0030173//integral to Golgi membrane
0.721	0.829	17	17	*	*	0.775	0.076368	K05399|1|1	GO:004369	GO:001712	GO:0031982//vesicle;GO:0034364//high-density lipoprotein particle
0.731	0.894	5	4	*	*	0.8125	0.115258	-	GO:001026	GO:000843	GO:0005615//extracellular space
0.759	0.724	19	19	*	-	0.7415	0.024749	-	GO:000695	-	GO:0034364//high-density lipoprotein particle
0.774	0.751	11	11	*	*	0.7625	0.016263	K04525|1|1	GO:001095	GO:000486	GO:0005615//extracellular space
0.744	0.707	10	10	*	*	0.7255	0.026163	K03989|1|9	GO:004508	-	GO:0005576//extracellular region;GO:0005886//plasma membrane
0.359	0.325	32	32	*	*	0.342	0.024042	K14480|1|0	GO:001983	GO:000828	GO:0031224//intrinsic to membrane;GO:0034364//high-density lipoprotein particle;G
0.504	0.562	21	22	*	-	0.533	0.041012	K07605|1|0	GO:000697	GO:000487	GO:0045095//keratin filament;GO:0005886//plasma membrane
0.473	0.441	33	33	*	-	0.457	0.022627	K03912|1|0	GO:000693	GO:000486	GO:0005576//extracellular region
0.678	0.645	34	34	*	*	0.6615	0.023335	K05768|1|0	GO:005159	GO:000550	GO:0030027//lamellipodium;GO:0015629//actin cytoskeleton;GO:0005615//extracellu
0.627	0.636	5	5	*	*	0.6315	0.006364	K06840|1|0	GO:004884	GO:000487	GO:0005615//extracellular space;GO:0016020//membrane
0.813	0.694	7	7	*	-	0.7535	0.084146	K01446|1|0	GO:003282	GO:000827	GO:0005622//intracellular;GO:0005576//extracellular region;GO:0016020//membrane
0.451	0.452	9	9	-	*	0.4515	0.000707	K09644|1|0	GO:003016	GO:000425	GO:0031093//platelet alpha granule lumen;GO:0034358//plasma lipoprotein particle;G
0.438	0.444	21	21	*	*	0.441	0.004243	K08761|1|0	GO:001018	GO:000828	GO:0005576//extracellular region
0.68	0.474	4	4	*	*	0.577	0.145664	K06260|1|0	GO:000716	GO:000551	GO:0005887//integral to plasma membrane;GO:0009986//cell surface
0.94	0.815	18	18	-	*	0.8775	0.088388	K03983|1|0	GO:000203	GO:004280	GO:0005577//fibrinogen complex;GO:0031093//platelet alpha granule lumen
0.603	0.615	10	10	*	*	0.609	0.008485	K08758|1|2	GO:006069	GO:004280	GO:0042627//chylomicron;GO:0005788//endoplasmic reticulum lumen;GO:0034361//
0.857	0.825	11	11	-	*	0.841	0.022627	K09821|1|0	GO:004816	GO:000808	GO:0005615//extracellular space;GO:0044444//cytoplasmic part
0.797	0.895	5	5	*	-	0.846	0.069296	-	GO:000268	GO:000551	GO:0005615//extracellular space
0.397	0.395	519	519	*	-	0.396	0.001414	K14462|1|0	GO:001088	GO:005075	GO:0030669//clathrin-coated endocytic vesicle membrane;GO:0034360//chylomicron r
0.835	0.757	12	12	-	*	0.796	0.055154	K03898|1|0	GO:003016	GO:000510	GO:0031093//platelet alpha granule lumen;GO:0005615//extracellular space;GO:0005
0.695	0.696	3	4	*	-	0.6955	0.000707	K06251|1|3	GO:000695	GO:003024	GO:0005576//extracellular region;GO:0016021//integral to membrane
0.708	0.668	15	14	*	*	0.688	0.028284	-	GO:000662	GO:000531	GO:0034364//high-density lipoprotein particle;GO:0034361//very-low-density lipoprot
0.468	0.416	7	7	*	*	0.442	0.03677	K14462|1|0	GO:001088	GO:005075	GO:0030669//clathrin-coated endocytic vesicle membrane;GO:0034360//chylomicron r
0.535	0.454	3	3	*	*	0.4945	0.057276	-	GO:002241	GO:000487	GO:0005789//endoplasmic reticulum membrane;GO:0005886//plasma membrane;GO:
1.748	1.988	6	6	*	*	1.868	0.169706	K10789|1|0	GO:000695	GO:004687	GO:0005764//lysosome;GO:0030141//secretory granule;GO:0005615//extracellular sp
2.168	1.75	7	7	*	*	1.959	0.295571	K03989|1|3	GO:000695	GO:000486	GO:0005615//extracellular space;GO:0005886//plasma membrane
2.859	2.981	14	14	*	*	2.92	0.086267	K10104|1|7	GO:000822	GO:000382	GO:0005615//extracellular space;GO:0005581//collagen
1.18	1.258	48	48	*	*	1.219	0.055154	K10104|1|2	GO:000716	GO:003024	GO:0005615//extracellular space;GO:0005581//collagen
1.515	1.089	8	8	*	-	1.302	0.301227	K00134|1|3	GO:005140	GO:005128	GO:0005829//cytosol;GO:0015630//microtubule cytoskeleton;GO:0048471//perinucle
1.512	1.505	7	7	*	*	1.5085	0.00495	K13915|1|5	GO:004274	GO:000379	GO:0005615//extracellular space
2.028	1.684	4	4	*	*	1.856	0.243245	-	GO:000609	GO:003463	GO:0005615//extracellular space
2.479	2.434	26	26	*	*	2.4565	0.03182	K14736|1|0	GO:004274	GO:000819	GO:0030141//secretory granule;GO:0097013//phagocytic vesicle lumen;GO:0005576//
1.233	1.352	4	4	-	*	1.2925	0.084146	K04004|1|8	GO:000686	GO:000531	GO:0005615//extracellular space
1.209	1.279	3	3	-	*	1.244	0.049497	K07374|1|0	GO:005108	GO:001990	GO:0030496//midbody;GO:0005881//cytoplasmic microtubule
1.218	1.098	2	2	*	-	1.158	0.084853	K03996|1|0	GO:000695	-	GO:0005579//membrane attack complex;GO:0005576//extracellular region
1.343	1.296	10	10	*	-	1.3195	0.033234	K04004|1|6	GO:004508	-	GO:0005615//extracellular space
1.378	1.211	9	9	*	*	1.2945	0.118087	K09025|1|0	GO:004572	GO:005075	GO:0042627//chylomicron;GO:0034361//very-low-density lipoprotein particle;GO:003
1.427	1.471	4	4	*	*	1.449	0.031113	K07964|1|0	GO:003369	GO:000456	GO:0045121//membrane raft;GO:0005765//lysosomal membrane;GO:0005576//extrac
1.193	1.208	16	16	-	*	1.2005	0.010607	K04550|1|0	GO:004295	GO:000504	GO:0005905//coated pit;GO:0030425//dendrite;GO:0043025//neuronal cell body;GO:
1.28	1.223	18	18	-	*	1.2515	0.040305	-	GO:004306	GO:000486	GO:0005886//plasma membrane;GO:0031093//platelet alpha granule lumen;GO:0005
1.321	1.366	4	5	*	*	1.3435	0.03182	K10104|1|0	GO:000822	GO:000382	GO:0005615//extracellular space;GO:0005581//collagen
1.346	1.158	6	6	*	*	1.252	0.132936	K06554|1|4	-	-	
1.459	1.048	7	7	*	-	1.2535	0.290621	-	GO:003016	GO:000166	GO:0034364//high-density lipoprotein particle
1.216	1.033	10	10	*	-	1.1245	0.129401	K03997|1|0	GO:000695	-	GO:0005615//extracellular space;GO:0005579//membrane attack complex
1.206	1.336	7	6	*	*	1.271	0.091924	K08023|1|1	GO:001810	GO:000550	GO:0005615//extracellular space;GO:0005578//proteinaceous extracellular matrix
1.594	1.193	3	3	*	-	1.3935	0.28355	K06554|1|1	-	-	
1.208	1.231	5	5	*	*	1.2195	0.016263	-	GO:000646	GO:000486	GO:0043234//protein complex;GO:0005576//extracellular region
1.33	1.27	22	22	*	*	1.3	0.042426	K05692|1|0	GO:000741	GO:001990	GO:0030863//cortical cytoskeleton;GO:0031941//filamentous actin;GO:0005829//cyto
1.178	1.254	2	2	-	*	1.216	0.05374	K03986|1|3	GO:004508	GO:000551	GO:0005602//complement component C1 complex;GO:0005581//collagen
1.336	1.124	8	8	*	-	1.23	0.149907	K08136|1|2	GO:000716	GO:000550	GO:0005615//extracellular space;GO:0005578//proteinaceous extracellular matrix
1.202	1.129	45	45	*	-	1.1655	0.051619	K07127|1|1	-	GO:000551	GO:0005576//extracellular region
1.072	1.227	8	8	-	*	1.1495	0.109602	K04004|1|0	GO:000695	-	GO:0005615//extracellular space
1.362	1.527	4	4	*	*	1.4445	0.116673	K03992|1|0	GO:000650	GO:000550	GO:0005615//extracellular space
1.539	1.112	3	3	*	-	1.3255	0.301935	-	GO:007127	GO:000551	GO:0031045//dense core granule;GO:0043196//varicosity;GO:0043679//axon terminu
1.306	1.34	9	9	-	*	1.323	0.024042	K03992|1|0	GO:000650	GO:000550	GO:0005615//extracellular space
